# Evaluating the effect of spaceflight on the host–pathogen interaction between human intestinal epithelial cells and *Salmonella* Typhimurium

**DOI:** 10.1038/s41526-021-00136-w

**Published:** 2021-03-09

**Authors:** Jennifer Barrila, Shameema F. Sarker, Nicole Hansmeier, Shanshan Yang, Kristina Buss, Natalia Briones, Jin Park, Richard R. Davis, Rebecca J. Forsyth, C. Mark Ott, Kevin Sato, Cristine Kosnik, Anthony Yang, Cheryl Shimoda, Nicole Rayl, Diana Ly, Aaron Landenberger, Stephanie D. Wilson, Naoko Yamazaki, Jason Steel, Camila Montano, Rolf U. Halden, Tom Cannon, Sarah L. Castro-Wallace, Cheryl A. Nickerson

**Affiliations:** 1 Biodesign Center for Fundamental and Applied Microbiomics, Arizona State University, Tempe, AZ USA; 2 Biodesign Center for Infectious Diseases and Vaccinology, Arizona State University, Tempe, AZ USA; 3 Swette Center for Environmental Biotechnology, The Biodesign Institute, Arizona State University, Tempe, AZ USA; 4 Luther College at University of Regina, Department of Biology, Regina, Saskatchewan Canada; 5 Bioinformatics Core Facility, Bioscience, Knowledge Enterprise, Arizona State University, Tempe, AZ USA; 6 Integrated Cancer Genomics Division, The Translational Genomics Research Institute, Phoenix, AZ USA; 7 Biomedical Research and Environmental Sciences Division, NASA Johnson Space Center, Houston, TX USA; 8 NASA Ames Research Center, Mountain View, CA USA; 9 Tissue Genesis, Inc, Honolulu, HI USA; 10 NASA Headquarters, Washington, D.C. USA; 11 DoD Space Test Program, Houston, TX USA; 12 Astronaut Office, NASA Johnson Space Center, Houston, TX USA; 13 Space Biomedical Research Office, Human Space Technology and Astronauts Department, Japan Aerospace Exploration Agency (JAXA), Tokyo, Japan; 14 Biodesign Center for Environmental Health Engineering, School of Sustainable Engineering and the Built Environment, Arizona State University, Tempe, AZ USA; 15 School of Life Sciences, Arizona State University, Tempe, AZ USA

**Keywords:** Microbiology

## Abstract

Spaceflight uniquely alters the physiology of both human cells and microbial pathogens, stimulating cellular and molecular changes directly relevant to infectious disease. However, the influence of this environment on host–pathogen interactions remains poorly understood. Here we report our results from the STL-IMMUNE study flown aboard Space Shuttle mission STS-131, which investigated multi-omic responses (transcriptomic, proteomic) of human intestinal epithelial cells to infection with *Salmonella* Typhimurium when both host and pathogen were simultaneously exposed to spaceflight. To our knowledge, this was the first in-flight infection and dual RNA-seq analysis using human cells.

Humans will travel to space more frequently and for longer duration as the rapidly growing spaceflight industry enables more routine access for both professional astronauts and the public. Spaceflight can adversely affect multiple aspects of human physiology, including immune and gastrointestinal function^1–4^. While pre-flight screening and quarantine procedures have reduced infection incidence, astronauts and cargo can still harbor opportunistic and obligate pathogens, including *Salmonella* spp., which have been recovered from both crew refuse and International Space Station (ISS) surfaces^5–8^. We previously discovered that spaceflight and spaceflight analog culture increased the virulence and stress resistance of the foodborne pathogen, *Salmonella enterica* serovar Typhimurium (*Salmonella*)^9–12^. These environments have also been shown to increase the virulence and/or alter pathogenesis-related phenotypes of other microorganisms^13–16^. Collectively, these findings indicate an increased risk for infection during space travel and underscore the need for a more in-depth understanding of the similarities and differences between spaceflight and ground-based infection mechanisms.

Studies performed in spaceflight and spaceflight analog environments have revealed unexpected physiological responses in both mammalian and microbial cells^14,15^. This is not surprising given that physical forces in the cellular microenvironment (including those associated with microgravity) profoundly alter both host and pathogen phenotypes through mechanotransduction^17^. An integrated network involving the cytoskeleton, cell surface receptors, and the extracellular matrix (ECM) mediates the dynamic three-dimensional (3D) interactions between host cells and their external environment to regulate cellular responses^17–19^. Since spaceflight alters the organization and/or expression of these classes of proteins^14,20,21^, it is logical to predict that this environment could impact infection processes for invasive pathogens like *Salmonella* that hijack the host and actively exploit these structural networks to enable their invasion and dissemination^22,23^.

From the pathogen perspective, we previously discovered that spaceflight analog culture of *Salmonella* in the Rotating Wall Vessel bioreactor led to increased virulence in mice^24^, enhanced stress resistance^9–11,24^, global transcriptional and proteomic reprogramming^11,12,24^ and enhanced the colonization of a human 3D intestinal co-culture model (JB, SY, RRD, RJF, CMO, JS, CAN; manuscript in preparation). Space Shuttle experiments aboard STS-115 and STS-123 independently confirmed RWV trends by demonstrating that spaceflight increased the virulence and globally altered the transcriptomic and proteomic responses of *Salmonella* relative to identical ground controls^9,10^. Supplementation of select ions in the media was able to counteract this spaceflight-associated increased virulence^10^. It is important to note that *Salmonella* virulence studies were performed when the bacteria were cultured in spaceflight, brought back to Earth and immediately used in animal infections (i.e., the host was not exposed to spaceflight). Transcriptional profiling revealed a central role for the RNA-binding protein Hfq in the response of *Salmonella* to both spaceflight and spaceflight analog culture in the RWV^9,10^ and subsequent studies indicated altered expression of *hfq* or the Hfq regulon in other microbes^25–28^.

In this study, we investigated how spaceflight culture alters the response of human intestinal epithelial cells (HT-29) to infection by *Salmonella* when both the host and pathogen were exposed to spaceflight. The experiment was flown as part of the Space Tissue Loss (STL) payload and operationally designated as STL-IMMUNE, since epithelial cells are excellent sentinels of innate immunity by providing the first physical and chemical barriers to infection and coordinating additional innate and mucosal defenses and subsequent adaptive responses^29,30^. Since in-flight technical limitations precluded the use of gentamicin protection assays to quantify bacterial invasion into the epithelium, we focused our analysis on transcriptomic (dual RNA-seq of host and pathogen) and proteomic profiling (Isobaric tags for relative and absolute quantification/iTRAQ). These global analyses can yield valuable insight into the status of healthy and infected cells. Figure 1 shows the experimental design and timeline for STL-IMMUNE and schematic of hollow fiber bioreactors used for 3D cell culture. HT-29 cells were seeded into the extracapillary space (ECS) of hollow fiber bioreactors which contained collagen I-coated capillary fibers for intestinal cell attachment. Oxygenated and CO_2_-containing media was continually perfused through the intracapillary space (ICS) of the fibers (underneath the basal side of the cells, which also prevented exposure of cells to high fluid shear forces). During spaceflight, *Salmonella* was introduced to the apical side of HT-29 cultures through ECS ports and allowed to incubate for 6 h before fixative was perfused through the ICS.

**Fig. 1:**
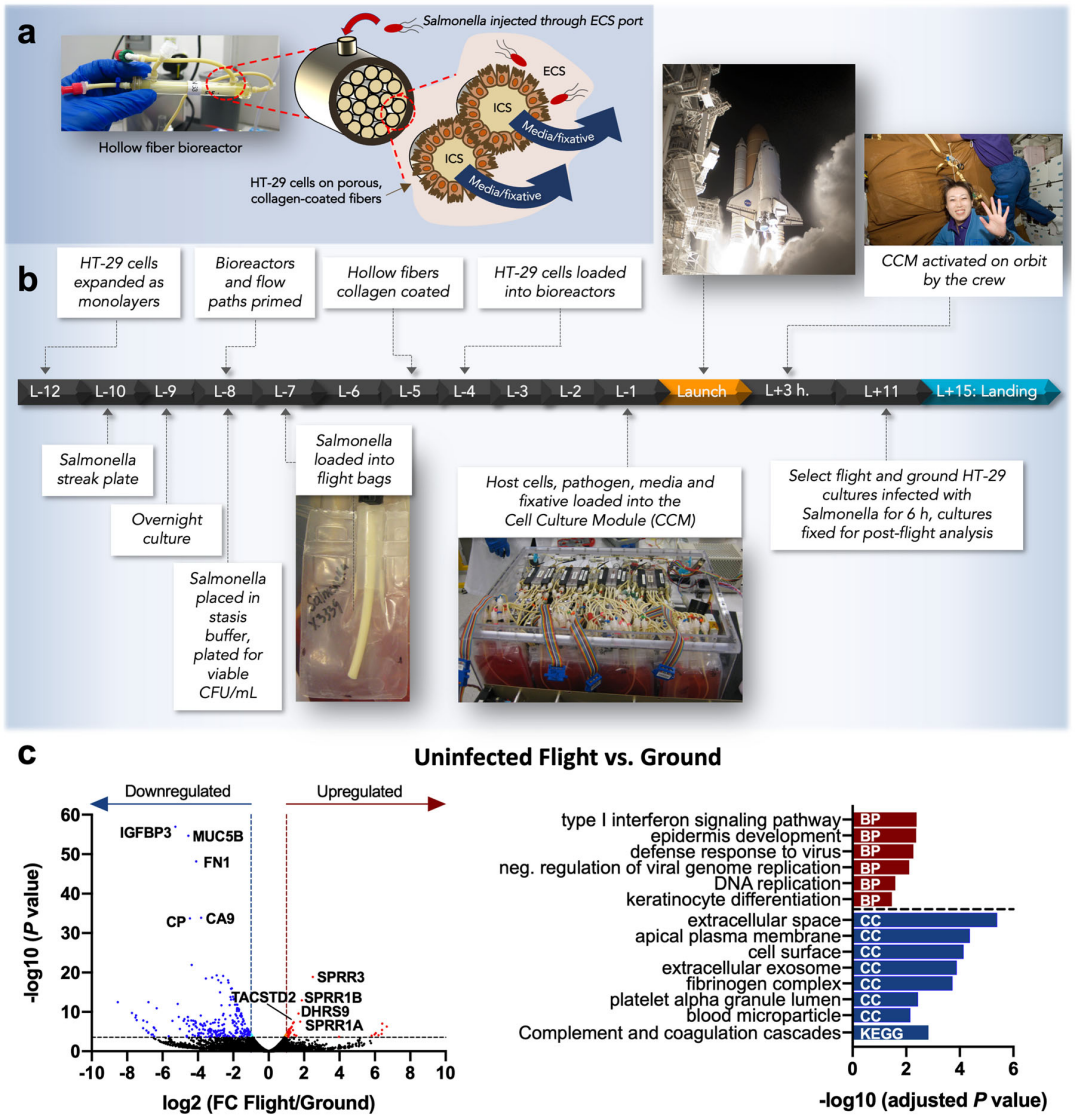
STL-IMMUNE experimental design and impact of spaceflight on uninfected HT-29 transcriptional profiles. **a** Hollow fiber bioreactor technology. Photo and cross-sectional schematic of the hollow fiber bioreactors used in this study. Hollow fibers had a pore size of 0.3 µm to retain bacteria within the extracapillary space (ECS) while still facilitating nutrient and gas exchange via diffusion as oxygenated media was perfused through the intracapillary space (ICS). **b** STL-IMMUNE experimental design. HT-29 cells and bacteria were prepared as shown (see “Methods” section for additional detail). Bioreactors, bacteria, and fixative bags were integrated into the CCM hardware and launched aboard Space Shuttle Discovery mission STS-131. Crew members activated the CCM once on orbit. At mission elapsed day 11, cultures were infected with *S*. Typhimurium for 6 h and fixed for postflight analysis. Launch and astronaut images: NASA [STS-131-S-036 (image is slightly cropped), S131E007206, and S131E007188]. **c** Spaceflight-induced transcriptional responses of HT-29 cells relative to ground controls. Left panel: volcano plot depicting differentially expressed genes (DEGs) between flight and ground control cultures (flight/ground). Red: significantly upregulated genes according to a log_2_ fold change in expression of at least ± 1 and FDR < 0.05. Blue: significantly downregulated genes. The top 5 significantly up and downregulated genes are labeled. Right panel: Analysis of upregulated (red) and downregulated (blue) transcripts revealed significantly enriched GO terms and KEGG pathways using DAVID. BP biological process, CC cellular component. The authors affirm that informed consent for publication of the images in Fig. 1 was provided.

Between uninfected flight and ground HT-29 cultures, RNA-seq profiling showed significant differences in 307 genes (≥2-fold change; false discovery rate (FDR) < 0.05; Fig. 1c and Supplementary Table 1). Evaluation of the top 5 significantly upregulated genes revealed the enhanced expression of several transcripts belonging to the small proline-rich (SPRR) family as well as *DHRS9* (a retinol dehydrogenase) and tumor-associated calcium signal transducer 2 (Fig. 1c). *SPRR* genes encode mechanosensitive proteins that play roles in barrier function, differentiation, reactive oxygen species (ROS) detoxification, wound healing and cancer^31^. They are also likely involved in the stabilization of the actin cytoskeleton in response to biomechanical stress^32^. Their expression is regulated by ECM/integrin interactions and by various stimuli, including UV radiation, inflammation, oxidative stress, DNA damage, and retinoids^31,33^. Gene ontology (GO) analysis of upregulated genes indicated enrichment in type I interferon (IFN-I) signaling/antiviral defense, DNA replication, and differentiation (including the SPRR genes) (Fig. 1c, Supplementary Table 2). The upregulation of viral defense genes was also previously observed in the livers of mice following spaceflight^34^.

IFN-I signaling is complex and context dependent, regulating a broad range of cellular processes^35,36^. In addition to modulating innate and adaptive immune defenses against pathogens, IFN-I regulates intestinal homeostasis, including epithelial cell proliferation, and barrier function^36,37^. IFN-I signaling is often associated with decreased proliferation to promote differentiation, cellular repair, or defense against infection^36,37^. We found that genes important for glycolysis (*SLC2A3*, *HK2*, *PFKFB3, PFKFB4, PDK1)* and fatty acid metabolism (*FADS1, FADS2, ELOVL5, EHHADH*, *SIRT4*) were downregulated in flight (Supplementary Table 1). Previous spaceflight studies using either cultured macrophages^38^ or murine liver tissue^34^ also saw the suppression of multiple glycolytic enzymes, with the latter study postulating a model linking spaceflight changes in cellular metabolism, ROS, and immune dysregulation^34^. The downregulation of these bioenergetic pathways in our study could indicate the reduced proliferation of the flight cultures, which could result from a number of phenotypes, including decreased tumorigenicity/increased differentiation, increased DNA damage or apoptosis. During cancer metabolism, glycolysis and fatty acid synthesis is often elevated^39^. Several genes highly expressed in tumors or undifferentiated cells were also downregulated in flight, including *CA9, CA12, CEACAM6, ITGA5, CLDN2*, *COL5A2*, *VIM*, *PROM1*, and *LGR4* (Supplemental Table 1). Together, these trends may indicate decreased tumorigenicity of the flight cells. *Salmonella* is known to preferentially colonize cancer cells^40^, thus differences in tumorigenicity between flight and ground cultures could impact infection. Of relevance, we previously showed that RWV culture of HT-29 cells induced a more normal, differentiated phenotype relative to controls^41^. Although in our present study we saw indicators of increased differentiation of the flight cultures, there were also some potential indicators of increased tumorigenicity (e.g., downregulation of *VIL*, *TP53I11*, upregulation of *CCAT1;* Supplemental Table 1). In addition, LGR4 is also important for Paneth cell differentiation^42^. A reduction in Paneth cells could diminish antimicrobial defenses. Host metabolism is also important in the context of an impending infection as the pathogen must scavenge for available nutrients^43,44^. Decreases in intracellular glucose could dampen intracellular pathogen replication depending on the availability of other nutrients. However, *Salmonella* has evolved an impressive metabolic plasticity to exploit diverse nutrients in parallel, even at trace levels^44^.

Engagement of IFN-I signaling and decreased expression of metabolic genes could also indicate increased apoptosis. However, pro-apoptotic genes *BNIP3* and *BNIP3L* were downregulated in flight, suggesting reduced apoptosis. A previous spaceflight study^45^ showed reduced apoptosis and increased differentiation in MIP-101 colon cells, as well as increased expression of carcinoembryonic antigen proteins (often upregulated in colon cancer). In our study, we observed the downregulation of *CEACAM6* (Supplementary Table 1). IFN-I signaling can also be activated by the DNA damage response following exposure to radiation, oxidative stress, or other harmful stimuli. DNA damage can occur during spaceflight due to radiation exposure and oxidative stress, including in the gastrointestinal tract^46–49^. Chromosomal damage has been observed in astronauts^48,49^ and we previously saw the downregulation of select DNA repair genes in astronaut blood cells^50^, which could potentially impede repair. In our present study, several genes involved in checkpoint-mediated responses to DNA damage and replication stress were upregulated (*BRCA1*, *CLSPN, MCM10, RRM2*, and *KIAA0101)*. Thus, it is also possible that changes in IFN-I and metabolic genes could be due to DNA damage caused by oxidative stress and/or radiation exposure.

GO analysis of downregulated genes indicated enrichment in cellular components related to the cell surface/membrane and extracellular components as well as complement and coagulation pathways (Fig. 1c, Supplementary Table 2). Decreased expression of genes encoding factors classically associated with blood or other tissues was observed, including complement and fibrinogen complex proteins (e.g., *C3, FGG, FGA*, and *FGB*) (Supplementary Table 2). Fibrinogen and complement proteins are also expressed by epithelial cells and play an important role in infection^51–54^. However, as there are cases where RNA from fetal bovine serum (FBS) in culture media has been detected in mRNA analyses^55^, we cannot exclude the possibility of FBS interference. Importantly, the same batch of heat-inactivated FBS was used for identical and simultaneous preparation of all flight and ground cultures in this study.

We also performed iTRAQ analysis to obtain a snapshot of the cellular proteomic profile in the uninfected flight cultures compared to ground controls. The presence of FBS in the culture media resulted in strong interference from bovine proteins; a subset of which could not be distinguished from human proteins due to their identical sequence homologies and thus were excluded from our analysis. Despite this challenge, we observed flight-associated upregulation of the major cytoskeletal proteins G-actin and tubulin, which are known targets of *Salmonella* effectors to facilitate invasion^56^ (Supplementary Table 3). HSPA1A protein was also 2.4-fold upregulated in flight. None of these proteins were differentially regulated at the transcriptional level; however, it is fairly common to see divergence in mRNA and protein expression due to a variety of biological factors, such as alterations in posttranscriptional modifications, translation efficiency and RNA or protein stabilities (reviewed by Maier et al.)^57^.

We then examined the data for the infections performed with *Salmonella* during spaceflight and in our ground controls. The experiment was designed to automatically inject 1 mL bacterial inoculum (~1 × 10^7^ colony forming units (CFU)) into HT-29 cultures. Postlanding hardware inspection revealed that all flight samples received the intended volume of bacteria; however, ground controls received slightly different volumes (see “Methods” section). The 6-h incubation in the absence of gentamicin would have allowed sufficient time for the bacteria to replicate in the media during attachment and invasion, but it is unknown whether differences in initial inoculum eventually equalized. In addition, while we had sufficient material to perform iTRAQ analysis using triplicate cultures, only one infected flight sample had enough material remaining after proteomics for RNA-seq, thus we analyzed multiple technical replicates from a single flight bioreactor for that condition (see “Methods” section). Acknowledging these limitations, given the rare nature of these samples due to the inherent difficulty in performing spaceflight infections, we present a preliminary analysis of the host and pathogen response during spaceflight infection. Rather than focusing on the direct quantitative comparison between the infected flight and ground cultures, we instead focused on evaluating the gene expression patterns elicited during either a spaceflight or ground-based infection relative to their respective controls (infected vs. uninfected for flight and ground) (Fig. 2; Supplementary Tables 4–7). However, we also include the direct comparisons of the host response to infection between flight and ground for comparison in future studies (Supplementary Table 8), as well as the bacterial genes expressed above background during the flight and ground infections (Supplementary Tables 9 and 10) and directly between flight and ground infected cultures (Supplementary Table 11).

**Fig. 2:**
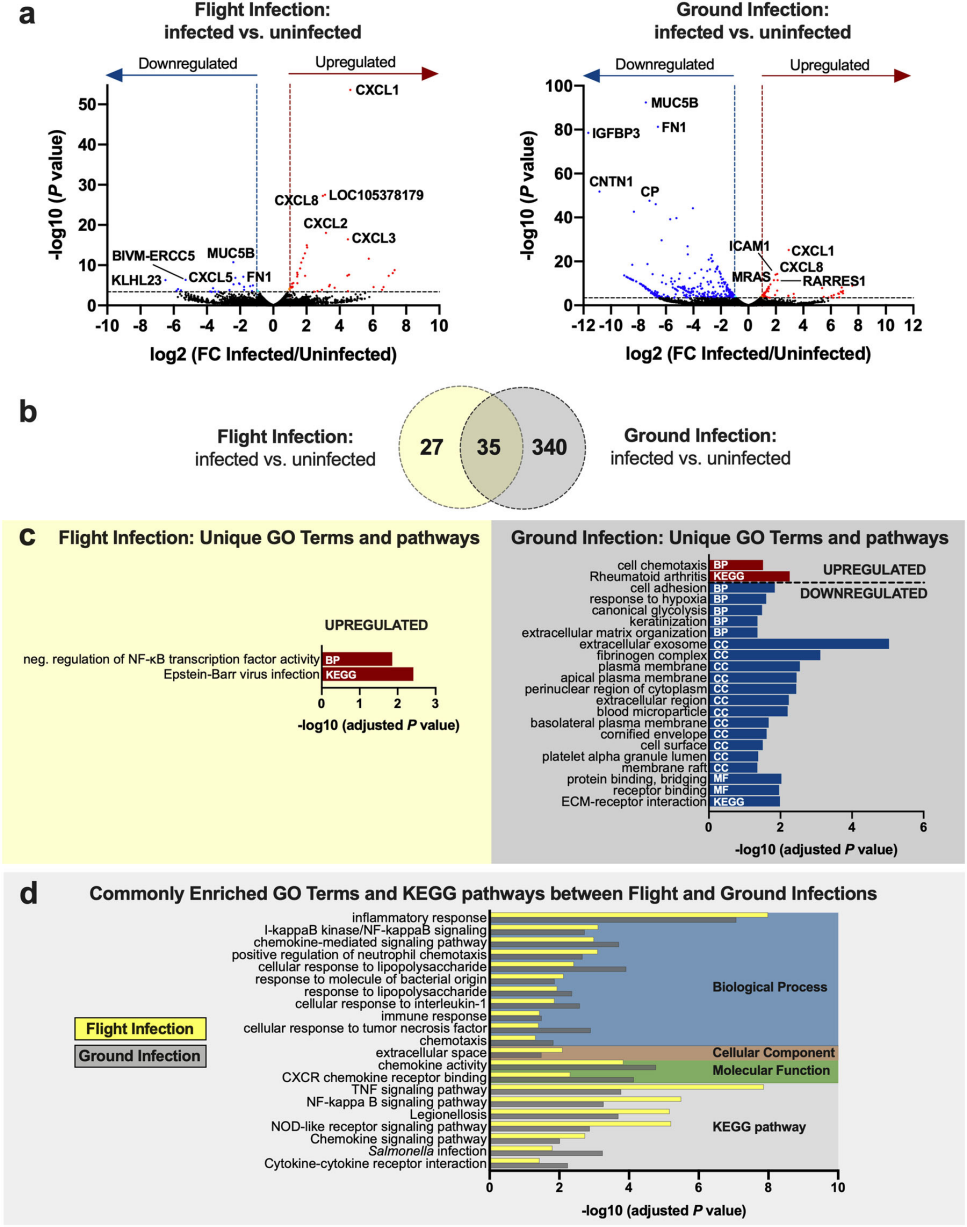
Transcriptional responses of HT-29 cells elicited in response to infection with *S*. Typhimurium during flight or ground culture. **a** Volcano plots. Differentially expressed genes between infected and uninfected host cells during spaceflight (left panel) or in matching ground controls (right panel). Red: significantly upregulated genes in response to infection. Blue: significantly downregulated genes. Significance determined according to a log_2_ fold change in expression of at least ±1 and FDR < 0.05. The top 5 significantly up and downregulated genes are labeled. **b** Venn diagram. Overlapping and distinct genes elicited in response to infection in flight and ground cultures. **c**, **d** Uniquely and commonly enriched GO terms and KEGG pathways in response to infection either in flight or ground cultures relative to their respective uninfected controls. Analysis was performed in DAVID 6.8 using upregulated (red) or downregulated (blue) transcripts using an EASE score of 0.05 and Benjamini–Hochberg correction to determine significantly enriched terms and pathways (adjusted *P* < 0.05). BP biological process, CC cellular component, MF molecular function. Bars in **d** indicate the results for a flight infection (yellow; flight infected/uninfected) and ground infection (gray; ground infected/uninfected).

Analysis of flight and ground cultures indicates both common and distinct host transcriptional profiles in response to *Salmonella* infection (Fig. 2; Supplementary Tables 4–7). Overall, 35 transcripts were altered in response to infection in both flight and ground conditions; 28 genes in the same direction and 7 oppositely regulated (Fig. 2b, Supplementary Tables 4–7). Relative to their respective uninfected controls, both flight- and ground-infected cultures displayed enrichment in biological functions and pathways expected for *Salmonella*-infected cultures, including the upregulation of genes involved in inflammation (TNF, IL-1, NF-κB, and NOD signaling), response to lipopolysaccharide (LPS), and cell chemotaxis (Fig. 2d; Supplementary Tables 5 and 7). Interaction of *Salmonella* with the intestinal epithelium initiates a series of signaling cascades leading to intestinal inflammation^58^. In vivo, this defensive response serves to recruit neutrophils and other immune cells to the site of infection but can also lead to the generation of ROS that confers a strategic advantage for *Salmonella* to outcompete resident microbiota^58^. Both flight and ground infections induced upregulation of *IRAK2*, a protein that plays a central role in TLR signaling to NF-κB for the production of pro-inflammatory mediators^59^. NF-κB-related genes commonly induced during both flight and ground infections included the Rel protein *NFKB2* and the atypical IκB member, *NFKBIZ* (Supplementary Tables 4 and 6). *RELB* and *REL*, which encode distinct Rel proteins which are induced in response to pathogen-associated molecular patterns/PAMPs (e.g., flagellin, LPS), were also upregulated in flight and ground infections, respectively (Supplementary Tables 4 and 6). In line with these observations, both flight and ground infections led to the upregulation of transcripts encoding several chemokines important for recruiting neutrophils and other immune cells to the site of infection, including *CXCL1, CXCL2, CXCL3*, *CXCL8*, and *CCL20*. *NFKBIZ* encodes NF-κB inhibitor zeta (IκBζ), which both represses and activates select NF-κB genes^60^. IκBζ is rapidly induced in response to infection or exposure to potent TLR stimuli and is an important inhibitor of nuclear NF-κB activity to prevent uncontrolled inflammation^61^. These findings confirm that during spaceflight, *Salmonella* can induce a subset of the known biosignatures of infection in the host at the transcriptional level.

In addition to those commonly observed responses, both flight and ground cultures also displayed unique transcriptional responses to infection (Fig. 2b, c; Supplementary Tables 4–7). DAVID analysis of upregulated genes in the infected flight cultures indicated that they were enriched for negative regulation of NF-κB transcription factor activity as well as those involved in Epstein–Barr virus infection (Fig. 2c). Genes belonging to these categories overlap with those known to be important for *Salmonella* infection. Two uniquely upregulated genes of interest belonging to these categories were anti-inflammatory mediator *NFKBIA* (IκBα) and NF-κB transcription factor, *RELB*. IκBα functions by sequestering NF-κB in the cytoplasm to prevent its translocation to the nucleus^62^. RelB is induced in response to severe systemic inflammation, LPS, flagellin and/or TNF stimulation and is required for sustained expression of IκBα during LPS tolerance^62^. Both proteins are known to be targeted by *Salmonella* to suppress host inflammation and/or apoptosis^63,64^. Changes in the expression of these transcripts may reflect an attempt by the host and/or pathogen to initiate processes to suppress excessive inflammation or apoptosis during the flight infection.

Ground cultures uniquely displayed enrichment in select upregulated genes associated with cell chemotaxis and inflammation (the latter include genes classified by DAVID under the ‘Rheumatoid arthritis’ category but overlap in their role for *Salmonella* infection). Conversely, downregulated genes were enriched for diverse biological processes (e.g., cell adhesion, ECM organization, fibrinogen complex, glycolysis, hypoxia), cellular components (e.g., cell surface/membrane, exosome, and fibrinogen complex), molecular functions associated with binding and KEGG pathway involved in ECM-receptor interaction. Interestingly, although we observed the expected stimulation of *TLR4* (Toll-like receptor 4) in the ground-based infection (Supplementary Table 6), this did not occur in the flight cultures at the time point evaluated. In alignment with the TLR4 trends, LPS-inducible *PIGR* was also upregulated only during the ground infection (Supplementary Table 6). A previous study using astronaut monocytes observed a significant decrease in TLR4 expression immediately following return from a space shuttle mission relative to measurements taken 10 days before launch and 15 days post landing^65^. Similarly, *TLR4* was also downregulated in blood cells in-flight and post-flight relative to pre-flight levels during the Twins study^48^. *PIGR* encodes polymeric immunoglobulin receptor which transports dimeric secretory IgA (sIgA) across the epithelium and into intestinal secretions as part of the first line of defense in mucosal immunity. Our findings could indicate the potential for an altered intestinal mucosal immune response to infection in spaceflight. Hindlimb unloading disrupted intestinal immune homeostasis in rats, leading to decreased sIgA levels in the ileum, although the activation of TLR4/MyD88/NF-kB was still observed^66^. In astronauts, *PIGR* and/or sIgA expression in saliva or blood has increased following spaceflight^48,67,68^. However, sIgA levels in extraintestinal sites are not always predictive of intestinal responses^69,70^. Thus, there is a need for further investigation into how spaceflight regulates these intestinal-specific responses.

iTRAQ analysis further revealed unique differences at the protein level in HT-29 cells for flight and ground infections. Keratins 8 and 18 (KRT 8, KRT18), both targets of the *Salmonella* effector SipC during invasion^56^, were downregulated in the infected flight cultures relative to the uninfected flight cultures (Supplementary Table 3). In contrast, these proteins were upregulated in response to infection in ground cultures (Supplementary Table 3). Infected flight cultures also displayed the downregulation of two additional cytoskeletal proteins, ACTG1 and TUBA1C, as well as a retinal dehydrogenase, ALDH1A1. As mentioned above, spaceflight-induced alterations in the uninfected flight cultures in several of these structural proteins relative to the uninfected ground controls, which may indicate the presence of an altered or dysregulated cytoskeletal landscape at the starting point for the infection. Spaceflight is known to alter the organization and/or expression of structural proteins^14,20,21^. Infection in ground controls induced changes in the expression of proteins with various functions, including heat shock/protein folding, RNA-binding, calcium-sensing, and disulfide bond catalysis (Supplementary Table 3). Heat shock protein HSPA1A was upregulated during infection in ground cultures but not in response to infection in flight. This difference between the flight and ground infections could possibly be attributed to the fact that the protein was already 2.4-fold upregulated in flight even in the absence of infection (see uninfected flight vs. ground comparison in Supplementary Table 3). Thus, the flight-cultured cells may have had sufficient amounts of HSPA1A protein readily available for response to infection. HSPA1A is a major stress-inducible member of the HSP70 family and can be upregulated as part of a cytoprotective response to a variety of stressors, including infection^71^. Heat shock proteins have previously shown altered expression (RNA and protein) in a variety of mammalian, plant, and prokaryotic cells during spaceflight or spaceflight analog culture, though not in a unified direction^72^, which is not surprising given their diverse functions. For example, multiple HSP70 transcripts were upregulated during BRIC-16, an *Arabidopsis thaliana* tissue culture payload aboard STS-131 (the same Shuttle mission as the present study)^72^.

We also performed a direct comparison between infected flight and ground cultures under the previously described limitations (Supplementary Table 8). Direct comparison indicated that infection during spaceflight-induced upregulation of host genes associated with inflammation, response to *Salmonella* challenge and/or wound healing relative to infected ground controls, including *CXCL8* (IL-8), *CXCL2*, *CSF2*, *IL36B, MAPK13*, *JUN*, *FOS, KLC2, NCF2, FLNA, RHOG*, among others (Supplementary Table 8). IL-8 chemokine expression can be stimulated by both LPS and flagellin^73^. Elevated IL-8 levels were previously observed in a postflight challenge using purified LPS from *Escherichia coli* (*E. coli*) and astronaut immune cells, the latter collected either in-flight or at landing (relative to pre-flight levels)^4,65^. Interestingly, both increased and decreased IL-8 responses have been observed following postflight LPS challenge in crew immune cells^74^. An overall blunted response to *E. coli* LPS was observed when endothelial cells were challenged during spaceflight, including only a modest increase in IL-8 expression by flight cultures relative to ground^75^. As is the case in terrestrial studies, IL-8 responses to LPS challenge in the context of spaceflight will depend on the type of LPS and host cells evaluated, as well as LPS exposure in the context of a live pathogen. In this study, *TLR4* was significantly downregulated in the infected flight samples relative to infected ground cultures (Supplementary Table 8), which suggests that the enhanced expression of pro-inflammatory mediators (e.g., *CXCL8*) in the infected flight cultures may rely more heavily on other interactions, such as flagellin-mediated interactions with TLR5. Alternatively, the kinetics of infection may have been altered between flight and ground cultures, where *TLR4* expression in the infected flight cultures may have occurred outside of our analysis time point. Future studies following the in-flight kinetics of infection would provide insight in this regard. iTRAQ analysis indicated that infection during spaceflight induced downregulation of multiple cytoskeletal genes and retinal dehydrogenase 1 relative to infected ground cultures (Supplementary Table 3).

Direct comparison of the bacterial transcripts between infected flight and ground cultures indicated 328 upregulated genes and 1 downregulated gene (Supplementary Table 11). Transcripts associated with metabolism (glycolysis/gluconeogenesis, TCA cycle, purine and pyrimidine metabolism, amino sugar and nucleotide sugar metabolism), protein translation, antibiotic synthesis, amino acid biosynthesis, RNA degradation, and biosynthesis of siderophore group non-ribosomal peptides were upregulated in flight. We also observed the upregulation of the AcrAB-TolC multidrug efflux pump (Supplementary Table 11). Though we did not see changes in *Salmonella* pathogenicity island 1 or 2 (SPI-1 or SPI-2) virulence genes, transcripts encoding major global virulence and/or stress regulators *rpoS*, *rpoE*, and *rpoH* were upregulated in flight relative to ground controls.

Another global regular, *hfq*, was found to be expressed above background levels in the flight cultures, but not the ground cultures (Supplementary Tables 9 and 10). However, in the direct comparison between infected flight and ground cultures, while we did observe an upward trend in *hfq* expression during spaceflight, it was not significant by FDR (7.8-fold; FDR = 0.24, *p* value = 0.006). In our previous studies, *hfq* was downregulated in *Salmonella* in response to both spaceflight and spaceflight analog culture^9,12^. Deletion of the gene impacted RWV trends in acid stress resistance and macrophage survival^9^. Hfq post-transcriptionally regulates gene expression by pairing mRNAs with cognate small non-coding RNAs to either inhibit or facilitate translation^76^. Given the diverse functions of *hfq* and its ability to both positively and negatively regulate translation, its absolute expression level can change depending on environmental conditions^77^. The conditions in our current study (GTSF-2 media, 37C, 5% CO_2_) are very different from those in our STS-115 experiment which solely looked at pure cultures of *Salmonella* (Lennox Broth/LB, ambient temperature). In addition, our STS-115 study was performed in the absence of the host, as all infections with microgravity-grown bacteria were done immediately upon landing. In our present study, the bacteria were co-cultured with host cells that had either been exposed to spaceflight prior to infection or infected as ground controls. Thus, the host cells in our current study experienced different environmental conditions (microgravity vs. unit gravity), which could also potentially contribute to observed bacterial differences. In addition, the cellular mRNA and protein concentration of *hfq* is tightly controlled by autoregulation and mRNA expression is also under control of several promoters, including σ32 (encoded by *rpoH*, a global stress response regulator that was also upregulated in response to spaceflight, Supplementary Table 11)^77^. It is unlikely that the Hfq protein was completely suppressed in ground cultures given its important role in diverse cellular functions; however, since bacterial proteins were not detected in our proteomics analysis these levels are unknown. If indeed elevated amounts of Hfq protein were present in the ground cultures relative to flight, it is possible that mRNA expression was inhibited in the ground cultures via autoregulation. Future studies will seek to confirm these gene expression trends and identify the associated regulatory mechanisms.

To our knowledge, this study was the first to examine how human cells respond to microbial infection when both the host and pathogen are simultaneously exposed to spaceflight. In addition, it is the first global transcriptomic and proteomic profiling of human intestinal epithelial cultures during spaceflight (either infected or uninfected). Although the sample size was small in this study due to technical limitations, the results from this investigation suggest that spaceflight altered the transcriptional and proteomic profiles of both uninfected and infected host cells. Spaceflight culture of the uninfected cells led to the upregulation of genes associated with IFN-I signaling and decreased expression of multiple bioenergetic pathways, suggesting the potential for decreased proliferation relative to ground cultures. Decreased proliferation could be due to the changes in tumorigenic potential, differentiation or DNA damage responses. *Salmonella* infection induced a core set of pro-inflammatory responses in both conditions, but spaceflight culture also induced unique transcriptional and proteomic responses not observed in the identical ground controls. Trends observed between infected flight and ground samples were consistent with a heightened response of host cells to infection with *Salmonella* during spaceflight, including amplified induction of genes encoding pro-inflammatory mediators and wound healing. These findings are important to consider in the context of previously observed changes in astronaut intestinal microbiota^3,48,78^, where some alterations were linked with decreased abundance of anti-inflammatory species and increases in select pro-inflammatory species^3,78^. In addition, the use of nutritional countermeasures to prevent or mitigate infection including the modulation of nutritional supplements^2,10^ or probiotic microbes for intestinal health during spaceflight (including for protection against pathogens)^79–81^ are active areas of research and have shown promise in ground-based studies. Our investigation adds to the growing number of studies aimed at mitigating infectious disease risks to humans during spaceflight missions and for the general public on Earth.

## Methods

### Mammalian cell lines, bacterial strains, and flight hardware

The human colonic epithelial cell line, HT-29 (ATCC, HTB-38), was cultured at 37 °C and 5% CO_2_ in GTSF-2 media (Hyclone) supplemented with 10% heat-inactivated FBS (ThermoFisher Scientific), 2.25 g sodium bicarbonate (Fisher Scientific) and 5 mL insulin transferrin selenite (Sigma-Aldrich) per liter. Media used to expand cells as monolayers contained Penicillin–Streptomycin (10 units/mL and 10 µg/mL, respectively; Sigma-Aldrich) and fungizone (0.5 µg/mL). Flight culture media contained only streptomycin (Sm) and fungizone. Infection studies were performed with wild-type *S*. Typhimurium χ3339, a mouse-passaged derivative of SL1344 which is naturally Sm-resistant^24^. Bacteria were cultured in Lennox Broth (LB) containing 0.3 M NaCl prior to loading in the hardware, as described below. HT-29 cells were cultured in 2.2 mL hollow fiber bioreactors (Spectrum Labs) that were housed in the Cell Culture Module (CCM; Tissue Genesis). The CCM hardware enabled automated media perfusion, addition of bacteria to perform infections and sample fixation. Flight comparisons were made to identical ground controls housed in the same hardware at NASA Kennedy Space Center (KSC). The flight hardware and experimental layout are shown in Fig. 1.

### Preparation of *S*. Typhimurium

Ten days before launch, *S*. Typhimurium strain χ3339 was streaked from a glycerol stock onto LB agar plates containing 100 µg/mL Sm and incubated at 37 °C overnight. Bacterial colonies were then used to inoculate overnight cultures grown for 15–16 h. with aeration in LB media containing 0.3 M NaCl and 100 µg/mL Sm at 37 °C. Cultures were pelleted at 7000 r.p.m. and resuspended in Dulbecco’s Phosphate Buffered Saline (DPBS; Invitrogen). Bacteria were quantified by plating for viable CFU per mL and diluted to 1 × 10^7^ CFU/mL in DPBS to maintain the bacteria as viable but not actively growing. On L-7, flight bacteria bags were filled with 2 mL of the cultures and maintained at room temperature until attachment to the flow paths 2 days prior to launch (L-2).

### Preparation of hollow fiber bioreactors, media bags and fixatives

Sterile bioreactors were wetted with 70% ethanol (10 mL) and then rinsed in triplicate with 10 mL DPBS. A 1 mg/mL collagen I solution (bovine, Corning) in DPBS was then introduced through the ECS ports to coat the hollow fibers. After 1 h, bioreactors were rinsed in triplicate with DPBS through the ECS ports to remove unbound collagen. Bioreactors were rinsed in triplicate with 10 mL of warm flight medium and placed at 37 °C until ready for loading with HT-29 cells. Flight and ground control bags contained either 60 mL of flight culture media or 40 mL RNAlater II fixative (Ambion) for transcriptomic and proteomic analyses.

### Preparation and loading of HT-29 cells into flight hardware

HT-29 cells were expanded as monolayers in T-75 flasks. Four days prior to launch, confluent monolayers were washed in warm Hank’s Balanced Salt Solution without calcium and magnesium (ThermoFisher Scientific), dissociated using 0.25% Trypsin-EDTA (Corning), and counted using trypan blue exclusion to determine cell viability. Approximately 1 × 10^6^ HT-29 cells were loaded into twelve 2.2 mL hollow fiber bioreactors (Spectrum) through the ECS ports (Fig. 1). Six bioreactors were designated for flight (three infected and three time-matched uninfected controls) and six for identical ground controls. Bioreactors were placed at 37 °C and rotated every 15 min until attachment to the CCM rails together with the flight media bags. CCM rails are organizational subunits within the CCM that facilitate gas and media delivery. Rails were placed into a 37 °C incubator and monitored for contamination. Media was circulated within the ICS of the fibers during the experiment at a flow rate of 6 mL/min. The day prior to launch, the fully loaded rails containing the HT-29 cells, media, fixatives and bacteria (maintained in stasis in DPBS) were integrated into the CCM, which was then sealed, powered and the temperature set to 37 °C. The CCM was turned over to the KSC integration team at L-18 h. STL-IMMUNE launched aboard Space Shuttle Discovery (STS-131) on April 5, 2010 at 6:21 a.m.

### In-flight operations

At approximately L + 3 h, astronauts activated the on-orbit programming of the CCM. On mission elapsed day 11, the CCM was programmed to automatically inject 1 mL (~1 × 10^7^ bacteria) into a subset of the hollow fiber bioreactors to enable infection at the apical surface of host cells. Six hours after the bacteria were injected, RNAlater II was injected into the designated bioreactors and the rails were turned off to maintain sample temperature at 29–30 °C until landing.

### Postlanding processing

STL-IMMUNE landed at KSC at 9:08 a.m. on April 20, 2010. All bioreactors and bags were removed from the rails and stored at 4 °C. Each reactor was carefully opened, cells and hollow fibers were maintained in RNAlater II at −80 °C until further processing. Inspection of the remaining volumes in the bacterial bags post flight indicated that all infected flight cultures received the intended 1 mL bacterial injection. Infected ground cultures received 0.8 and 0.4 mL injections for two replicates used for both the RNA-seq/iTRAQ analysis, and the third replicate received a 1.2 mL injection (this sample used for iTRAQ analysis only). For processing, RNAlater II-fixed samples were thawed at 4 °C. Samples were concentrated using a Vivaspin 20 (0.2 µm) concentrator. Cells were allowed to settle at 4 °C, the supernatant was removed and then processed for iTRAQ and/or RNA-seq analysis.

### Isobaric tags for relative and absolute quantitation (iTRAQ) analysis

Proteomic analysis using iTRAQ was performed on total cellular protein from HT-29 cells from triplicate flight and ground bioreactors (*n* = 3 for flight and ground infected and uninfected cultures). Cells were lysed with 1 cell bed volume of RLT buffer (Qiagen) supplemented with cOmplete EDTA-free protease inhibitor (Roche), vortexed and passed 5–7 times through a 25G needle to facilitate lysis. After a 10-min incubation on ice, lysates were centrifuged at 12,000 rpm for 10 min at 4 °C. Lysates were added to a desalting column (Pierce) and eluted by centrifugation at 1000 × *g* for 2 min. From each condition 25 μg protein sample was prepared according to the manufacturer’s instruction (iTRAQ reagents—4plex Application kit, Applied Biosystems). Briefly, after protein reduction, alkylation, tryptic digestion (Promega), and labeling with iTRAQ 4plex, peptide samples were quenched before pooled and vacuum-dried. The combined iTRAQ-reagent labeled sample was resuspended in 2% acetonitrile and 0.1% trifluoroacetic acid before separated with a 145-min gradient elution (using a Tempo LC MALDI Spotting system (Applied Biosystems)) and analyzed with a 4800 MALDI-tandem time-of-flight (TOF/TOF) mass spectrometer (Applied Biosystems) as described^82^. Protein identification and quantification was performed with ProteinPilot 4.0 software using Paragon algorithm. The search parameters were as follows: iTRAQ 4plex (peptide labeled), trypsin as the digestion agent, UniProt database *Homo sapiens* (created on March 2009), ID focus: biological modification. Proteins were considered as differentially expressed according to the following criteria: greater than a twofold expression change, *p* value ≤ 0.05 (two-tailed ANOVA). Differential proteins were identified with at least three unique peptides (>95% confidence, 1% FDR). Identified and quantified proteins by LC MALDI-MS/MS are shown in Supplementary Dataset 1.

### RNA-Seq

RNA was extracted from flight and ground cultures using remaining samples following the proteomics processing. From the remaining samples, RNA-seq analyses were performed using duplicate flight and ground bioreactors for all conditions (*n* = 2 biological replicates) except for the infected flight samples, which only had sufficient sample remaining to evaluate duplicate technical aliquots from a single bioreactor (i.e., *n* = 1 biological; *n* = 2 technical replicates). RNAlater II-fixed samples were lysed with 700 µl Qiazol (Qiagen), vortexed extensively and passed several times through a 25G needle. After a 5-min static incubation at room temperature with intermittent vortexing, samples were added to MaXtract High-Density tubes (Qiagen) together with 100 µl RNase-free water (Qiagen) and 200 µl chloroform (Sigma-Aldrich). Samples were then processed using the miRNeasy kit (Qiagen) according to the manufacturer’s instructions, including on-column DNase-treatment. RNA was quantified using a Nanodrop spectrophotometer and the integrity validated using an Agilent Bioanalyzer 2100 to confirm an RNA integrity number (RIN) of 7 or greater for all samples. All RIN were between 7 and 8 except for one of the uninfected ground control samples which had a RIN of 6.4.

RNA was fragmented with heat and magnesium to roughly 300 bp using the KAPA HyperPrep RNA-seq kit (Roche). The KAPA Hyper RNA-seq kit and Illumina-compatible adapters (IDT) were used for the remaining library construction. Adapter ligated molecules were cleaned using Kapa pure beads (Kapa Biosciences) and amplified with Kapa HIFI enzyme. Each library was then analyzed for fragment size on an Agilent Tapestation and quantified by qPCR (KAPA Library Quantification Kit) on Quantstudio 5 (ThermoFisher Scientific). Libraries were then multiplexed and sequenced on 2 × 150 flow cells on the NovaSeq platform (Illumina) at University of Colorado Anschutz Medical Campus Genomics and Microarray Core. RNA-seq reads for each sample were quality checked using FastQC v0.10.1 and aligned to Human GRCh38.p7 primary assembly and S. Typhimurium LT2 genome using STAR v2.5.1b. A series of quality control metrics were generated on the STAR outputs. Cufflinks v2.2.1 was used to report FPKM values (Fragments Per Kilobase of transcript per Million mapped reads) and read counts. Transcripts per million was calculated by an in-house R script. Differential expression analysis was performed with EdgeR package from Bioconductor v3.2 in R 3.2.3. Multidimensional scaling plots were drawn by plotMDS, in which distances correspond to leading log-fold-changes between samples. For each pairwise comparison, genes with FDR <0.05 were considered significant and log_2_-fold changes of expression between conditions (logFC) were reported.

### GO biological process and KEGG pathway enrichment analysis

DAVID 6.8 was used to perform the enrichment analysis of RNA-seq data. Data were corrected using the Benjamini–Hochberg procedure, had threshold count of 2 and EASE score of 0.05.

### Reporting summary

Further information on experimental design is available in the Nature Research Reporting Summary linked to this paper.

## Supplementary information

Supplementary Tables 1 to 11

Supplementary Dataset 1

Reporting Summary Checklist

## Data Availability

All data generated during this study are either included in the manuscript and its Supplementary files (Supplementary Dataset 1) or are available from the corresponding authors upon reasonable request. RNA-Seq data are available at the Gene Expression Omnibus (GEO) database (GSE156066; BioProject PRJNA656571) and NASA GeneLab (https://genelab-data.ndc.nasa.gov/genelab/accession/GLDS-323).

## References

[1] Mcpee, J. C. & Charles, J. B. *Human Health and Performance Risks of Space Exploration Missions*. (US National Aeronautics and Space Admin; NASA SP-2009-3405 edition, 2010).

[2] Crucian BE (2018). Immune system dysregulation during spaceflight: potential countermeasures for deep space exploration missions. Front Immunol..

[3] Voorhies, A. A. & Lorenzi, H. A. The challenge of maintaining a healthy microbiome during long-duration space missions. *Front. Astron. Space Sci.* **3**, 10.3389/fspas.2016.00023 (2016).

[4] Crucian B (2015). Alterations in adaptive immunity persist during long-duration spaceflight. NPJ Microgravity.

[5] Castro VA, Thrasher AN, Healy M, Ott CM, Pierson DL (2004). Microbial characterization during the early habitation of the International Space Station. Micro. Ecol..

[6] Yamaguchi N (2014). Microbial monitoring of crewed habitats in space-current status and future perspectives. Microbes Environ..

[7] Kish, A. L. et al. *Biostability and Microbiological Analysis Of Shuttle Crew Refuse*. (SAE Technical Paper #2002-01-2356, 2002).

[8] Singh NK, Wood JM, Karouia F, Venkateswaran K (2018). Succession and persistence of microbial communities and antimicrobial resistance genes associated with International Space Station environmental surfaces. Microbiome.

[9] Wilson JW (2007). Space flight alters bacterial gene expression and virulence and reveals a role for global regulator Hfq. Proc. Natl Acad. Sci. USA.

[10] Wilson JW (2008). Media ion composition controls regulatory and virulence response of *Salmonella* in spaceflight. PLoS ONE.

[11] Wilson JW (2002). Low-Shear modeled microgravity alters the *Salmonella enterica* serovar typhimurium stress response in an RpoS-independent manner. Appl. Environ. Microbiol..

[12] Wilson JW (2002). Microarray analysis identifies *Salmonella* genes belonging to the low-shear modeled microgravity regulon. Proc. Natl Acad. Sci. USA.

[13] Gilbert, R. et al. Spaceflight and simulated microgravity conditions increase virulence of *Serratia marcescens* in the *Drosophila melanogaster* infection model. *npj Microgravity* **6**, 4 (2020). 10.1038/s41526-019-0091-2 PMC7000411 32047838

[14] Nickerson, C. A, Pellis, N. R. & Ott, C. M. *Effect of Spaceflight and Spaceflight Analogue Culture on Human and Microbial Cells: Novel Insights into Disease Mechanisms* (Springer, 2016).

[15] Horneck, G., Klaus, D. M. & Mancinelli, R. L. Space microbiology. *Microbiol. Mol. Biol. Rev.* **74**, 121–156, (2010). 10.1128/MMBR.00016-09 PMC2832349 20197502

[16] Mermel LA (2013). Infection prevention and control during prolonged human space travel. Clin. Infect. Dis..

[17] Barrila, J. et al. Modeling host-pathogen interactions in the context of the microenvironment: three-dimensional cell culture comes of age. *Infect. Immun.* **86**, 10.1128/IAI.00282-18 (2018). 10.1128/IAI.00282-18 PMC6204695 30181350

[18] Bissell MJ, Aggeler J (1987). Dynamic reciprocity: how do extracellular matrix and hormones direct gene expression?. Prog. Clin. Biol. Res.

[19] Ingber DE (1999). How cells (might) sense microgravity. FASEB J..

[20] Hughes-Fulford M (2003). Function of the cytoskeleton in gravisensing during spaceflight. Adv. Space Res..

[21] Lewis ML (2002). The cytoskeleton, apoptosis, and gene expression in T lymphocytes and other mammalian cells exposed to altered gravity. Adv. Space Biol. Med..

[22] Ibarra JA, Steele-Mortimer O (2009). Salmonella-the ultimate insider. Salmonella virulence factors that modulate intracellular survival. Cell Microbiol..

[23] Gruenheid S, Finlay BB (2003). Microbial pathogenesis and cytoskeletal function. Nature.

[24] Nickerson CA (2000). Microgravity as a novel environmental signal affecting Salmonella enterica Serovar typhimurium virulence. Infect. Immun..

[25] Castro SL, Nelman-Gonzalez M, Nickerson CA, Ott CM (2011). Induction of attachment-independent biofilm formation and repression of Hfq expression by low-fluid-shear culture of Staphylococcus aureus. Appl. Environ. Microbiol..

[26] Crabbé A (2010). Response of *Pseudomonas aeruginosa* PAO1 to low shear modelled microgravity involves AlgU regulation. Environ. Microbiol..

[27] Crabbe A (2011). Transcriptional and proteomic responses of *Pseudomonas aeruginosa* PAO1 to spaceflight conditions involve Hfq regulation and reveal a role for oxygen. Appl. Environ. Microbiol..

[28] Grant KC, Khodadad CLM, Foster JS (2014). Role of Hfq in an animal–microbe symbiosis under simulated microgravity conditions. Int. J. Astrobiol..

[29] Larsen SB, Cowley CJ, Fuchs E (2020). Epithelial cells: liaisons of immunity. Curr. Opin. Immunol..

[30] Allaire JM (2018). The intestinal epithelium: central coordinator of mucosal immunity. Trends Immunol..

[31] Carregaro F, Stefanini AC, Henrique T, Tajara EH (2013). Study of small proline-rich proteins (SPRRs) in health and disease: a review of the literature. Arch. Dermatol. Res..

[32] Pradervand S (2004). Small proline-rich protein 1A is a gp130 pathway- and stress-inducible cardioprotective protein. EMBO J..

[33] Pyle AL (2008). Regulation of the atheroma-enriched protein, SPRR3, in vascular smooth muscle cells through cyclic strain is dependent on integrin alpha1beta1/collagen interaction. Am. J. Pathol..

[34] Pecaut, M. J. et al. Is spaceflight-induced immune dysfunction linked to systemic changes in metabolism? *PLOS ONE* **12**, e0174174 (2017). 10.1371/journal.pone.0174174 PMC5443495 28542224

[35] McNab F, Mayer-Barber K, Sher A, Wack A, O’Garra A (2015). Type I interferons in infectious disease. Nat. Rev. Immunol..

[36] Kotredes KP, Thomas B, Gamero AM (2017). The protective role of type I interferons in the gastrointestinal tract. Front. Immunol..

[37] Katlinskaya YV (2016). Type I interferons control proliferation and function of the intestinal epithelium. Mol. Cell Biol..

[38] Shi, L. et al. Spaceflight and simulated microgravity suppresses macrophage development via altered RAS/ERK/NFkappaB and metabolic pathways. *Cell. Mol. Immunol.*10.1038/s41423-019-0346-6 (2020). 10.1038/s41423-019-0346-6 PMC8167113 31900461

[39] Fritz V, Fajas L (2010). Metabolism and proliferation share common regulatory pathways in cancer cells. Oncogene.

[40] Leschner S, Weiss S (2010). Salmonella-allies in the fight against cancer. J. Mol. Med..

[41] Honer zu Bentrup K (2006). Three-dimensional organotypic models of human colonic epithelium to study the early stages of enteric salmonellosis. Microbes Infect..

[42] Barker N, Tan S, Clevers H (2013). Lgr proteins in epithelial stem cell biology. Development.

[43] Field CJ, Johnson IR, Schley PD (2002). Nutrients and their role in host resistance to infection. J. Leukoc. Biol..

[44] Steeb B (2013). Parallel exploitation of diverse host nutrients enhances Salmonella virulence. PLoS Pathog..

[45] Jessup JM (2000). Microgravity culture reduces apoptosis and increases the differentiation of a human colorectal carcinoma cell line. Vitr. Cell Dev. Biol. Anim..

[46] Kumar S, Suman S, Fornace AJ, Datta K (2018). Space radiation triggers persistent stress response, increases senescent signaling, and decreases cell migration in mouse intestine. Proc. Natl Acad. Sci. USA.

[47] Moreno-Villanueva M, Wong M, Lu T, Zhang Y, Wu H (2017). Interplay of space radiation and microgravity in DNA damage and DNA damage response. NPJ Microgravity.

[48] Garrett-Bakelman, F. E. et al. The NASA Twins Study: a multidimensional analysis of a year-long human spaceflight. *Science* **364**. 10.1126/science.aau8650 (2019). 10.1126/science.aau8650 PMC7580864 30975860

[49] George K, Rhone J, Beitman A, Cucinotta FA (2013). Cytogenetic damage in the blood lymphocytes of astronauts: effects of repeat long-duration space missions. Mutat. Res.

[50] Barrila J (2016). Spaceflight modulates gene expression in the whole blood of astronauts. npj Microgravity.

[51] Simpson-Haidaris PJ (1998). Induction of fibrinogen expression in the lung epithelium during Pneumocystis carinii Pneumonia. Infect. Immun..

[52] Molmenti EP, Ziambaras T, Perlmutter DH (1993). Evidence for an acute phase response in human intestinal epithelial cells. J. Biol. Chem..

[53] Andoh A (1998). Detection of complement C3 and factor B gene expression in normal colorectal mucosa, adenomas and carcinomas. Clin. Exp. Immunol..

[54] Kulkarni HS, Liszewski MK, Brody SL, Atkinson JP (2018). The complement system in the airway epithelium: an overlooked host defense mechanism and therapeutic target?. J. Allergy Clin. Immunol..

[55] Wei Z, Batagov AO, Carter DR, Krichevsky AM (2016). Fetal bovine serum RNA interferes with the cell culture derived extracellular RNA. Sci. Rep..

[56] Schleker S (2012). The current Salmonella-host interactome. Proteom. Clin. Appl..

[57] Maier T, Guell M, Serrano L (2009). Correlation of mRNA and protein in complex biological samples. FEBS Lett..

[58] Broz P, Ohlson MB, Monack DM (2012). Innate immune response to Salmonella typhimurium, a model enteric pathogen. Gut Microbes.

[59] Keating SE, Maloney GM, Moran EM, Bowie AG (2007). IRAK-2 participates in multiple toll-like receptor signaling pathways to NFkappaB via activation of TRAF6 ubiquitination. J. Biol. Chem..

[60] Muller A (2018). IkappaBzeta is a key transcriptional regulator of IL-36-driven psoriasis-related gene expression in keratinocytes. Proc. Natl Acad. Sci. USA.

[61] Yamazaki S, Muta T, Takeshige K (2001). A novel IkappaB protein, IkappaB-zeta, induced by proinflammatory stimuli, negatively regulates nuclear factor-kappaB in the nuclei. J. Biol. Chem..

[62] Chen X (2009). RelB sustains IkappaBalpha expression during endotoxin tolerance. Clin. Vaccin. Immunol..

[63] Le Negrate G (2008). *Salmonella* secreted factor L deubiquitinase of *Salmonella typhimurium* inhibits NF-kappaB, suppresses IkappaBalpha ubiquitination and modulates innate immune responses. J. Immunol..

[64] Sun H, Kamanova J, Lara-Tejero M, Galan JE (2016). A family of Salmonella type IIi secretion effector proteins selectively targets the NF-kappaB signaling pathway to preserve host homeostasis. PLoS Pathog..

[65] Kaur I, Simons ER, Kapadia AS, Ott CM, Pierson DL (2008). Effect of spaceflight on ability of monocytes to respond to endotoxins of gram-negative bacteria. Clin. Vaccin. Immunol..

[66] Jin M (2018). Responses of intestinal mucosal barrier functions of rats to simulated weightlessness. Front. Physiol..

[67] Mednieks, M. H. A. In *Oral Tissue Responses to Travel in Space. Beyond LEO - Human Health Issues for Deep Space Exploration* (ed Reynolds, R. J.) (IntechOpen, 2019).

[68] Spielmann G (2018). B cell homeostasis is maintained during long-duration spaceflight. J. Appl. Physiol..

[69] Aase A (2016). Salivary IgA from the sublingual compartment as a novel noninvasive proxy for intestinal immune induction. Mucosal Immunol..

[70] Externest D, Meckelein B, Schmidt MA, Frey A (2000). Correlations between antibody immune responses at different mucosal effector sites are controlled by antigen type and dosage. Infect. Immun..

[71] Ding XZ (2010). HSP-70 mitigates LPS/SKI-induced cell damage by increasing sphingosine kinase 1 (SK1). Prostaglandins Other Lipid Mediat.

[72] Zupanska AK, Denison FC, Ferl RJ, Paul AL (2013). Spaceflight engages heat shock protein and other molecular chaperone genes in tissue culture cells of Arabidopsis thaliana. Am. J. Bot..

[73] Audy J, Mathieu O, Belvis J, Tompkins TA (2012). Transcriptomic response of immune signalling pathways in intestinal epithelial cells exposed to lipopolysaccharides, Gram-negative bacteria or potentially probiotic microbes. Benef. Microbes.

[74] Crucian B, Stowe R, Quiriarte H, Pierson D, Sams C (2011). Monocyte phenotype and cytokine production profiles are dysregulated by short-duration spaceflight. Aviat. Space Environ. Med..

[75] Chakraborty N (2014). An integrated omics analysis: impact of microgravity on host response to lipopolysaccharide in vitro. BMC Genom..

[76] Vogel J, Luisi BF (2011). Hfq and its constellation of RNA. Nat. Rev. Microbiol.

[77] Morita T, Aiba H (2019). Mechanism and physiological significance of autoregulation of the Escherichia coli hfq gene. RNA.

[78] Voorhies AA (2019). Study of the impact of long-duration space missions at the International Space Station on the astronaut microbiome. Sci. Rep..

[79] Castro-Wallace S, Stahl S, Voorhies A, Lorenzi H, Douglas GL (2017). Response of Lactobacillus acidophilus ATCC 4356 to low-shear modeled microgravity. Acta Astronautica.

[80] Shao D (2017). Simulated microgravity affects some biological characteristics of Lactobacillus acidophilus. Appl. Microbiol. Biotechnol..

[81] Turroni, S. et al. Gut microbiome and space travelers’ health: state of the art and possible pro/prebiotic strategies for long-term space missions. *Front. Physiol.* **11**, 10.3389/fphys.2020.553929 (2020). 10.3389/fphys.2020.553929 PMC7505921 33013480

[82] Hansmeier N, Chao TC, Goldman LR, Witter FR, Halden RU (2012). Prioritization of biomarker targets in human umbilical cord blood: identification of proteins in infant blood serving as validated biomarkers in adults. Environ. Health Perspect..

