# Supplementary material for: Evaluating the effect of spaceflight on the host–pathogen interaction between human intestinal epithelial cells and Salmonella Typhimurium
Source: NPJ Microgravity. 2021 Mar 9;7:9. doi: 10.1038/s41526-021-00136-w (PMC7943786; doi:10.1038/s41526-021-00136-w)
Supplement: Supplementary file 1 — Supplementary Tables 1 to 11 [file 41526_2021_136_MOESM1_ESM.pdf]

Supplementary Tables 1 to 11

Evaluating the effect of spaceflight on the host-pathogen interaction between human intestinal epithelial cells and *Salmonella* Typhimurium

Jennifer Barrila, Shameema F. Sarker, Nicole Hansmeier, Shanshan Yang, Kristina Buss, Natalia Briones, Jin Park, Richard R. Davis, Rebecca J. Forsyth, C. Mark Ott, Kevin Sato, Cristine Kosnik, Anthony Yang, Cheryl Shimoda, Nicole Rayl, Diana Ly, Aaron Landenberger, Stephanie D. Wilson, Naoko Yamazaki, Jason Steel, Camila Montano, Rolf U. Halden, Tom Cannon, Sarah L. Castro-Wallace and Cheryl A. Nickerson

This document contains Supplementary Tables 1 through 11. Titles contain an active link to bring the reader to the appropriate starting page for each table.

Supplementary Table 1. Differentially expressed genes in uninfected Flight versus uninfected Ground HT-29 cultures (pp. 2-6)

Supplementary Table 2. Enrichment analysis of uninfected Flight versus uninfected Ground HT-29 cultures (p. 7)

Supplementary Table 3. Differentially expressed host proteins in STL-IMMUNE (p. 8)

Supplementary Table 4. Differentially expressed host genes in Flight infected vs uninfected cultures (pp. 9-10)

Supplementary Table 5. Enrichment analysis of Flight HT-29 cultures: infected versus uninfected (pp. 11-12)

Supplementary Table 6. Differentially expressed host genes in Ground infected vs uninfected cultures (pp. 13-19)

Supplementary Table 7. Enrichment analysis of Ground HT-29 cultures: infected versus uninfected (pp. 20-22)

Supplementary Table 8. Differentially expressed host genes in infected Flight vs infected Ground cultures (pp. 23-31)

Supplementary Table 9. Bacterial transcripts expressed in infected Flight HT-29 cultures (pp. 32-39)

Supplementary Table 10. Bacterial transcripts expressed in infected Ground HT-29 cultures (pp. 40-41)

Supplementary Table 11. Differential expression of bacterial transcripts between infected Flight and infected Ground HT-29 cultures (pp. 42-47)

Supplementary Table 1. Differentially expressed genes in uninfected Flight versus uninfected Ground HT-29 cultures[1]

Gene	Description	Fold Change	FDR
	**Upregulated genes (45)**		
KLHL23	kelch like family member 23	101.726	0.000271233
BIVM-ERCC5	BIVM-ERCC5 readthrough	85.544	6.51E-05
SLMO2-ATP5E	SLMO2-ATP5E readthrough	85.085	0.001528544
JMJD7-PLA2G4B	JMJD7-PLA2G4B readthrough	83.792	0.009568089
HSPE1-MOB4	HSPE1-MOB4 readthrough	73.870	0.019503319
UGT1A5	UDP glucuronosyltransferase family 1 member A5	66.273	0.006721232
RPL36A-HNRNPH2	RPL36A-HNRNPH2 readthrough	63.359	0.012933521
NPHP3-ACAD11	NPHP3-ACAD11 readthrough (NMD candidate)	56.357	0.02377648
IFNL1	interferon lambda 1	15.737	0.03985838
SPRR3	small proline rich protein 3	5.626	7.93E-16
SPRR1B	small proline rich protein 1B	3.672	2.85E-10
SPRR1A	small proline rich protein 1A	3.438	2.24E-05
DHRS9	dehydrogenase/reductase 9	3.215	3.40E-07
MCM10	minichromosome maintenance 10 replication initiation factor	2.957	0.022862199
ADAM19	ADAM metallopeptidase domain 19	2.742	0.015984152
TACSTD2	tumor-associated calcium signal transducer 2	2.664	3.06E-05
WFDC2	WAP four-disulfide core domain 2	2.598	4.93E-05
RSAD2	radical S-adenosyl methionine domain containing 2	2.589	0.001648149
CLSPN	claspin	2.481	0.00030744
CD3EAP	CD3e molecule associated protein	2.392	0.000522717
CCNA2	cyclin A2	2.341	0.004397226
RRM2	ribonucleotide reductase regulatory subunit M2	2.338	0.000723169
KIAA0101	KIAA0101	2.329	0.006721232
SAMHD1	SAM and HD domain containing deoxynucleoside triphosphate triphosphohydrolase 1	2.304	0.005688177
BRCA1	BRCA1, DNA repair associated	2.269	0.002190685
SH3TC2	SH3 domain and tetratricopeptide repeats 2	2.230	0.001391707
WDR62	WD repeat domain 62	2.223	0.017315084
ZNF185	zinc finger protein 185 (LIM domain)	2.222	0.00117612
ADGRF1	adhesion G protein-coupled receptor F1	2.215	0.001392283
TREML2	triggering receptor expressed on myeloid cells like 2	2.213	0.010207079
LAMC2	laminin subunit gamma 2	2.193	0.001421026
OAS3	2'-5'-oligoadenylate synthetase 3	2.191	0.005227343
LOC107985741	uncharacterized LOC107985741	2.159	0.009568089
IFI30	IFI30, lysosomal thiol reductase	2.144	0.045805938
SECTM1	secreted and transmembrane 1	2.116	0.003633792
EREG	epiregulin	2.095	0.011885583
KLK6	kallikrein related peptidase 6	2.092	0.009443983
GRHL3	grainyhead like transcription factor 3	2.091	0.02254808
MX1	MX dynamin like GTPase 1	2.054	0.008015452
OASL	2'-5'-oligoadenylate synthetase like	2.050	0.006942525
LOXL4	lysyl oxidase like 4	2.045	0.007140929
SULT2B1	sulfotransferase family 2B member 1	2.033	0.008694672
DPP4	dipeptidyl peptidase 4	2.026	0.016357368
GTF2A2	general transcription factor IIA subunit 2	2.022	0.011996017
CCAT1	colon cancer associated transcript 1 (non-protein coding)	2.010	0.013816545
	**Downregulated genes (262)**		
TSC22D3	TSC22 domain family member 3	-2.005	0.028211759
PDCD4	programmed cell death 4 (neoplastic transformation inhibitor)	-2.014	0.023699402
NOL3	nucleolar protein 3	-2.023	0.022862199
TBC1D8	TBC1 domain family member 8	-2.042	0.028246061
DUSP4	dual specificity phosphatase 4	-2.044	0.008015452
KLHDC7A	kelch domain containing 7A	-2.044	0.022754382
SREBF1	sterol regulatory element binding transcription factor 1	-2.050	0.007471623
ANO9	anoctamin 9	-2.069	0.019062096
ARSD	arylsulfatase D	-2.076	0.006153623
DENND5B	DENN domain containing 5B	-2.085	0.0058557

HK2	hexokinase 2	-2.089	0.00435589
METTL12	methyltransferase like 12	-2.096	0.0129733
TNS3	tensin 3	-2.101	0.003526686
MYRF	myelin regulatory factor	-2.110	0.005682348
CD24	CD24 molecule	-2.113	0.004397226
TSPAN12	tetraspanin 12	-2.121	0.01112855
C6orf223	chromosome 6 open reading frame 223	-2.149	0.004183485
ANKZF1	ankyrin repeat and zinc finger domain containing 1	-2.156	0.007822103
QPRT	quinolinate phosphoribosyltransferase	-2.161	0.00538341
CAMK2N1	calcium/calmodulin dependent protein kinase II inhibitor 1	-2.163	0.004351272
IGFBP4	insulin like growth factor binding protein 4	-2.169	0.003117576
AK4	adenylate kinase 4	-2.176	0.018424967
NAALADL2	N-acetylated alpha-linked acidic dipeptidase like 2	-2.196	0.007636672
EPS8L3	EPS8 like 3	-2.197	0.005679925
NYNRIN	NYN domain and retroviral integrase containing	-2.221	0.001332708
MYOM3	myomesin 3	-2.243	0.009568089
PTPRJ	protein tyrosine phosphatase, receptor type J	-2.253	0.001336869
KDM4B	lysine demethylase 4B	-2.269	0.007471623
HOXB3	homeobox B3	-2.272	0.002169876
HOXA3	homeobox A3	-2.303	0.01617281
SLC25A29	solute carrier family 25 member 29	-2.321	0.000710704
ARHGEF17	Rho guanine nucleotide exchange factor 17	-2.322	0.011298087
SNORA53	small nucleolar RNA, H/ACA box 53	-2.351	0.035909394
GAPDHP1	glyceraldehyde-3-phosphate dehydrogenase pseudogene 1	-2.392	0.002877052
BNIP3L	BCL2 interacting protein 3 like	-2.437	0.002025497
UGT1A9	UDP glucuronosyltransferase family 1 member A9	-2.538	0.012910824
FAM83E	family with sequence similarity 83 member E	-2.551	6.82E-05
TPM2	tropomyosin 2 (beta)	-2.552	0.004907446
TCN2	transcobalamin 2	-2.556	0.001392283
RIN2	Ras and Rab interactor 2	-2.558	0.005510304
FREM2	FRAS1 related extracellular matrix protein 2	-2.596	0.001648149
AGFG2	ArfGAP with FG repeats	-2.615	0.00221063
TSPAN9	tetraspanin 9	-2.623	0.002636918
HNF4A	hepatocyte nuclear factor 4 alpha	-2.648	0.02414016
SLC40A1	solute carrier family 40 member 1	-2.660	1.55E-05
SEMA4G	semaphorin 4G	-2.723	6.02E-06
MYADM	myeloid associated differentiation marker	-2.745	0.008045139
PDK1	pyruvate dehydrogenase kinase 1	-2.767	2.79E-05
BTD	biotinidase	-2.775	0.044183093
SH3PXD2A	SH3 and PX domains 2A	-2.787	0.009043447
PDE4D	phosphodiesterase 4D	-2.811	0.000116127
ECHDC2	enoyl-CoA hydratase domain containing 2	-2.819	0.000908827
CEACAM6	carcinoembryonic antigen related cell adhesion molecule 6	-2.866	1.15E-06
HGD	homogentisate 1,2-dioxygenase	-2.894	0.026446937
LOC107987206	uncharacterized LOC107987206	-2.902	2.05E-06
DPYSL2	dihydropyrimidinase like 2	-2.913	8.08E-05
PFKFB3	6-phosphofructo-2-kinase/fructose-2,6-biphosphatase 3	-2.917	3.40E-07
ENO2	enolase 2	-2.931	1.44E-06
ABCG1	ATP binding cassette subfamily G member 1	-2.938	0.005560988
AGR2	anterior gradient 2, protein disulphide isomerase family member	-2.955	4.22E-07
F2R	coagulation factor II thrombin receptor	-2.970	0.007471623
ANO1	anoctamin 1	-3.020	0.002874584
CLEC3A	C-type lectin domain family 3 member A	-3.060	1.13E-06
EHHADH	enoyl-CoA hydratase and 3-hydroxyacyl CoA dehydrogenase	-3.120	0.038336674
PRKCA	protein kinase C alpha	-3.194	3.88E-08
VIL1	villin 1	-3.213	8.36E-08
USH1C	USH1 protein network component harmonin	-3.230	2.63E-08
PROM1	prominin 1	-3.270	2.19E-05
MUC13	mucin 13, cell surface associated	-3.302	2.17E-08
DMTN	dematin actin binding protein	-3.329	0.00088236
TSPAN8	tetraspanin 8	-3.386	3.69E-06
CA12	carbonic anhydrase 12	-3.414	2.88E-09
ROR1	receptor tyrosine kinase like orphan receptor 1	-3.416	0.000387615
PTPRO	protein tyrosine phosphatase, receptor type O	-3.421	0.005286392
F5	coagulation factor V	-3.454	1.70E-08
CFTR	cystic fibrosis transmembrane conductance regulator	-3.459	3.26E-08

CLMN	calmin	-3.467	8.65E-08
IGF2	insulin like growth factor 2	-3.495	0.001551811
ALDH3A1	aldehyde dehydrogenase 3 family member A1	-3.517	2.69E-09
PHGR1	proline, histidine and glycine rich 1	-3.545	5.40E-05
FAM13A	family with sequence similarity 13 member A	-3.548	1.13E-06
RBPMS	RNA binding protein with multiple splicing	-3.550	0.004986765
GDA	guanine deaminase	-3.560	0.007119424
FADS1	fatty acid desaturase 1	-3.618	0.002311488
CDK18	cyclin dependent kinase 18	-3.654	5.56E-07
MYRFL	myelin regulatory factor-like	-3.669	0.007140929
PFKFB4	6-phosphofructo-2-kinase/fructose-2,6-biphosphatase 4	-3.711	0.000209785
LOC105378179	uncharacterized LOC105378179	-3.756	2.88E-09
TACC1	transforming acidic coiled-coil containing protein 1	-3.807	2.24E-05
IQGAP2	IQ motif containing GTPase activating protein 2	-3.816	0.000153473
SIRT4	sirtuin 4	-3.833	1.25E-10
ARHGEF40	Rho guanine nucleotide exchange factor 40	-3.840	0.03211052
TM4SF4	transmembrane 4 L six family member 4	-3.966	1.70E-10
PRLR	prolactin receptor	-3.995	2.00E-05
SLC29A4	solute carrier family 29 member 4	-4.043	4.10E-11
LOC102724740	uncharacterized LOC102724740	-4.052	0.021447868
LGR4	leucine rich repeat containing G protein-coupled receptor 4	-4.087	4.51E-05
PDZK1	PDZ domain containing 1	-4.106	0.046423012
PAPSS2	3'-phosphoadenosine 5'-phosphosulfate synthase 2	-4.120	1.12E-05
PLOD2	procollagen-lysine,2-oxoglutarate 5-dioxygenase 2	-4.227	3.73E-13
KCNE3	potassium voltage-gated channel subfamily E regulatory subunit 3	-4.322	0.009083405
TP53I11	tumor protein p53 inducible protein 11	-4.382	1.84E-10
NDRG1	N-myc downstream regulated 1	-4.385	4.53E-13
PTPRB	protein tyrosine phosphatase, receptor type B	-4.479	4.46E-11
CLDN2	claudin 2	-4.503	1.04E-06
CFH	complement factor H	-4.602	3.46E-09
CLU	clusterin	-4.613	0.045882073
ABCA1	ATP binding cassette subfamily A member 1	-4.628	0.004986765
PEG10	paternally expressed 10	-4.693	0.033230131
GRAMD1B	GRAM domain containing 1B	-4.833	1.50E-14
PPP1R1B	protein phosphatase 1 regulatory inhibitor subunit 1B	-4.866	0.032911557
SLC11A2	solute carrier family 11 member 2	-4.922	3.95E-15
GJB1	gap junction protein beta 1	-4.994	0.023163277
HNF1A-AS1	HNF1A antisense RNA 1	-5.032	0.038340167
RNF183	ring finger protein 183	-5.279	0.035896831
PLXND1	plexin D1	-5.468	6.02E-06
CD109	CD109 molecule	-5.516	0.013816545
PLCB4	phospholipase C beta 4	-5.627	0.038805614
ANTXR1	anthrax toxin receptor 1	-5.678	0.031171923
MGAT3	mannosyl (beta-1,4-)-glycoprotein beta-1,4-N-acetylglucosaminyltransferase	-5.718	0.000874274
WWTR1	WW domain containing transcription regulator 1	-5.749	0.002603325
CDH17	cadherin 17	-5.753	0.001298533
TPPP3	tubulin polymerization promoting protein family member 3	-5.761	8.25E-05
DNAJC22	DnaJ heat shock protein family (Hsp40) member C22	-5.793	0.005732023
EGLN3	egl-9 family hypoxia inducible factor 3	-5.839	3.97E-07
C1S	complement C1s	-5.910	0.026032097
TNS1	tensin 1	-5.965	5.04E-16
DHRS3	dehydrogenase/reductase 3	-5.975	8.80E-07
STRA6	stimulated by retinoic acid 6	-6.021	0.001648149
COLCA1	colorectal cancer associated 1	-6.082	0.007537002
LOC105370256	uncharacterized LOC105370256	-6.142	0.000120877
C3	complement C3	-6.366	3.90E-09
CXCL5	C-X-C motif chemokine ligand 5	-6.485	1.50E-14
ADRA2A	adrenoceptor alpha 2A	-6.502	0.00214777
OSMR	oncostatin M receptor	-6.546	0.007723224
GPRIN3	GPRIN family member 3	-6.602	0.015333478
PPFIA4	PTPRF interacting protein alpha 4	-6.718	0.017951726
ABCC2	ATP binding cassette subfamily C member 2	-6.719	3.11E-12
BMPR1B	bone morphogenetic protein receptor type 1B	-6.739	0.010647227
AXL	AXL receptor tyrosine kinase	-6.786	0.026028138
SLITRK6	SLIT and NTRK like family member 6	-6.994	8.30E-06
RAB37	RAB37, member RAS oncogene family	-7.004	0.002311488

PIGZ	phosphatidylinositol glycan anchor biosynthesis class Z	-7.108	0.000209785
DIP2C	disco interacting protein 2 homolog C	-7.109	0.016878127
FADS2	fatty acid desaturase 2	-7.147	3.83E-05
CNTNAP3	contactin associated protein-like 3	-7.173	0.012861253
MAP1B	microtubule associated protein 1B	-7.198	3.77E-15
COL5A2	collagen type V alpha 2 chain	-7.207	0.00206874
ST8SIA4	ST8 alpha-N-acetyl-neuraminide alpha-2,8-sialyltransferase 4	-7.526	0.003907819
ASCL2	achaete-scute family bHLH transcription factor 2	-7.571	0.035225575
CNTN1	contactin 1	-7.673	3.69E-16
MOGAT3	monoacylglycerol O-acyltransferase 3	-7.745	0.02414016
LRP4	LDL receptor related protein 4	-7.755	0.021477372
PCDH9	protocadherin 9	-7.858	0.004183485
OBSL1	obscurin like 1	-7.924	0.009948122
APLP1	amyloid beta precursor like protein 1	-7.935	0.005655414
CALD1	caldesmon 1	-7.993	0.004566192
SPP1	secreted phosphoprotein 1	-8.019	8.91E-06
SYT1	synaptotagmin 1	-8.028	0.002311488
MUC1	mucin 1, cell surface associated	-8.258	3.55E-05
NRG1	neuregulin 1	-8.287	0.003395438
EYA4	EYA transcriptional coactivator and phosphatase 4	-8.413	0.004351272
CNNM1	cyclin and CBS domain divalent metal cation transport mediator 1	-8.701	0.002571225
MYO7B	myosin VIIB	-8.874	3.97E-05
CPLX2	complexin 2	-9.136	9.87E-16
NBEA	neurobeachin	-9.165	0.010971761
TMEM74B	transmembrane protein 74B	-9.208	0.038336674
SNURF	SNRPN upstream reading frame	-9.289	0.034415237
IHH	indian hedgehog	-9.380	0.026032097
EDN1	endothelin 1	-9.433	0.001285415
FGFR1	fibroblast growth factor receptor 1	-9.580	0.023069421
ANGPT4	angiopoietin 4	-9.664	0.002469733
LOC100505817	uncharacterized LOC100505817	-9.692	0.002571225
THSD7A	thrombospondin type 1 domain containing 7A	-9.713	6.82E-05
PRICKLE2	prickle planar cell polarity protein 2	-9.849	0.005237809
C2orf72	chromosome 2 open reading frame 72	-9.871	0.004286404
ANXA13	annexin A13	-9.976	0.000881513
BASP1	brain abundant membrane attached signal protein 1	-10.054	0.00188112
BNIP3	BCL2 interacting protein 3	-10.074	0.001034732
CCDC183	coiled-coil domain containing 183	-10.380	0.032911557
NINL	ninein like	-10.390	0.045882073
ELOVL5	ELOVL fatty acid elongase 5	-10.395	0.003173521
HHLA2	HERV-H LTR-associating 2	-10.553	0.001371069
CNTNAP3B	contactin associated protein-like 3B	-10.631	0.00223658
EVC	EvC ciliary complex subunit 1	-10.752	0.009443983
IGFBP1	insulin like growth factor binding protein 1	-10.754	0.002001995
UCHL1	ubiquitin C-terminal hydrolase L1	-11.158	2.24E-05
NDUFA4L2	NDUFA4, mitochondrial complex associated like 2	-11.687	1.73E-15
KRASP1	KRAS proto-oncogene, GTPase pseudogene 1	-11.693	0.04653213
COL4A5	collagen type IV alpha 5 chain	-11.992	0.000152035
TET1	tet methylcytosine dioxygenase 1	-12.218	0.001607259
NXPH4	neurexophilin 4	-12.468	5.40E-05
THBD	thrombomodulin	-12.926	0.008928496
DDX3Y	DEAD-box helicase 3, Y-linked	-13.092	0.001052232
COL27A1	collagen type XXVII alpha 1 chain	-13.158	0.000237724
NR3C1	nuclear receptor subfamily 3 group C member 1	-13.212	0.00015704
SSPO	SCO-spondin	-14.006	0.006665758
CA9	carbonic anhydrase 9	-14.081	1.76E-30
ZNF618	zinc finger protein 618	-14.119	0.000115722
PKDCC	protein kinase domain containing, cytoplasmic	-14.170	0.007535705
SLC4A4	solute carrier family 4 member 4	-14.193	0.044891944
PIGR	polymeric immunoglobulin receptor	-14.312	1.00E-06
RAB34	RAB34, member RAS oncogene family	-14.450	0.045108008
TBX18	T-box 18	-14.510	8.61E-05
HAVCR1	hepatitis A virus cellular receptor 1	-14.544	6.81E-05
GPA33	glycoprotein A33	-15.053	0.0071381
SETBP1	SET binding protein 1	-15.148	0.001323852
NEBL	nebulette	-15.487	0.020077019

FMOD	fibromodulin	-15.793	0.007462809
PPP1R3C	protein phosphatase 1 regulatory subunit 3C	-15.948	0.02377648
SCN9A	sodium voltage-gated channel alpha subunit 9	-16.747	0.000461949
FN1	fibronectin 1	-17.058	1.14E-44
FGG	fibrinogen gamma chain	-17.363	2.93E-05
ITGA5	integrin subunit alpha 5	-17.511	0.000224205
VTN	vitronectin	-17.548	4.95E-05
IGF2BP1	insulin like growth factor 2 mRNA binding protein 1	-17.728	1.98E-05
HYAL1	hyaluronoglucosaminidase 1	-17.993	0.026032097
C20orf194	chromosome 20 open reading frame 194	-18.863	9.54E-05
QKI	QKI, KH domain containing RNA binding	-19.053	8.61E-05
FGL1	fibrinogen like 1	-19.207	1.43E-05
TMEM169	transmembrane protein 169	-19.684	0.01594565
CPPED1	calcineurin like phosphoesterase domain containing 1	-20.140	0.001209375
VIM	vimentin	-20.431	9.33E-19
SLC2A3	solute carrier family 2 member 3	-20.670	1.31E-05
CP	ceruloplasmin	-21.907	2.08E-30
TM4SF18	transmembrane 4 L six family member 18	-22.504	0.000200518
LOC105370503	uncharacterized LOC105370503	-22.566	0.002874584
MUC5B	mucin 5B, oligomeric mucus/gel-forming	-23.321	4.75E-51
FCGBP	Fc fragment of IgG binding protein	-23.503	1.36E-05
CPS1	carbamoyl-phosphate synthase 1	-23.519	2.16E-06
TTLL6	tubulin tyrosine ligase like 6	-23.715	2.05E-06
SLFN11	schlafen family member 11	-24.382	0.042924175
LOXL2	lysyl oxidase like 2	-24.671	0.02414016
RNF217	ring finger protein 217	-24.725	6.94E-05
TMEM246	transmembrane protein 246	-24.914	0.027986129
RNASE4	ribonuclease A family member 4	-27.113	6.02E-06
LRRK2	leucine rich repeat kinase 2	-28.056	1.51E-05
MPDZ	multiple PDZ domain crumbs cell polarity complex component	-32.482	0.000950561
ALPK2	alpha kinase 2	-33.915	2.05E-06
FGA	fibrinogen alpha chain	-35.261	2.04E-09
IGFBP3	insulin like growth factor binding protein 3	-38.764	3.41E-53
STC1	stanniocalcin 1	-43.303	1.52E-05
TEN1-CDK3	TEN1-CDK3 readthrough (NMD candidate)	-50.116	0.031842117
FXYD2	FXYD domain containing ion transport regulator 2	-80.144	2.02E-08
ACTG1P10	actin gamma 1 pseudogene 10	-82.553	0.001803809
UGT1A4	UDP glucuronosyltransferase family 1 member A4	-88.100	0.00066772
TIMD4	T-cell immunoglobulin and mucin domain containing 4	-88.838	0.034415237
LOC400927-CSNK1E	LOC400927-CSNK1E readthrough	-89.990	0.000438762
HSPA12A	heat shock protein family A (Hsp70) member 12A	-94.060	0.013267691
KCNE4	potassium voltage-gated channel subfamily E regulatory subunit 4	-97.994	0.014052393
SPX	spexin hormone	-111.060	0.004986765
FGB	fibrinogen beta chain	-111.402	7.09E-10
PDE4B	phosphodiesterase 4B	-111.960	0.006245259
PHOSPHO2-KLHL23	PHOSPHO2-KLHL23 readthrough	-117.579	2.81E-05
KLF7	Kruppel like factor 7	-119.444	0.00206874
LOC100129550	uncharacterized LOC100129550	-135.133	1.34E-05
LINC00473	long intergenic non-protein coding RNA 473	-136.155	0.000678652
USP9Y	ubiquitin specific peptidase 9, Y-linked	-176.125	1.03E-05
UGT1A1	UDP glucuronosyltransferase family 1 member A1	-180.312	9.66E-07
PNMA2	paraneoplastic Ma antigen 2	-184.355	2.56E-06
ANPEP	alanyl aminopeptidase, membrane	-211.352	2.66E-07
STC2	stanniocalcin 2	-368.339	7.60E-10

[1] Significant differences between the Flight and Ground cultures were determined according to an FDR < 0.05 and a minimum log2 fold change of 1 or -1 (2-fold increase or decrease in expression, respectively). Log2-fold change values were converted to fold change in this table. Red shading indicates upregulation in the Flight culture, blue shading downregulation in the Flight culture.

Supplementary Table 2. Enrichment analysis of uninfected Flight versus uninfected Ground HT-29 cultures[1]

Category	Term	Count	%	Genes	Fold enrichment	P-value	$-log_{10}$ Benjamini
**UPREGULATED**							
Biological Process	GO:0060337 type I interferon signaling pathway	5	11.11	*OASL, OAS3, SAMHD1, RSAD2, MX1*	32.80	1.43E-05	2.388345951
Biological Process	GO:0051607 defense response to virus	6	13.33	*IFNL1, OASL, OAS3, SAMHD1, RSAD2, MX1*	15.27	3.79E-05	2.265871718
Biological Process	GO:0008544 epidermis development	5	11.11	*SPRR1A, SPRR1B, SPRR3, GRHL3, LAMC2*	24.69	4.39E-05	2.377336673
Biological Process	GO:0045071 negative regulation of viral genome replication	4	8.89	*OASL, OAS3, RSAD2, MX1*	41.98	1.08E-04	2.113091102
Biological Process	GO:0006260 DNA replication	5	11.11	*CLSPN, RRM2, KIAA0101, MCM10, BRCA1*	13.54	4.47E-04	1.596560713
Biological Process	GO:0030216 keratinocyte differentiation	4	8.89	*EREG, SPRR1A, SPRR1B, SPRR3*	22.09	7.24E-04	1.467753718
**DOWNREGULATED**							
Cellular Component	GO:0005615 extracellular space	45	17.18	*FMOD, BTD, CXCL5, C3, CLU, EDN1, CD109, VTN, ANPEP, ALDH3A1, FGG, SPX, FGA, FGB, ENO2, CFH, CEACAM6, LOXL2, NRG1, MUC13, SPP1, ANGPT4, IHH, FN1, MUC1, HYAL1, STC2, AXL, IGF2, PIGR, TCN2, SSPO, PROM1, THBD, F5, SEMA4G, ANXA13, STC1, CP, IGFBP1, LRRK2, AGR2, IGFBP3, MUC5B, IGFBP4*	2.54	1.49E-08	5.399741954
Cellular Component	GO:0016324 apical plasma membrane	18	6.87	*MUC1, OSMR, MPDZ, ANO1, PDE4D, CFTR, PTPRO, PROM1, SLC11A2, SLC29A4, ANXA13, STC1, ABCC2, NRG1, PDZK1, MUC13, FN1, KCNE4*	4.70	3.16E-07	4.373333213
Cellular Component	GO:0009986 cell surface	24	9.16	*PTPRJ, PPFIA4, CLU, CD109, AXL, IQGAP2, CFTR, SLC11A2, PROM1, FGG, THBD, TNS1, FGA, PRLR, FGB, CDH17, ITGA5, GPA33, ABCC2, ANTXR1, CD24, SLITRK6, LRP4, F2R*	3.36	8.01E-07	4.145184179
Cellular Component	GO:0070062 extracellular exosome	66	25.19	*GDA, ARSD, BTD, MYO7B, ANO1, UCHL1, IQGAP2, VTN, ANPEP, TSPAN8, PLOD2, SLC2A3, CFH, SLC4A4, MUC13, PTPRJ, PRKCA, HYAL1, VIL1, CFTR, BASP1, PIGR, TCN2, PTPRO, MYADM, NEBL, CLDN2, CNTN1, GPA33, LRRK2, FXYD2, TPPP3, C3, VIM, CLU, C1S, UGT1A9, FGG, FGA, FGB, ENO2, NDRG1, FGL1, FN1, SPP1, MUC1, CPPED1, RNASE4, AXL, HGD, IGF2, DPYSL2, AK4, PROM1, THSD7A, HSPA12A, FREM2, RAB34, ANXA13, QPRT, FCGBP, CP, ANTXR1, IGFBP3, PDZK1, MUC5B*	1.78	1.92E-06	3.890264065
Cellular Component	GO:0005577 fibrinogen complex	5	1.91	*FGG, FGA, FGB, FGL1, FN1*	42.19	3.45E-06	3.732805436
Cellular Component	GO:0031093 platelet alpha granule lumen	7	2.67	*FGG, F5, FGA, FGB, CLU, IGF2, FN1*	9.66	8.09E-05	2.442616639
Cellular Component	GO:0072562 blood microparticle	10	3.82	*FGG, FGA, FGB, C3, CLU, CFH, VTN, C1S, CP, FN1*	5.00	1.82E-04	2.157871705
KEGG pathway	hsa04610: Complement and coagulation cascades	9	3.44	*FGG, THBD, F5, FGA, FGB, C3, CFH, C1S, F2R*	8.55	8.12E-06	2.828325559

[1] Differentially expressed upregulated and downregulated human transcripts between uninfected Flight and Ground cultures were analyzed using DAVID 6.8 using a threshold count of 2, an EASE score of 0.05 and Benjamini-Hochberg correction (< 0.05)

Supplementary Table 3. Differentially expressed host proteins in STL-IMMUNE[1]

Protein identifier	Description	Fold Change	P-Value
**Uninfected only: Flight vs. Ground**			
TBA1C_HUMAN	Tubulin alpha-1C chain	4.20	0.0417
ACTG_HUMAN	Actin, cytoplasmic 2	3.98	0.01083
HSPA1A_HUMAN	Heat shock 70 kDa protein 1A	2.42	0.047
**Flight only: Infected vs. Uninfected**			
K2C8_HUMAN	Keratin, type II cytoskeletal 8	- 11.37	1.3E-04
K1C18_HUMAN	Keratin, type I cytoskeletal 18	- 5.86	0.00232
AL1A1_HUMAN	Retinal dehydrogenase 1	- 11.69	0.02619
TBA1C_HUMAN	Tubulin alpha-1C chain	- 11.91	0.02741
ACTN4_HUMAN	Alpha-actinin-4	- 16.15	0.03009
**Ground only: Infected vs. Uninfected**			
PDIA3_HUMAN	Protein disulfide-isomerase A3	5.10	5.37E-03
ROA2_HUMAN	Heterogeneous ribonucleoproteins A2/B1	4.65	0.01290
S10A6_HUMAN	Protein S100-A6	3.90	0.04558
CH10_HUMAN	10 kDa heat shock protein, mitochondrial	3.87	0.04361
K2C8_HUMAN	Keratin, type II cytoskeletal 8	3.80	5.25E-05
GRP78_HUMAN	78 kDa glucose-related protein	3.63	0.01112
K1C18_HUMAN	Keratin, type I cytoskeletal 18	3.25	3.05E-06
HSPA1A_HUMAN	Heat shock 70 kDa protein 1A	3.01	0.0121
K1C19_HUMAN	Keratin, type I cytoskeletal 19	3.37	0.04404
ITIH2_HUMAN	Inter-alpha-trypsin inhibitor heavy chain	- 18.53	2.04E-04
FETUA_HUMAN	Alpha-2-HS-glycoprotein	- 9.20	0.01541
A2MG_HUMAN	Alpha-2-macroglobulin	- 8.47	6.00E-03
CO3_HUMAN	Complement C3	- 28.32	0.03718
**Infected only: Flight vs. Ground**			
K2C8_HUMAN	Keratin, type II cytoskeletal 8	- 18.72	6.05E-07
K1C18_HUMAN	Keratin, type I cytoskeletal 18	- 10.10	4.07E-05
AL1A1_HUMAN	Retinal dehydrogenase 1	- 13.07	4.52E-03
HSPA1A_HUMAN	Heat shock 70 kDa protein 1A	- 2.55	4.32E-03
H4_HUMAN	Histone H4	- 11.80	7.19E-03
PDIA3_HUMAN	Protein disulfide-isomerase A3	- 10.10	9.87E-03
A2MG_HUMAN	Alpha-2-macroglobulin	5.59	0.0142
CO3_HUMAN	Complement C3	13.80	0.0479

[1] Relative expression levels of significantly increased or decreased proteins in response to flight and/or infection ($p < 0.05$; two-tailed ANOVA). Ratios are plotted as fold changes: red shading indicates increased protein expression in the flight cultures (for the flight vs ground comparisons) or the infected culture (for infected vs uninfected comparisons). Likewise, blue shading represents decreased protein expression. Differential proteins were identified with at least 3 unique peptides (>95% confidence, 1% FDR). Additional information on the identified proteins is given in Supplemental Material, Table 11.

Supplementary Table 4. Differentially expressed host genes in Flight infected vs uninfected cultures[1]

Blue text highlights genes that were also regulated in the Ground infection and show the same trend
Red text highlights genes that were also regulated in the Ground infection but show the opposite trend

Gene	Description	Fold Change	Unique to Flight infection	FDR
**Upregulated genes (40)**				
PHOSPHO2-KLHL23	PHOSPHO2-KLHL23 readthrough	158.303		4.68E-07
UGT1A4	UDP glucuronosyltransferase family 1 member A4	146.645	X	2.31E-06
LOC400927-CSNK1E	LOC400927-CSNK1E readthrough	123.227	X	1.06E-05
EEF1E1-BLOC1S5	EEF1E1-BLOC1S5 readthrough (NMD candidate)	100.030	X	0.003979554
FKBP1A-SDCBP2	FKBP1A-SDCBP2 readthrough (NMD candidate)	93.738	X	0.017759244
TEN1-CDK3	TEN1-CDK3 readthrough (NMD candidate)	65.932	X	0.003811145
CCL20	C-C motif chemokine ligand 20	53.767		1.20E-09
CXCL1	C-X-C motif chemokine ligand 1	24.925		9.02E-51
IL17C	interleukin 17C	23.697	X	5.87E-06
HOXA10-HOXA9	HOXA10-HOXA9 readthrough	22.598		0.034598034
CXCL3	C-X-C motif chemokine ligand 3	22.509		3.69E-14
MIR3142HG	MIR3142 host gene	22.090		9.46E-06
SSPO	SCO-spondin	12.899		0.005956134
NANOGP2	Nanog homeobox pseudogene 2	10.569	X	0.001183961
LOC103344931	uncharacterized LOC103344931	10.279	X	0.002603989
CXCL2	C-X-C motif chemokine ligand 2	9.040		1.03E-15
LOC105378179	uncharacterized LOC105378179	8.699		4.75E-25
CXCL8	C-X-C motif chemokine ligand 8	7.919		8.68E-25
LINC01296	long intergenic non-protein coding RNA 1296	7.606	X	0.016665007
PAK6	p21 (RAC1) activated kinase 6	6.306	X	0.032768093
IL32	interleukin 32	5.454	X	0.044197545
LOC107987206	uncharacterized LOC107987206	4.124		3.31E-12
PI3	peptidase inhibitor 3	4.032	X	9.89E-13
TNFAIP3	TNF alpha induced protein 3	3.768		1.06E-05
SIRT4	sirtuin 4	3.748	X	7.51E-11
ICAM1	intercellular adhesion molecule 1	3.472		8.39E-10
NFKBIZ	NFKB inhibitor zeta	3.211		8.44E-09
BIRC3	baculoviral IAP repeat containing 3	3.072		1.93E-07
TNFAIP2	TNF alpha induced protein 2	2.761		5.27E-06
BCL3	B-cell CLL/lymphoma 3	2.739	X	0.000539408
ZC3H12A	zinc finger CCCH-type containing 12A	2.713		1.88E-06
SDC4	syndecan 4	2.702		1.56E-05
SOD2	superoxide dismutase 2, mitochondrial	2.303	X	0.000450759
IRAK2	interleukin 1 receptor associated kinase 2	2.241		0.003282668
RCAN1	regulator of calcineurin 1	2.213		0.004362187
NFKBIA	NFKB inhibitor alpha	2.179	X	0.000714059
ZC3H12C	zinc finger CCCH-type containing 12C	2.161	X	0.004128813
RELB	RELB proto-oncogene, NF-kB subunit	2.135	X	0.003401183
METTL12	methyltransferase like 12	2.120	X	0.005321945
NFKB2	nuclear factor kappa B subunit 2	2.032		0.004994745
**Downregulated genes (22)**				
N4BP2L2-IT2	N4BPL2 intronic transcript 2	-2.338	X	0.001647584
FAM25A	family with sequence similarity 25 member A	-2.443		0.032215301
IFT80	intraflagellar transport 80	-2.625	X	0.002415638
RARRES1	retinoic acid receptor responder 1	-3.385		0.017099356
SNORA53	small nucleolar RNA, H/ACA box 53	-3.398	X	0.003442609
FN1	fibronectin 1	-3.507		1.68E-05
CNTN1	contactin 1	-4.084		0.000793169
MAP1B	microtubule associated protein 1B	-4.178		0.000563371
CXCL5	C-X-C motif chemokine ligand 5	-4.850		2.92E-05
CPLX2	complexin 2	-5.262		0.000911363
MUC5B	mucin 5B, oligomeric mucus/gel-forming	-5.304		6.70E-09
IGFBP3	insulin like growth factor binding protein 3	-6.234		0.018915375
LOC105373455	uncharacterized LOC105373455	-9.804	X	0.045506961
POLR2J2	RNA polymerase II subunit J2	-11.320	X	0.044197545
PEG10	paternally expressed 10	-12.342		0.006989355

Downregulated genes (continued)				
PLCL2	phospholipase C like 2	-13.103		0.049033867
NEK11	NIMA related kinase 11	-13.940	X	0.038807407
BIVM-ERCC5	BIVM-ERCC5 readthrough	-38.920		8.88E-05
LOC107984730	vesicle-associated membrane protein-associated protein A pseudogene	-47.041	X	0.022851037
ADGRL2	adhesion G protein-coupled receptor L2	-53.298		0.01206827
ARPC4-TTLL3	ARPC4-TTLL3 readthrough	-56.646		0.027026184
KLHL23	kelch like family member 23	-90.467		0.000100019

[1] Significant differences between the infected and uninfected Flight cultures were determined according to an FDR < 0.05 and a minimum log2 fold change of 1 or -1 (2-fold increase or decrease in expression, respectively). Log2-fold change values were converted to fold change in this table. Red shading indicates upregulation and blue shading indicates downregulation in the infected cultures relative to the uninfected cultures.

Supplementary Table 5. Enrichment analysis of Flight HT-29 cultures: infected versus uninfected[1]

Blue text indicates GO terms/pathways that were unique to the Flight infection. Black text indicates terms/pathways that were commonly regulated between the Flight and Ground infection.

Category	Term	Count	%	Genes	Fold enrichment	P-value	$-log_{10}$ Benjamini
**UPREGULATED**							
Biological Process	GO:0006954 inflammatory response	12	30	*CXCL1, IL17C, IRAK2, NFKBIZ, CCL20, CXCL3, RELB, CXCL2, CXCL8, ZC3H12A, NFKB2, TNFAIP3*	17.15	2.49E-11	7.990517998
Biological Process	GO:0007249 I-kappaB kinase/NF-kappaB signaling	5	12.5	*IRAK2, RELB, BCL3, NFKB2, BIRC3*	45.14	3.76E-06	3.112680743
Biological Process	GO:0070098 chemokine-mediated signaling pathway	5	12.5	*CXCL1, CCL20, CXCL3, CXCL2, CXCL8*	38.15	7.40E-06	2.995336528
Biological Process	GO:0090023 positive regulation of neutrophil chemotaxis	4	10	*CXCL1, CXCL3, CXCL2, CXCL8*	98.49	7.74E-06	3.100434179
Biological Process	GO:0071222 cellular response to lipopolysaccharide	5	12.5	*ICAM1, CCL20, CXCL8, ZC3H12A, TNFAIP3*	23.97	4.65E-05	2.41909551
Biological Process	GO:0002237 response to molecule of bacterial origin	3	7.5	*CXCL2, CXCL8, TNFAIP3*	180.56	1.10E-04	2.124716028
Biological Process	GO:0032496 response to lipopolysaccharide	5	12.5	*CXCL1, CXCL3, CXCL2, NFKB2, SOD2*	16.51	1.97E-04	1.939953555
Biological Process	GO:0071347 cellular response to interleukin-1	4	10	*ICAM1, CCL20, CXCL8, ZC3H12A*	30.52	2.71E-04	1.860359033
Biological Process	GO:0032088 negative regulation of NF-kappaB transcription factor activity	4	10	*IRAK2, NFKBIA, ZC3H12A, TNFAIP3*	30.52	2.71E-04	1.860359033
Biological Process	GO:0006955 immune response	6	15	*CXCL1, CCL20, CXCL3, CXCL2, CXCL8, IL32*	7.72	8.22E-04	1.434736794
Biological Process	GO:0071356 cellular response to tumor necrosis factor	4	10	*ICAM1, CCL20, CXCL8, ZC3H12A*	19.70	9.76E-04	1.40611286
Biological Process	GO:0071356 chemotaxis	4	10	*CXCL1, CCL20, CXCL2, CXCL8*	17.76	0.00131636 6	1.319563934
Cellular Component	GO:0005615 extracellular space	10	25	*CXCL1, IL17C, ICAM1, CCL20, CXCL3, CXCL2, CXCL8, IL32, SSPO, TNFAIP2*	4.51	1.65E-04	2.093054837
Molecular Function	GO:0008009 chemokine activity	5	12.5	*CXCL1, CCL20, CXCL3, CXCL2, CXCL8*	55.57	1.62E-06	3.840056134
Molecular Function	GO:0045236 CXCR chemokine receptor binding	3	7.5	*CXCL1, CXCL3, CXCL2*	181.52	1.09E-04	2.314981649
KEGG pathway	hsa04668 TNF signaling pathway	9	22.5	*CXCL1, ICAM1, CCL20, CXCL3, CXCL2, BCL3, NFKBIA, BIRC3, TNFAIP3*	28.93	1.73E-10	7.870819522
KEGG pathway	hsa04064 NF-kappa B signaling pathway	7	17.5	*ICAM1, RELB, CXCL8, NFKBIA, NFKB2, BIRC3, TNFAIP3*	27.67	8.18E-08	5.496342934
KEGG pathway	hsa05134 Legionellosis	6	15	*CXCL1, CXCL3, CXCL2, CXCL8, NFKBIA, NFKB2*	38.22	2.64E-07	5.163519008
KEGG pathway	hsa04621 NOD-like receptor signaling pathway	6	15	*CXCL1, CXCL2, CXCL8, NFKBIA, BIRC3, TNFAIP3*	36.85	3.18E-07	5.20792802
KEGG pathway	hsa04062 Chemokine signaling pathway	6	15	*CXCL1, CCL20, CXCL3, CXCL2, CXCL8, NFKBIA*	11.10	1.17E-04	2.739140509
KEGG pathway	hsa05169 Epstein-Barr virus infection	5	12.5	*ICAM1, RELB, NFKBIA, NFKB2, TNFAIP3*	14.10	2.97E-04	2.414067009
KEGG pathway	hsa05132 Salmonella infection	4	10	*CXCL1, CXCL3, CXCL2, CXCL8*	16.58	0.00142768 6	1.801512825
KEGG pathway	hsa04060 Cytokine-cytokine receptor interaction	5	12.5	*CXCL1, CCL20, CXCL3, CXCL2, CXCL8*	7.08	0.00387271 6	1.430326755
**DOWNREGULATED**							
No GO terms or KEGG pathways were enriched for downregulated genes							

[1] Differentially expressed upregulated and downregulated human transcripts between infected and uninfected Flight cultures were analyzed using DAVID 6.8 using a threshold count of 2, an EASE score of 0.05 and Benjamini-Hochberg correction (< 0.05)

Supplementary Table 6. Differentially expressed host genes in Ground infected vs uninfected cultures[1]

Blue text highlights genes that were also regulated in the Flight infection and show the same trend
Red text highlights genes that were also regulated in the Flight infection but show the opposite trend

Gene	Description	Fold Change	Unique to Ground infection	FDR
Upregulated genes (59)				
KLHL23	kelch like family member 23	189.944		6.42E-07
SLMO2-ATP5E	SLMO2-ATP5E readthrough	118.513	X	8.37E-05
CERS6-AS1	CERS6 antisense RNA 1	118.243	X	0.00090825
BIVM-ERCC5	BIVM-ERCC5 readthrough	110.615		3.66E-06
CXCL6	C-X-C motif chemokine ligand 6	110.465	X	0.00025661
UGT1A5	UDP glucuronosyltransferase family 1 member A5	106.395	X	0.00018975
RAB4B-EGLN2	RAB4B-EGLN2 readthrough (NMD candidate)	98.631	X	0.0344097
SENP3-EIF4A1	SENP3-EIF4A1 readthrough (NMD candidate)	96.638	X	0.00150814
NPHP3-ACAD11	NPHP3-ACAD11 readthrough (NMD candidate)	93.435	X	0.00102942
NME1-NME2	NME1-NME2 readthrough	74.817	X	0.00398118
LOC107985867	uncharacterized LOC107985867	71.791	X	0.04166343
LOC642862	Ras homolog enriched in brain pseudogene	66.202	X	0.00835659
LOC107985909	uncharacterized LOC107985909	57.230	X	0.03690048
ARPC4-TTLL3	ARPC4-TTLL3 readthrough	44.944		0.02154135
CCL20	C-C motif chemokine ligand 20	41.095		5.36E-06
EEF1B2P1	eukaryotic translation elongation factor 1 beta 2 pseudogene 1	9.991	X	0.04093665
HOXA10-HOXA9	HOXA10-HOXA9 readthrough	9.851		0.00138673
CXCL1	C-X-C motif chemokine ligand 1	7.654		1.58E-22
MIR3142HG	MIR3142 host gene	7.559		0.00937465
IL4I1	interleukin 4 induced 1	7.500	X	0.00742221
RARRES1	retinoic acid receptor responder 1	4.350		2.86E-09
CXCL8	C-X-C motif chemokine ligand 8	4.297		7.21E-12
CXCL2	C-X-C motif chemokine ligand 2	4.289		6.89E-05
ICAM1	intercellular adhesion molecule 1	3.950		1.36E-11
CXCL3	C-X-C motif chemokine ligand 3	3.844		0.00367684
MRAS	muscle RAS oncogene homolog	3.621	X	2.73E-09
ZC3H12A	zinc finger CCCH-type containing 12A	3.038		1.56E-07
PIGR	polymeric immunoglobulin receptor	2.937	X	0.04162124
SDC4	syndecan 4	2.813		1.01E-05
NFKBIZ	NFKB inhibitor zeta	2.808		1.83E-06
IFNGR1	interferon gamma receptor 1	2.739	X	2.57E-06
MCAM	melanoma cell adhesion molecule	2.738	X	0.00013574
TNFAIP3	TNF alpha induced protein 3	2.616		0.03241145
STMN1	stathmin 1	2.608	X	1.44E-05
TSPAN8	tetraspanin 8	2.567	X	4.58E-05
DRAM1	DNA damage regulated autophagy modulator 1	2.532	X	7.84E-05
ATP8	ATP synthase F0 subunit 8	2.427	X	0.00027523
HIST1H2BE	histone cluster 1 H2B family member e	2.405	X	0.01499372
TLR4	toll like receptor 4	2.385	X	0.03275059
TNFAIP2	TNF alpha induced protein 2	2.302		0.0008292
CCNA2	cyclin A2	2.263	X	0.00618899
CDCA7	cell division cycle associated 7	2.241	X	0.00221468
ATP10B	ATPase phospholipid transporting 10B (putative)	2.229	X	0.00112381
LOC100287290	cytokine receptor CRL2	2.228	X	0.01649054
TRIP13	thyroid hormone receptor interactor 13	2.221	X	0.00773495
CLEC3A	C-type lectin domain family 3 member A	2.215	X	0.00046371
CENPE	centromere protein E	2.215	X	0.00831678
RCAN1	regulator of calcineurin 1	2.207		0.00835659
IRAK2	interleukin 1 receptor associated kinase 2	2.154		0.01816602
LCN2	lipocalin 2	2.150	X	0.00201172
FAM111B	family with sequence similarity 111 member B	2.109	X	0.007646
NFKB2	nuclear factor kappa B subunit 2	2.082		0.01055386
BIRC3	baculoviral IAP repeat containing 3	2.063		0.01724045
CEACAM1	carcinoembryonic antigen related cell adhesion molecule 1	2.058	X	0.00901243
KLK10	kallikrein related peptidase 10	2.053	X	0.01317932

**Upregulated genes (continued)**				
CFTR	cystic fibrosis transmembrane conductance regulator	2.042	X	0.00356923
BRCA1	BRCA1, DNA repair associated	2.039	X	0.01462255
REL	REL proto-oncogene, NF-kB subunit	2.035	X	0.00592357
PRNCR1	prostate cancer associated non-coding RNA 1	2.012	X	0.02396165
**Downregulated genes (316)**				
THSD4	thrombospondin type 1 domain containing 4	-2.002	X	0.01492104
BHLHE40	basic helix-loop-helix family member e40	-2.005	X	0.00814725
CDCP1	CUB domain containing protein 1	-2.010	X	0.01604839
APOL1	apolipoprotein L1	-2.019	X	0.00783566
FAM162A	family with sequence similarity 162 member A	-2.027	X	0.01480962
ALDOC	aldolase, fructose-bisphosphate C	-2.029	X	0.00584082
HMOX1	heme oxygenase 1	-2.045	X	0.00895015
RETSAT	retinol saturase	-2.055	X	0.01144362
RHOD	ras homolog family member D	-2.057	X	0.04569568
TDP2	tyrosyl-DNA phosphodiesterase 2	-2.075	X	0.00510779
SVIL	supervillin	-2.100	X	0.01396687
CAPRIN2	caprin family member 2	-2.105	X	0.00742221
C4orf3	chromosome 4 open reading frame 3	-2.134	X	0.00220732
EHD2	EH domain containing 2	-2.151	X	0.00220372
PDK1	pyruvate dehydrogenase kinase 1	-2.152	X	0.00521454
SLC23A2	solute carrier family 23 member 2	-2.152	X	0.00600987
LAMA3	laminin subunit alpha 3	-2.163	X	0.00315835
ALDOA	aldolase, fructose-bisphosphate A	-2.164	X	0.00615633
ABL2	ABL proto-oncogene 2, non-receptor tyrosine kinase	-2.170	X	0.00223627
MGST3	microsomal glutathione S-transferase 3	-2.170	X	0.01961326
TNFRSF10D	TNF receptor superfamily member 10d	-2.188	X	0.0443514
SDCBP2	syndecan binding protein 2	-2.190	X	0.00197154
TNS1	tensin 1	-2.194	X	0.00228339
MT1E	metallothionein 1E	-2.208	X	0.00087618
SERTAD1	SERTA domain containing 1	-2.220	X	0.00724439
MT1X	metallothionein 1X	-2.222	X	0.00064578
SLC16A3	solute carrier family 16 member 3	-2.235	X	0.0008612
SLC29A4	solute carrier family 29 member 4	-2.249	X	0.00072412
DPP4	dipeptidyl peptidase	-2.250	X	0.00891828
TP53INP2	tumor protein p53 inducible nuclear protein 2	-2.250	X	0.00129165
CDA	cytidine deaminase	-2.267	X	0.01383433
CA12	carbonic anhydrase 12	-2.270	X	0.00039074
CDC42EP3	CDC42 effector protein 3	-2.294	X	0.0027315
RHOF	ras homolog family member F, filopodia associated	-2.324	X	0.00071721
DAPK1	death associated protein kinase 1	-2.326	X	0.00053817
CEACAM5	carcinoembryonic antigen related cell adhesion molecule 5	-2.348	X	0.00037871
FUT11	fucosyltransferase 11	-2.357	X	0.0004344
CLCF1	cardiotrophin-like cytokine factor 1	-2.391	X	0.00120931
PGK1	phosphoglycerate kinase 1	-2.395	X	0.0005445
PLIN2	perilipin 2	-2.398	X	0.00020336
GAPDHP1	glyceraldehyde-3-phosphate dehydrogenase pseudogene 1	-2.410	X	0.00171216
GLS	glutaminase	-2.429	X	0.00028634
VLDLR	very low density lipoprotein receptor	-2.435	X	0.00835659
ISG20	interferon stimulated exonuclease gene 20	-2.464	X	0.00237079
KLF4	Kruppel like factor 4	-2.467	X	0.03625246
ARL2BP	ADP ribosylation factor like GTPase 2 binding protein	-2.512	X	0.00746954
P4HA1	prolyl 4-hydroxylase subunit alpha 1	-2.520	X	2.66E-05
GAB2	GRB2 associated binding protein 2	-2.540	X	0.00112381
DUSP4	dual specificity phosphatase 4	-2.558	X	2.93E-05
PTPRM	protein tyrosine phosphatase, receptor type M	-2.567	X	0.02621532
ADM	adrenomedullin	-2.580	X	0.00042375
LAMC2	laminin subunit gamma 2	-2.593	X	9.96E-05
MYO15B	myosin XVB	-2.608	X	0.00397863
ELL2	elongation factor for RNA polymerase II 2	-2.614	X	6.08E-05
NTSR1	neurotensin receptor 1	-2.633	X	3.37E-05
PTRF	polymerase I and transcript release factor	-2.642	X	4.84E-05
AK4	adenylate kinase 4	-2.656	X	0.0003872
SFN	stratifin	-2.664	X	2.72E-05
FOSL1	FOS like 1, AP-1 transcription factor subunit	-2.665	X	0.00023341
ATP2B4	ATPase plasma membrane Ca2+ transporting 4	-2.726	X	2.04E-05

Downregulated genes (continued)				
LDHA	lactate dehydrogenase A	-2.736	X	2.63E-05
NLRC5	NLR family CARD domain containing 5	-2.794	X	0.01205825
TUSC3	tumor suppressor candidate 3	-2.795	X	0.01160377
ANXA1	annexin A1	-2.861	X	4.57E-06
PLOD2	procollagen-lysine,2-oxoglutarate 5-dioxygenase 2	-2.884	X	2.26E-07
ALCAM	activated leukocyte cell adhesion molecule	-2.982	X	4.00E-05
GRB10	growth factor receptor bound protein 10	-2.985	X	0.00889671
SPIRE1	spire type actin nucleation factor 1	-2.989	X	2.49E-05
OASL	2'-5'-oligoadenylate synthetase like	-2.999	X	5.12E-06
IFIT2	interferon induced protein with tetratricopeptide repeats 2	-3.002	X	9.43E-07
GJB3	gap junction protein beta 3	-3.003	X	7.53E-05
LAT	linker for activation of T-cells	-3.145	X	0.00245147
AGR2	anterior gradient 2, protein disulphide isomerase family member	-3.193	X	2.46E-08
MID2	midline 2	-3.218	X	0.00204036
DUSP10	dual specificity phosphatase 10	-3.238	X	0.0325711
LOC645553	uncharacterized LOC645553	-3.314	X	5.08E-08
IGFBP6	insulin like growth factor binding protein 6	-3.316	X	1.15E-07
TACC1	transforming acidic coiled-coil containing protein 1	-3.422	X	0.00010096
CTSL	cathepsin L	-3.442	X	2.03E-08
LOC107987206	uncharacterized LOC107987206	-3.603		8.12E-10
ZSCAN12P1	zinc finger and SCAN domain containing 12 pseudogene 1	-3.647	X	1.41E-06
TUBB3	tubulin beta 3 class III	-3.816	X	0.04521746
BNIP3L	BCL2 interacting protein 3 like	-3.817	X	5.32E-08
AKR1C1	aldo-keto reductase family 1 member C1	-3.835	X	1.40E-09
SLPI	secretory leukocyte peptidase inhibitor	-3.847	X	0.01711276
PAG1	phosphoprotein membrane anchor with glycosphingolipid microdomains 1	-3.912	X	0.00160309
C6orf223	chromosome 6 open reading frame 223	-4.006	X	1.20E-10
SLC11A2	solute carrier family 11 member 2	-4.054	X	5.58E-12
FUT8	fucosyltransferase 8	-4.127	X	0.04599991
ALDH3A1	aldehyde dehydrogenase 3 family member A1	-4.139	X	3.51E-12
DHRS9	dehydrogenase/reductase 9	-4.156	X	1.19E-06
AKAP12	A-kinase anchoring protein 12	-4.221	X	0.00685784
P4HA2	prolyl 4-hydroxylase subunit alpha 2	-4.287	X	6.27E-13
GBE1	1,4-alpha-glucan branching enzyme 1	-4.479	X	3.39E-08
CITED2	Cbp/p300 interacting transactivator with Glu/Asp rich carboxy-terminal domain 2	-4.659	X	0.00113304
PFKFB3	6-phosphofructo-2-kinase/fructose-2,6-biphosphatase 3	-4.697	X	3.91E-15
MT2A	metallothionein 2A	-4.818	X	1.25E-14
ADGRG6	adhesion G protein-coupled receptor G6	-4.848	X	0.04234548
ENO2	enolase 2	-4.881	X	5.93E-14
LOC105378179	uncharacterized LOC105378179	-4.997		4.28E-14
FSCN1	fascin actin-bundling protein 1	-5.040	X	0.00480852
HDAC9	histone deacetylase 9	-5.060	X	0.0070314
ANXA13	annexin A13	-5.148	X	0.03690048
ZNF114	zinc finger protein 114	-5.189	X	0.01737084
PLCB4	phospholipase C beta 4	-5.266	X	0.0451635
IL1RN	interleukin 1 receptor antagonist	-5.288	X	5.92E-13
AKR1B1	aldo-keto reductase family 1 member B	-5.321	X	0.00017875
APLP1	amyloid beta precursor like protein 1	-5.363	X	0.0395862
NWD2	NACHT and WD repeat domain containing 2	-5.379	X	0.04345234
CDH17	cadherin 17	-5.708	X	0.00093411
NXPH4	neurexophilin 4	-5.720	X	0.00888243
FADS1	fatty acid desaturase 1	-5.844	X	2.59E-06
TTC28	tetratricopeptide repeat domain 28	-5.851	X	0.01686265
LOC105374003	uncharacterized LOC105374003	-5.903	X	0.01802239
AXL	AXL receptor tyrosine kinase	-5.965	X	0.04551514
KRT17	keratin 17	-6.018	X	0.00030478
UCA1	urothelial cancer associated 1 (non-protein coding)	-6.046	X	1.37E-17
EYA4	EYA transcriptional coactivator and phosphatase 4	-6.133	X	0.02043978
NCF2	neutrophil cytosolic factor 2	-6.217	X	0.00081333
CA9	carbonic anhydrase 9	-6.234	X	2.30E-17
LONRF2	LON peptidase N-terminal domain and ring finger 2	-6.325	X	0.02503702
NDRG1	N-myc downstream regulated 1	-6.333	X	2.71E-20
AHNAK2	AHNAK nucleoprotein 2	-6.590	X	8.42E-19

Downregulated genes (continued)				
EGLN3	egl-9 family hypoxia inducible factor 3	-6.646	X	2.65E-08
PFKFB4	6-phosphofructo-2-kinase/fructose-2,6-biphosphatase 4	-6.673	X	8.81E-09
RNF144A	ring finger protein 144A	-6.720	X	0.03389489
CHST11	carbohydrate sulfotransferase 11	-6.739	X	0.01964322
KIAA1462	KIAA1462	-6.960	X	6.21E-14
PTPRU	protein tyrosine phosphatase, receptor type U	-6.980	X	0.04781902
HHIPL2	HHIP like 2	-7.224	X	0.02805903
MID1	midline 1	-7.325	X	0.00033458
IL6R	interleukin 6 receptor	-7.422	X	1.73E-07
CST1	cystatin SN	-7.428	X	3.35E-18
WWTR1	WW domain containing transcription regulator 1	-7.594	X	0.00016976
C2orf72	chromosome 2 open reading frame 72	-7.609	X	0.01239241
TET1	tet methylcytosine dioxygenase 1	-7.668	X	0.01176597
SPTSSB	serine palmitoyltransferase small subunit B	-7.782	X	1.58E-08
OSMR	oncostatin M receptor	-7.791	X	0.00195094
NRP1	neuropilin 1	-7.935	X	3.70E-13
RAET1L	retinoic acid early transcript 1L	-8.289	X	0.04967792
TM4SF19	transmembrane 4 L six family member 19	-8.626	X	0.03241145
IL36RN	interleukin 36 receptor antagonist	-8.711	X	0.03246965
CAV2	caveolin 2	-8.955	X	0.00044326
LOC105375172	uncharacterized LOC105375172	-9.211	X	0.00541484
BTBD11	BTB domain containing 11	-9.268	X	0.00340314
ADGRL2	adhesion G protein-coupled receptor L2	-9.277		0.00933567
SPRR3	small proline rich protein 3	-9.623	X	3.11E-10
PDE7B	phosphodiesterase 7B	-9.683	X	0.01506336
FCGBP	Fc fragment of IgG binding protein	-9.721	X	0.00112183
MIR210HG	MIR210 host gene	-9.789	X	0.00207403
PAPPA	pappalysin 1	-10.117	X	0.00152656
GALNT5	polypeptide N-acetylgalactosaminyltransferase 5	-10.173	X	8.17E-06
ST6GAL1	ST6 beta-galactoside alpha-2,6-sialyltransferase 1	-10.262	X	0.02175511
KIAA0319	KIAA0319	-10.710	X	1.10E-05
C3	complement C3	-10.712	X	8.44E-14
PHOSPHO2-KLHL23	PHOSPHO2-KLHL23 readthrough	-10.932		0.01012828
ANGPTL4	angiopoietin like 4	-10.989	X	8.40E-08
COL6A1	collagen type VI alpha 1 chain	-11.254	X	8.53E-07
DPYSL3	dihydropyrimidinase like 3	-11.855	X	0.00170066
RAB3B	RAB3B, member RAS oncogene family	-11.875	X	0.00087627
ANPEP	alanyl aminopeptidase, membrane	-12.004	X	0.00113304
MUC16	mucin 16, cell surface associated	-12.864	X	1.77E-06
DKK1	dickkopf WNT signaling pathway inhibitor 1	-12.868	X	0.01518642
BNIP3	BCL2 interacting protein 3	-13.200	X	0.00017367
NRP2	neuropilin 2	-14.036	X	0.00835659
ARL4C	ADP ribosylation factor like GTPase 4C	-14.856	X	3.45E-12
COL5A2	collagen type V alpha 2 chain	-15.236	X	6.62E-06
RHOBTB1	Rho related BTB domain containing 1	-16.083	X	0.0031665
CD109	CD109 molecule	-16.117	X	5.66E-06
GPSM1	G-protein signaling modulator 1	-16.207	X	0.00012942
IGFBP1	insulin like growth factor binding protein 1	-16.359	X	0.00021396
CEACAM6	carcinoembryonic antigen related cell adhesion molecule 6	-16.377	X	2.67E-41
CPS1	carbamoyl-phosphate synthase 1	-17.267	X	1.27E-05
PPFIA4	PTPRF interacting protein alpha 4	-17.462	X	0.0001341
BDNF	brain derived neurotrophic factor	-18.098	X	0.00055341
BCAT1	branched chain amino acid transaminase 1	-18.732	X	0.04137557
LINC00520	long intergenic non-protein coding RNA 520	-18.863	X	0.00665282
SPP1	secreted phosphoprotein 1	-19.289	X	4.59E-10
PTPRS	protein tyrosine phosphatase, receptor type S	-19.328	X	0.00031153
HRNR	hornerin	-19.351	X	0.00663543
LGSN	lengsin, lens protein with glutamine synthetase domain	-19.485	X	0.00033458
SETBP1	SET binding protein 1	-19.753	X	0.00043959
NR3C1	nuclear receptor subfamily 3 group C member 1	-20.319		1.23E-05
PLCL2	phospholipase C like 2	-20.628		0.00016409
TM4SF18	transmembrane 4 L six family member 18	-21.166	X	0.00021202
NDUFA4L2	NDUFA4, mitochondrial complex associated like 2	-21.211	X	1.63E-20
ABCC2	ATP binding cassette subfamily C member 2	-21.425	X	3.64E-24

Downregulated genes (continued)				
CAMK1D	calcium/calmodulin dependent protein kinase ID	-21.445	X	0.03147727
CACNA1H	calcium voltage-gated channel subunit alpha1 H	-22.426	X	0.03101992
LOC102723490	uncharacterized LOC102723490	-22.547	X	0.0025194
TMEM246	transmembrane protein 246	-22.725	X	0.03275059
ITGA5	integrin subunit alpha 5	-22.855	X	7.53E-05
ZNF462	zinc finger protein 462	-23.183	X	0.00983955
RNF217	ring finger protein 217	-23.245	X	7.53E-05
BDKRB2	bradykinin receptor B2	-23.272	X	0.01108023
ZNF618	zinc finger protein 618	-24.593	X	5.60E-06
EML1	echinoderm microtubule associated protein like 1	-24.799	X	0.00556706
NAV3	neuron navigator 3	-25.058	X	3.06E-05
BASP1	brain abundant membrane attached signal protein 1	-25.295	X	2.26E-05
SYT1	synaptotagmin 1	-25.391	X	1.27E-06
NEBL	nebulette	-25.421	X	0.00575752
ZNF365	zinc finger protein 365	-26.097	X	3.60E-06
CAV1	caveolin 1	-26.821	X	0.00043959
STOM	stomatin	-27.227	X	1.23E-05
STC2	stanniocalcin 2	-27.601	X	1.84E-06
CALD1	caldesmon 1	-29.834	X	5.11E-06
VTN	vitronectin	-30.994	X	2.99E-06
SPRR1A	small proline rich protein 1A	-31.428	X	3.97E-12
NRCAM	neuronal cell adhesion molecule	-33.122	X	9.16E-10
CNNM1	cyclin and CBS domain divalent metal cation transport mediator 1	-33.659	X	1.83E-06
ANTXR1	anthrax toxin receptor 1	-34.872	X	1.81E-06
NBEA	neurobeachin	-35.223	X	7.53E-05
LRP4	LDL receptor related protein 4	-35.843	X	6.08E-05
SPRR1B	small proline rich protein 1B	-37.449	X	7.14E-37
PRICKLE2	prickle planar cell polarity protein 2	-37.696	X	2.92E-05
CHST15	carbohydrate sulfotransferase 15	-37.801	X	2.44E-05
SGK3	serum/glucocorticoid regulated kinase family member 3	-39.071	X	0.00197165
ELOVL5	ELOVL fatty acid elongase 5	-39.790	X	1.60E-05
CNTNAP3B	contactin associated protein-like 3B	-40.698	X	1.03E-05
PEG10	paternally expressed 10	-40.701		8.53E-09
LHX4-AS1	LHX4 antisense RNA 1	-41.193	X	0.03080333
CST6	cystatin E/M	-41.950	X	0.04958711
QKI	QKI, KH domain containing RNA binding	-45.291	X	3.60E-06
IGF2BP1	insulin like growth factor 2 mRNA binding protein 1	-45.485	X	1.28E-07
THSD7A	thrombospondin type 1 domain containing 7A	-48.158	X	4.87E-10
CLU	clusterin	-49.319	X	1.32E-08
MAP1B	microtubule associated protein 1B	-51.693		2.20E-36
GLI2	GLI family zinc finger 2	-55.624	X	0.02951303
DENND2C	DENN domain containing 2C	-58.618	X	0.00983955
LOC105376938	uncharacterized LOC105376938	-61.571	X	0.03301083
SLC2A3	solute carrier family 2 member 3	-63.692	X	1.15E-07
POC1B-GALNT4	POC1B-GALNT4 readthrough	-65.676	X	0.03398009
IL36B	interleukin 36, beta	-71.181	X	0.0029466
FGA	fibrinogen alpha chain	-74.507	X	2.96E-11
KRT81	keratin 81	-77.939	X	2.49E-08
UNC5C	unc-5 netrin receptor C	-78.801	X	0.01480962
VIM	vimentin	-79.670	X	6.83E-27
PWAR5	Prader Willi/Angelman region RNA 5	-84.035	X	0.03275059
TM4SF20	transmembrane 4 L six family member 20	-87.285	X	0.03718025
NTRK3	neurotrophic receptor tyrosine kinase 3	-88.269	X	0.01076595
KDM5D	lysine demethylase 5D	-88.382	X	0.01349776
BEX3	brain expressed X-linked 3	-88.495	X	0.01677241
RIMKLB	ribosomal modification protein rimK like family member B	-88.616	X	0.02134754
ANKRD18B	ankyrin repeat domain 18B	-88.720	X	0.01376336
TIMD4	T-cell immunoglobulin and mucin domain containing 4	-88.838	X	0.03379727
ZNF605	zinc finger protein 605	-92.952	X	0.00851734
BPIFB1	BPI fold containing family B member 1	-93.539	X	0.02883885
FN1	fibronectin 1	-95.301		1.81E-77
TFF2	trefoil factor 2	-96.688	X	0.00036859
KCNE4	potassium voltage-gated channel subfamily E regulatory subunit 4	-97.994	X	0.01406747
TSPYL5	TSPY like 5	-98.239	X	0.02411357
PHLDB2	pleckstrin homology like domain family B member 2	-100.269	X	0.00170066

Downregulated genes (continued)				
HSPB8	heat shock protein family B (small) member 8	-100.351	X	0.00029321
TDRP	testis development related protein	-101.867	X	0.00221468
GALC	galactosylceramidase	-102.573	X	0.00895015
SIRPA	signal regulatory protein alpha	-106.261	X	0.0046571
CXCL5	C-X-C motif chemokine ligand 5	-107.246		3.91E-43
MAP9	microtubule associated protein 9	-107.265	X	0.00746954
ABCA12	ATP binding cassette subfamily A member 12	-107.269	X	0.00753868
RTEL1-TNFRSF6B	RTEL1-TNFRSF6B readthrough (NMD candidate)	-110.140	X	0.00010922
RPS4Y1	ribosomal protein S4, Y-linked 1	-111.012	X	0.00089059
SPX	spexin hormone	-111.060	X	0.00488765
ANKRD30A	ankyrin repeat domain 30A	-111.585	X	0.00269285
PDE4B	phosphodiesterase 4B	-111.960	X	0.00618375
SLFN11	schlafen family member 11	-114.975	X	0.00418215
AMOTL1	angiomotin like 1	-115.600	X	0.00057487
SLC4A4	solute carrier family 4 member 4	-119.861	X	0.00087606
CHGB	chromogranin B	-120.970	X	0.00176291
RAB34	RAB34, member RAS oncogene family	-122.055	X	0.00089059
UCHL1	ubiquitin C-terminal hydrolase L1	-124.925	X	8.04E-12
FAM127A	family with sequence similarity 127 member A	-125.537	X	0.00109587
CDH6	cadherin 6	-129.443	X	0.00018308
PPP1R3C	protein phosphatase 1 regulatory subunit 3C	-134.673	X	0.00042375
LINC00473	long intergenic non-protein coding RNA 473	-136.155	X	0.00062936
GPRIN2	G protein regulated inducer of neurite outgrowth 2	-141.783	X	5.38E-05
C8orf4	chromosome 8 open reading frame 4	-143.223	X	4.93E-05
CP	ceruloplasmin	-146.505	X	1.00E-44
SNURF	SNRPN upstream reading frame	-147.990	X	4.51E-05
RSPO3	R-spondin 3	-148.087	X	5.38E-05
FGFR1	fibroblast growth factor receptor 1	-151.781	X	2.63E-05
MPDZ	multiple PDZ domain crumbs cell polarity complex component	-152.868	X	5.13E-05
EPDR1	ependymin related 1	-156.984	X	1.27E-05
THBD	thrombomodulin	-157.366	X	2.72E-05
FADS2	fatty acid desaturase 2	-161.611	X	3.40E-16
CPPED1	calcineurin like phosphoesterase domain containing 1	-169.767	X	1.03E-05
EVC	EvC ciliary complex subunit 1	-171.180	X	8.01E-06
MUC5B	mucin 5B, oligomeric mucus/gel-forming	-175.440		2.88E-88
USP9Y	ubiquitin specific peptidase 9, Y-linked	-176.125	X	1.08E-05
COL4A6	collagen type IV alpha 6 chain	-179.304	X	1.70E-06
PNMA2	paraneoplastic Ma antigen 2	-184.355	X	2.97E-06
GABRB3	gamma-aminobutyric acid type A receptor beta3 subunit	-194.100	X	7.56E-07
LOC105374020	uncharacterized LOC105374020	-198.710	X	4.88E-07
STC1	stanniocalcin 1	-203.649	X	6.84E-07
SCN9A	sodium voltage-gated channel alpha subunit 9	-203.651	X	6.84E-07
DDX3Y	DEAD-box helicase 3, Y-linked	-208.229	X	4.19E-07
CNTNAP3	contactin associated protein-like 3	-222.186	X	1.47E-07
LOC100505817	uncharacterized LOC100505817	-226.118	X	1.22E-07
C20orf194	chromosome 20 open reading frame 194	-229.311	X	1.16E-07
LRRK2	leucine rich repeat kinase 2	-236.285	X	7.92E-08
ALPK2	alpha kinase 2	-285.625	X	9.21E-09
NRG1	neuregulin 1	-288.114	X	1.02E-08
CPLX2	complexin 2	-322.533		9.36E-40
BMPR1B	bone morphogenetic protein receptor type 1B	-333.784	X	3.77E-09
FGG	fibrinogen gamma chain	-339.996	X	2.13E-09
TBX18	T-box 18	-340.028	X	2.30E-09
FAM25A	family with sequence similarity 25 member A	-349.578		6.41E-16
PCDH9	protocadherin 9	-362.916	X	1.84E-09
FXYD2	FXYD domain containing ion transport regulator 2	-376.908	X	8.01E-10
FGL1	fibrinogen like 1	-377.799	X	9.01E-10
HAVCR1	hepatitis A virus cellular receptor 1	-395.865	X	6.62E-10
ST8SIA4	ST8 alpha-N-acetyl-neuraminide alpha-2,8-sialyltransferase 4	-433.561	X	2.61E-10
COL4A5	collagen type IV alpha 5 chain	-462.698	X	1.24E-10
TTLL6	tubulin tyrosine ligase like 6	-466.552	X	1.15E-10
FGB	fibrinogen beta chain	-523.943	X	2.63E-11
CNTN1	contactin 1	-1831.878		8.47E-49
IGFBP3	insulin like growth factor binding protein 3	-3217.287		4.52E-75

[1] Significant differences between Ground infected and uninfected cultures were determined according to an FDR < 0.05 and a minimum log2 fold change of 1 or -1 (2-fold increase or decrease in expression, respectively). Log2-fold change values were converted to fold change in this table. Red shading indicates upregulation and blue shading indicates downregulation in the infected cultures relative to the uninfected cultures.

Supplementary Table 7. Enrichment analysis of Ground HT-29 cultures: infected versus uninfected[1]

Blue text indicates GO terms/pathways that were unique to the Ground infection. Black text indicates terms/pathways that were commonly regulated between the Flight and Ground infection.

Category	Term	Count	%	Genes	Fold enrichment	P-value	$-\log_{10}$ Benjamini
**UPREGULATED**							
Biological Process	GO:0006954 inflammatory response	13	22.03	*IRAK2, CXCL1, NFKBIZ, CXCL3, CXCL2, CXCL8, TLR4, NFKB2, CXCL6, CCL20, REL, ZC3H12A, TNFAIP3*	12.80	1.62E-10	7.075292654
Biological Process	GO:0071222 cellular response to lipopolysaccharide	7	11.86	*LCN2, ICAM1, CCL20, CXCL8, ZC3H12A, TLR4, TNFAIP3*	23.12	4.66E-07	3.916979441
Biological Process	GO:0070098 chemokine-mediated signaling pathway	6	10.17	*CXCL1, CCL20, CXCL3, CXCL2, CXCL8, CXCL6*	31.53	1.12E-06	3.712328744
Biological Process	GO:0071356 cellular response to tumor necrosis factor	6	10.17	*LCN2, ICAM1, CCL20, CXCL8, ZC3H12A, BRCA1*	20.35	9.75E-06	2.897162531
Biological Process	GO:0007249 I-kappaB kinase/NF-kappaB signaling	5	8.47	*IRAK2, REL, TLR4, NFKB2, BIRC3*	31.10	1.80E-05	2.728936504
Biological Process	GO:0090023 positive regulation of neutrophil chemotaxis	4	6.78	*CXCL1, CXCL3, CXCL2, CXCL8*	67.85	2.50E-05	2.66526767
Biological Process	GO:0071347 cellular response to interleukin-1	5	8.47	*LCN2, ICAM1, CCL20, CXCL8, ZC3H12A*	26.28	3.51E-05	2.584909459
Biological Process	GO:0032496 response to lipopolysaccharide	6	10.17	*CXCL1, CXCL3, CXCL2, TLR4, CXCL6, NFKB2*	13.65	6.67E-05	2.363695209
Biological Process	GO:0002237 response to molecule of bacterial origin	3	5.08	*CXCL2, CXCL8, TNFAIP3*	124.39	2.39E-04	1.863206726
Biological Process	GO:0071356 chemotaxis	5	8.47	*CXCL1, CCL20, CXCL2, CXCL8, CXCL6*	15.29	2.88E-04	1.828409596
Biological Process	GO:0060326 cell chemotaxis	4	6.78	*CXCL1, CCL20, CXCL2, CXCL6*	22.96	6.55E-04	1.515975567
Biological Process	GO:0006955 immune response	7	11.86	*CXCL1, CCL20, CXCL3, CXCL2, CXCL8, TLR4, CXCL6*	6.20	7.55E-04	1.492115608
Cellular Component	GO:0005615 extracellular space	12	20.34	*CXCL1, LCN2, ICAM1, HIST1H2BE, CCL20, CXCL3, CXCL2, CXCL8, CXCL6, PIGR, MCAM, TNFAIP2*	3.53	3.37E-04	1.484410284
Molecular Function	GO:0008009 chemokine activity	6	10.17	*CXCL1, CCL20, CXCL3, CXCL2, CXCL8, CXCL6*	46.98	1.48E-07	4.772769617
Molecular Function	GO:0045236 CXCR chemokine receptor binding	4	6.78	*CXCL1, CXCL3, CXCL2, CXCL6*	170.52	1.28E-06	4.137112083
KEGG pathway	hsa05134 Legionellosis	6	10.17	*CXCL1, CXCL3, CXCL2, CXCL8, TLR4, NFKB2*	26.36	2.11E-06	3.69257035
KEGG pathway	hsa04668 TNF signaling pathway	7	11.86	*CXCL1, ICAM1, CCL20, CXCL3, CXCL2, BIRC3, TNFAIP3*	15.52	3.51E-06	3.773534914
KEGG pathway	hsa05132 Salmonella infection	6	10.17	*CXCL1, CXCL3, CXCL2, CXCL8, TLR4, IFNGR1*	17.15	1.79E-05	3.242148363
KEGG pathway	hsa04064 NF-kappa B signaling pathway	6	10.17	*ICAM1, CXCL8, TLR4, NFKB2, BIRC3, TNFAIP3*	16.36	2.25E-05	3.267245909
KEGG pathway	hsa04621 NOD-like receptor signaling pathway	5	8.47	*CXCL1, CXCL2, CXCL8, BIRC3, TNFAIP3*	21.18	6.98E-05	2.873413913
KEGG pathway	hsa04060 Cytokine-cytokine receptor interaction	7	11.86	*CXCL1, CCL20, CXCL3, CXCL2, CXCL8, CXCL6, IFNGR1*	6.83	3.58E-04	2.243603984
KEGG pathway	hsa05323 Rheumatoid arthritis	5	8.47	*ICAM1, CCL20, CXCL8, TLR4, CXCL6*	13.48	4.05E-04	2.256552424
KEGG pathway	hsa04062 Chemokine signaling pathway	6	10.17	*CXCL1, CCL20, CXCL3, CXCL2, CXCL8, CXCL6*	7.65	8.11E-04	2.01354949
**DOWNREGULATED**							
Biological Process	GO:0007155 cell adhesion	23	7.28	*NRP2, MPDZ, CNTNAP3B, PTPRS, VTN, PTPRU, SIRPA, COL4A6, APLP1, CDH6, ALCAM, KIAA1462,*	3.02	8.60E-06	1.842462087

				LAMA3, ITGA5, CDH17, CNTNAP3, CNTN1, COL6A1, LAMC2, ABL2, SPP1, FN1, MUC16			
Biological Process	GO:0001666 response to hypoxia	13	4.11	*SLC11A2, LDHA, CAV1, ADM, CA9, PLOD2, HMOX1, EGLN3, BNIP3, DPP4, CITED2, ALDH3A1, ANGPTL4*	4.55	3.01E-05	1.601006259
Biological Process	GO:0061621 canonical glycolysis	6	1.90	*ALDOA, PFKFB4, PFKFB3, ALDOC, ENO2, PGK1*	13.89	5.93E-05	1.484628112
Biological Process	GO:0030198 extracellular matrix organization	13	4.11	*VTN, COL5A2, COL4A6, COL4A5, FGG, LAMA3, FGA, ITGA5, FGB, COL6A1, LAMC2, SPP1, FN1*	3.99	1.07E-04	1.356529113
Biological Process	GO:0031424 keratinization	7	2.22	*HRNR, KRT17, SPRR1A, SPRR1B, SPRR3, SFN, ABCA12*	8.78	1.34E-04	1.356763887
Cellular Component	GO:0005615 extracellular space	54	17.09	*NRP1, IGFBP6, VTN, ANPEP, KRT81, SPX, PAPPA, HMOX1, CEACAM6, NRG1, STC2, CST1, IL6R, STOM, CTSL, THBD, ADM, CST6, IL36B, SLPI, STC1, LAMC2, LRRK2, ALDOA, BPIFB1, CXCL5, C3, CLU, CD109, SFN, ALDH3A1, FGG, FGA, FGB, TFF2, ENO2, FN1, SPP1, ANGPTL4, IL1RN, ANXA1, AXL, DPYSL3, IL36RN, DKK1, APOL1, AKR1B1, ANXA13, CP, IGFBP1, AGR2, IGFBP3, MUC5B, MUC16*	2.52	5.81E-10	6.736047768
Cellular Component	GO:0070062 extracellular exosome	81	25.63	*LDHA, HRNR, IGFBP6, UCHL1, VTN, ANPEP, SDCBP2, CLCF1, SLC2A3, PLOD2, CEACAM5, SLC4A4, RHOF, DPP4, AKR1C1, TUBB3, FUT11, PTPRS, BASP1, SIRPA, NEBL, STOM, CTSL, CST6, KRT17, SPRR3, SLPI, CNTN1, LRRK2, ALDOA, FXYD2, RAB3B, BPIFB1, FUT8, EPDR1, C3, POC1B-GALNT4, ALDOC, VIM, CLU, SFN, CDH6, ALCAM, FGG, FGA, FGB, TFF2, GALC, ENO2, COL6A1, FAM162A, NDRG1, FGL1, EHD2, FN1, SPP1, ST6GAL1, CPPED1, IL1RN, FSCN1, AXL, ANXA1, AK4, MID2, THSD7A, MGST3, GBE1, LAMA3, SPRR1B, RAB34, AKR1B1, THSD4, ANXA13, FCGBP, ZNF114, CP, ANTXR1, PGK1, IGFBP3, MUC5B, MUC16*	1.81	5.84E-08	5.035072263
Cellular Component	GO:0005577 fibrinogen complex	5	1.58	*FGG, FGA, FGB, FGL1, FN1*	34.91	7.33E-06	3.112441292
Cellular Component	GO:0005886 plasma membrane	96	30.38	*NRP2, SYT1, TUSC3, NRP1, GABRB3, OSMR, UCHL1, CDCP1, NBEA, SDCBP2, NRCAM, ATP2B4, GAB2, SLC2A3, SLC23A2, HMOX1, CNTNAP3, RAET1L, CEACAM6, UNC5C, RHOD, SLC4A4, RHOF, DPP4, PAG1, CA12, PCDH9, PTPRS, SPIRE1, BASP1, PTPRU, IL6R, NTSR1, SIRPA, DAPK1, GRB10, THBD, CA9, TNFRSF10D, CNTN1, LRRK2, ARL4C, FXYD2, CAV2, FGFR1, CAV1, RAB3B, C3, CALD1, VIM, AKAP12, CD109, BDKRB2, APLP1, ALDH3A1, CDH6, SLC11A2, FGG, PLIN2, FGA, SLC29A4, FGB, GPSM1, ENO2, AHNAK2, RHOBTB1, LGSN, NDRG1, EHD2, CDC42EP3, FAM127A, ABCA12, RNF144A, CPPED1, IL1RN, MAP1B, ADGRG6, AXL, ANXA1, CNNM1, THSD7A, SLC16A3, LAT, DKK1, CDH17, ITGA5, SVIL, ANXA13, TM4SF20, KIAA0319,*	1.46	3.63E-05	2.543396236

				ANTXR1, ABCC2, BMPR1B, PHLDB2, MUC16, VLDLR			
Cellular Component	GO:0048471 perinuclear region of cytoplasm	25	7.91	*CAV2, HRNR, CAV1, RAB3B, PTPRM, STC2, POC1B-GALNT4, CLU, IGF2BP1, SPIRE1, MT1X, APLP1, STOM, SLC11A2, BDNF, EML1, HMOX1, RAB34, MT2A, AKR1B1, MT1E, LAMC2, NDRG1, EHD2, SPP1*	2.53	5.75E-05	2.440179073
Cellular Component	GO:0016324 apical plasma membrane	16	5.06	*CAV1, OSMR, MPDZ, ANXA1, IL6R, AMOTL1, SLC11A2, SLC29A4, SLC23A2, ANXA13, STC1, ABCC2, NRG1, DPP4, FN1, KCNE4*	3.46	6.74E-05	2.45051893
Cellular Component	GO:0005576 extracellular region	46	14.56	*ALDOA, NRP2, FGFR1, BPIFB1, NXPH4, EPDR1, CXCL5, C3, CLU, IGFBP6, VTN, CDCP1, NRCAM, BDNF, FGG, PLIN2, FGA, CLCF1, PAPPA, FGB, RSPO3, CNTNAP3, CDA, COL6A1, NRG1, SPP1, ANGPTL4, FN1, HHIPL2, STC2, ANXA1, IL6R, COL5A2, COL4A6, COL4A5, CTSL, DKK1, LAMA3, APOL1, ADM, IL36B, LAMC2, CP, IGFBP1, CHGB, IGFBP3*	1.80	1.30E-04	2.23226548
Cellular Component	GO:0072562 blood microparticle	11	3.48	*STOM, FGG, APOL1, FGA, FGB, C3, CLU, VTN, CP, FN1, ANGPTL4*	4.55	1.59E-04	2.202025537
Cellular Component	GO:0016323 basolateral plasma membrane	11	3.48	*FXYD2, CAV1, ATP2B4, CA9, CDH17, SLC23A2, ANXA1, ANXA13, CEACAM5, IL6R, SLC4A4*	3.84	6.16E-04	1.669578624
Cellular Component	GO:0001533 cornified envelope	6	1.90	*HRNR, CST6, SPRR1A, SPRR1B, ANXA1, SPRR3*	8.20	7.80E-04	1.613333839
Cellular Component	GO:0009986 cell surface	20	6.33	*PPFIA4, NRP1, CLU, ANXA1, CD109, AXL, IL6R, NTSR1, SLC11A2, FGG, THBD, TNS1, FGA, FGB, ITGA5, CDH17, ABCC2, ANTXR1, LRP4, DPP4*	2.32	0.001100515	1.506721294
Cellular Component	GO:0045121 membrane raft	11	3.48	*STOM, LAT, CAV2, CAV1, PTRF, CNTN1, ANXA13, NTSR1, PGK1, PAG1, DPP4*	3.36	0.00171468	1.35471486
Cellular Component	GO:0031093 platelet alpha granule lumen	6	1.90	*ALDOA, FGG, FGA, FGB, CLU, FN1*	6.86	0.001763319	1.376824471
Molecular Function	GO:0005102 receptor binding	19	6.01	*CAV1, NXPH4, C3, IGFBP6, ANXA1, CAPRIN2, ALCAM, FGG, LAMA3, FGA, ADM, FGB, CLCF1, RSPO3, IGFBP1, NRG1, ABL2, DPP4, ABCA12*	3.29	2.07E-05	1.96033577
Molecular Function	GO:0030674 protein binding, bridging	9	2.85	*CAV2, CAV1, FGG, FGA, SPRR1A, FGB, SPRR1B, FSCN1, ANXA1*	7.15	3.57E-05	2.024221751
KEGG pathway	hsa04512 ECM-receptor interaction	10	3.16	*LAMA3, ITGA5, COL6A1, LAMC2, VTN, COL5A2, COL4A6, SPP1, COL4A5, FN1*	5.69	5.52E-05	1.986378305

[1] Differentially expressed upregulated and downregulated human transcripts between infected and uninfected Ground cultures were analyzed using DAVID 6.8 using a threshold count of 2, an EASE score of 0.05 and Benjamini-Hochberg correction (< 0.05)

Supplementary Table 8. Differentially expressed host genes in infected Flight vs infected Ground cultures [1,2]

Gene	Description	Fold Change	FDR
**Upregulated genes (266)**			
FAM25A	family with sequence similarity 25 member A	349.858	4.73E-16
LOC103344931	uncharacterized LOC103344931	174.972	1.86E-06
TFF2	trefoil factor 2	165.359	3.20E-06
CST6	cystatin E/M	111.743	6.78E-05
HSPB8	heat shock protein family B (small) member 8	95.264	0.000275112
FKBP1A-SDCBP2	FKBP1A-SDCBP2 readthrough (NMD candidate)	93.738	0.016978697
IL36B	interleukin 36, beta	91.330	0.000328636
SPRR1B	small proline rich protein 1B	90.507	4.98E-60
LOC283045	uncharacterized LOC283045	87.262	0.000752781
IFNL1	interferon lambda 1	87.059	0.000774627
TGIF2-C20orf24	TGIF2-C20orf24 readthrough	83.971	0.014763594
SPRR1A	small proline rich protein 1A	82.378	3.44E-26
EEF1E1-BLOC1S5	EEF1E1-BLOC1S5 readthrough (NMD candidate)	78.034	0.006423799
CNTN1	contactin 1	58.459	0.00135517
LOC105373098	uncharacterized LOC105373098	54.519	0.042969066
HSPE1-MOB4	HSPE1-MOB4 readthrough	46.503	0.049391085
DENND2C	DENN domain containing 2C	46.204	0.020233506
LHX4-AS1	LHX4 antisense RNA 1	40.102	0.019069817
SPRR3	small proline rich protein 3	39.831	9.36E-38
CSF2	colony stimulating factor 2	33.856	4.08E-05
SGK3	serum/glucocorticoid regulated kinase family member 3	26.708	0.013019581
ZNF365	zinc finger protein 365	23.966	7.32E-06
IFNB1	interferon beta 1	23.921	0.000704738
LIPK	lipase family member K	20.760	1.96E-05
IGFN1	immunoglobulin-like and fibronectin type III domain containing 1	20.284	0.002406174
KIAA0319	KIAA0319	16.959	6.36E-09
PHOSPHO2-KLHL23	PHOSPHO2-KLHL23 readthrough	14.719	0.000662791
LINC00520	long intergenic non-protein coding RNA 520	14.147	0.023038258
IVL	involucrin	14.120	0.028183866
CST1	cystatin SN	14.084	3.61E-33
CAV2	caveolin 2	13.904	1.44E-06
CTNNA2	catenin alpha 2	13.889	0.000915727
RAET1L	retinoic acid early transcript 1L	13.570	0.000992228
CST4	cystatin S	13.348	0.034275804
IL36RN	interleukin 36 receptor antagonist	12.809	0.001101779
NCF2	neutrophil cytosolic factor 2	12.717	8.83E-09
GPSM1	G-protein signaling modulator 1	12.571	0.000883479
CLU	clusterin	12.008	0.007434587
LOC105378179	uncharacterized LOC105378179	11.573	1.25E-30
LOC105376517	uncharacterized LOC105376517	11.557	0.011312967
MYH15	myosin heavy chain 15	11.045	0.00503416
MAOB	monoamine oxidase B	10.502	0.002963514
AHNAK2	AHNAK nucleoprotein 2	10.412	3.44E-28
COL6A1	collagen type VI alpha 1 chain	9.924	4.26E-06
DHRS9	dehydrogenase/reductase 9	9.854	6.38E-21
SPTSSB	serine palmitoyltransferase small subunit B	9.756	4.12E-11
KIAA1462	KIAA1462	9.681	2.18E-20
KRT17	keratin 17	9.345	2.55E-07
NAV3	neuron navigator 3	9.326	0.037751375
ANGPTL4	angiopoietin like 4	8.868	2.45E-06
LINC00941	long intergenic non-protein coding RNA 941	8.554	0.003695165
GDPD3	glycerophosphodiester phosphodiesterase domain containing 3	8.283	0.024416238
LOC102723480	uncharacterized LOC102723480	8.053	0.031117076
ANKRD29	ankyrin repeat domain 29	8.048	0.034882773
TMEM255B	transmembrane protein 255B	7.863	0.020426879
ARL4C	ADP ribosylation factor like GTPase 4C	7.489	4.50E-06
UCA1	urothelial cancer associated 1 (non-protein coding)	7.372	1.41E-21
IL1RN	interleukin 1 receptor antagonist	7.002	1.27E-18

NOV	nephroblastoma overexpressed	6.924	0.035766746
CDRT1	CMT1A duplicated region transcript 1	6.649	0.016827859
GALNT5	polypeptide N-acetylgalactosaminyltransferase 5	6.309	0.001648066
ZNF114	zinc finger protein 114	6.302	0.001731296
HDAC9	histone deacetylase 9	6.301	0.000357609
LOC105374003	uncharacterized LOC105374003	6.241	0.006659696
SCEL	sciellin	5.931	0.001828161
IGFL2	IGF like family member 2	5.931	0.003961994
IL6R	interleukin 6 receptor	5.907	1.07E-05
FAM173A	family with sequence similarity 173 member A	5.741	0.02035755
HBEGF	heparin binding EGF like growth factor	5.697	2.68E-08
PTRF	polymerase I and transcript release factor	5.686	6.98E-18
IL32	interleukin 32	5.616	0.02786076
FOSL1	FOS like 1, AP-1 transcription factor subunit	5.532	1.04E-15
GJB3	gap junction protein beta 3	5.489	1.60E-13
AKAP12	A-kinase anchoring protein 12	5.406	0.000157836
SEMA7A	semaphorin 7A (John Milton Hagen blood group)	5.381	0.034775622
IFIT2	interferon induced protein with tetratricopeptide repeats 2	5.236	8.21E-16
LOC107987206	uncharacterized LOC107987206	5.121	6.07E-16
OASL	2'-5'-oligoadenylate synthetase like	4.896	6.66E-14
NTSR1	neurotensin receptor 1	4.872	7.55E-15
MSX2	msh homeobox 2	4.775	0.035687819
RSAD2	radical S-adenosyl methionine domain containing 2	4.771	7.03E-10
TUBB3	tubulin beta 3 class III	4.746	0.003044697
ATP2B4	ATPase plasma membrane Ca2+ transporting 4	4.653	5.82E-14
CDA	cytidine deaminase	4.556	3.97E-11
HLA-B	major histocompatibility complex, class I, B	4.517	0.011509289
CLCF1	cardiotrophin-like cytokine factor 1	4.481	1.24E-12
NLRC5	NLR family CARD domain containing 5	4.464	1.14E-06
SPINK1	serine peptidase inhibitor, Kazal type 1	4.439	0.023427591
NDUFA4L2	NDUFA4, mitochondrial complex associated like 2	4.406	0.000974367
LAMC2	laminin subunit gamma 2	4.402	9.60E-13
AKR1C1	aldo-keto reductase family 1 member C1	4.319	2.09E-11
PMAIP1	phorbol-12-myristate-13-acetate-induced protein 1	4.086	4.23E-05
DPP4	dipeptidyl peptidase 4	4.067	4.23E-10
ACOT7	acyl-CoA thioesterase 7	3.928	0.028748948
HLA-B	major histocompatibility complex, class I, B	3.886	5.78E-05
SFN	stratifin	3.848	2.17E-10
CEACAM6	carcinoembryonic antigen related cell adhesion molecule 6	3.836	2.29E-09
ADAM19	ADAM metallopeptidase domain 19	3.811	6.02E-05
FUT8	fucosyltransferase 8	3.805	0.049833584
LAMA3	laminin subunit alpha 3	3.790	8.90E-11
ELL2	elongation factor for RNA polymerase II 2	3.606	1.57E-09
SPIRE1	spire type actin nucleation factor 1	3.600	9.80E-08
LOC645553	uncharacterized LOC645553	3.599	2.80E-09
EREG	epiregulin	3.567	4.31E-09
SERTAD1	SERTA domain containing 1	3.500	2.30E-08
DUSP10	dual specificity phosphatase 10	3.489	0.008112262
SECTM1	secreted and transmembrane 1	3.489	1.50E-09
MAP2	microtubule associated protein 2	3.481	0.015967901
ANXA1	annexin A1	3.472	9.67E-09
PTGS2	prostaglandin-endoperoxide synthase 2	3.442	2.09E-09
CTSL	cathepsin L	3.441	1.59E-08
KLF4	Kruppel like factor 4	3.399	0.000100998
RHOF	ras homolog family member F, filopodia associated	3.373	6.61E-09
PDLIM7	PDZ and LIM domain 7	3.357	2.49E-06
RHOD	ras homolog family member D	3.348	3.85E-07
NRP1	neuropilin 1	3.329	0.000887425
ISG15	ISG15 ubiquitin-like modifier	3.296	1.29E-08
GLS	glutaminase	3.255	5.99E-08
CDCP1	CUB domain containing protein 1	3.213	6.37E-08
LAT	linker for activation of T-cells	3.197	0.00120829
MLLT11	myeloid/lymphoid or mixed-lineage leukemia; translocated to, 11	3.136	0.0109947
CXCL2	C-X-C motif chemokine ligand 2	3.108	7.23E-05
MID2	midline 2	3.094	0.002403245
ATG4D	autophagy related 4D cysteine peptidase	3.083	0.014317719

SLC25A30	solute carrier family 25 member 30	3.032	0.002810537
PLIN2	perilipin 2	3.024	1.58E-07
FLNA	filamin A	2.928	3.14E-06
ISG20	interferon stimulated exonuclease gene 20	2.920	3.46E-05
P4HA2	prolyl 4-hydroxylase subunit alpha 2	2.887	1.20E-06
GPC1	glypican 1	2.868	1.74E-06
NQO2	NAD(P)H quinone dehydrogenase 2	2.837	0.00196878
CDKN1A	cyclin dependent kinase inhibitor 1A	2.834	1.72E-06
CHAC1	ChaC glutathione specific gamma-glutamylcyclotransferase 1	2.819	0.009631218
SRXN1	sulfiredoxin 1	2.756	7.17E-06
IDS	iduronate 2-sulfatase	2.749	9.90E-06
SDCBP2	syndecan binding protein 2	2.746	3.36E-06
ZSCAN12P1	zinc finger and SCAN domain containing 12 pseudogene 1	2.745	0.000745457
SQRDL	sulfide quinone reductase-like (yeast)	2.742	0.020259647
MFGE8	milk fat globule-EGF factor 8 protein	2.736	0.000208377
TXN	thioredoxin	2.726	1.60E-05
ZNF185	zinc finger protein 185 (LIM domain)	2.707	4.99E-06
WFDC2	WAP four-disulfide core domain 2	2.678	1.40E-05
PLP2	proteolipid protein 2	2.665	1.33E-05
PI3	peptidase inhibitor 3	2.659	2.40E-06
PINK1	PTEN induced putative kinase 1	2.648	6.39E-06
TIMP1	TIMP metallopeptidase inhibitor 1	2.641	1.02E-05
CXCL8	C-X-C motif chemokine ligand 8	2.628	1.33E-05
CDC42EP3	CDC42 effector protein 3	2.627	7.43E-05
CAPN2	calpain 2	2.614	2.75E-05
SLC39A13	solute carrier family 39 member 13	2.611	0.006480976
KLF10	Kruppel like factor 10	2.609	9.66E-06
SPTSSA	serine palmitoyltransferase small subunit A	2.600	0.001035961
TREML2	triggering receptor expressed on myeloid cells like 2	2.592	0.000350856
TNFRSF12A	TNF receptor superfamily member 12A	2.589	1.33E-05
SULT2B1	sulfotransferase family 2B member 1	2.573	1.42E-05
PLAUR	plasminogen activator, urokinase receptor	2.567	1.68E-05
CEACAM5	carcinoembryonic antigen related cell adhesion molecule 5	2.550	2.86E-05
LRP8	LDL receptor related protein 8	2.519	7.67E-05
IMPA2	inositol monophosphatase 2	2.516	7.88E-05
CAPRIN2	caprin family member 2	2.512	6.96E-05
IGFBP6	insulin like growth factor binding protein 6	2.509	0.000228406
LOXL4	lysyl oxidase like 4	2.495	4.63E-05
ABL2	ABL proto-oncogene 2, non-receptor tyrosine kinase	2.475	5.94E-05
LURAP1L	leucine rich adaptor protein 1 like	2.475	9.28E-05
IFIT3	interferon induced protein with tetratricopeptide repeats 3	2.473	5.40E-05
GLRX	glutaredoxin	2.470	0.000658385
EGR1	early growth response 1	2.467	0.000166425
CYP4F11	cytochrome P450 family 4 subfamily F member 11	2.464	0.001320621
CCAT1	colon cancer associated transcript 1 (non-protein coding)	2.435	7.73E-05
ANKRD22	ankyrin repeat domain 22	2.432	0.001845486
LAMB3	laminin subunit beta 3	2.430	0.00025236
SH3KBP1	SH3 domain containing kinase binding protein 1	2.430	0.000107567
PPARD	peroxisome proliferator activated receptor delta	2.417	0.000100998
ALCAM	activated leukocyte cell adhesion molecule	2.414	0.002649636
STBD1	starch binding domain 1	2.411	0.023918262
ST6GALNAC4	ST6 N-acetylgalactosaminide alpha-2,6-sialyltransferase 4	2.410	0.000822164
MOB3C	MOB kinase activator 3C	2.408	6.87E-05
HLA-A	major histocompatibility complex, class I, A	2.404	0.000936675
TXNRD1	thioredoxin reductase 1	2.403	0.000444854
VLDLR	very low density lipoprotein receptor	2.398	0.006318591
COTL1	coactosin like F-actin binding protein 1	2.398	0.000103682
FADS3	fatty acid desaturase 3	2.391	0.003044697
MAP1A	microtubule associated protein 1A	2.389	0.000148154
MXD1	MAX dimerization protein 1	2.388	0.000130541
TMEM265	transmembrane protein 265	2.384	0.003856795
SH3TC2	SH3 domain and tetratricopeptide repeats 2	2.382	0.000134804
ARNTL2	aryl hydrocarbon receptor nuclear translocator like 2	2.361	0.00019065
CPTP	ceramide-1-phosphate transfer protein	2.350	0.005972415
MYEOV	myeloma overexpressed	2.342	0.000280113
IFIT1	interferon induced protein with tetratricopeptide repeats 1	2.337	0.000192761

C6orf223	chromosome 6 open reading frame 223	2.336	0.001106511
RHOB	ras homolog family member B	2.330	0.000223022
SLC27A4	solute carrier family 27 member 4	2.328	0.002868922
PDGFB	platelet derived growth factor subunit B	2.323	0.030133813
PDLIM5	PDZ and LIM domain 5	2.312	0.000627161
HLA-A	major histocompatibility complex, class I, A	2.303	0.000261901
C6orf1	chromosome 6 open reading frame 1	2.300	0.035598022
AP5Z1	adaptor related protein complex 5 zeta 1 subunit	2.281	0.006318591
RNF24	ring finger protein 24	2.275	0.00042326
MICALL1	MICAL like 1	2.275	0.000322409
ECM1	extracellular matrix protein 1	2.264	0.021792663
AKT1S1	AKT1 substrate 1	2.262	0.000658385
BSPRY	B-box and SPRY domain containing	2.250	0.013831217
PPIF	peptidylprolyl isomerase F	2.239	0.000579804
NOSIP	nitric oxide synthase interacting protein	2.236	0.017550578
SLCO4A1	solute carrier organic anion transporter family member 4A1	2.235	0.000627161
KRT80	keratin 80	2.232	0.000616848
NXPE3	neurexophilin and PC-esterase domain family member 3	2.229	0.038725336
LPCAT2	lysophosphatidylcholine acyltransferase 2	2.226	0.000793393
SNORA74A	small nucleolar RNA, H/ACA box 74A	2.220	0.000633005
GCLM	glutamate-cysteine ligase modifier subunit	2.217	0.00110182
JUN	Jun proto-oncogene, AP-1 transcription factor subunit	2.216	0.000936675
ATF3	activating transcription factor 3	2.208	0.003277282
ENO2	enolase 2	2.206	0.004424572
C11orf24	chromosome 11 open reading frame 24	2.203	0.008004439
APRT	adenine phosphoribosyltransferase	2.195	0.006692795
MAPK13	mitogen-activated protein kinase 13	2.190	0.00148469
EPAS1	endothelial PAS domain protein 1	2.186	0.001040986
NOCT	nocturnin	2.185	0.034748331
MGST3	microsomal glutathione S-transferase 3	2.183	0.010221763
SH3BGRL3	SH3 domain binding glutamate rich protein like 3	2.180	0.002297154
TDP2	tyrosyl-DNA phosphodiesterase 2	2.178	0.001060702
ABLIM3	actin binding LIM protein family member 3	2.169	0.001174074
ARL2BP	ADP ribosylation factor like GTPase 2 binding protein	2.166	0.041670299
GPX3	glutathione peroxidase 3	2.158	0.001888852
SLC10A3	solute carrier family 10 member 3	2.152	0.001731296
DHDDS	dehydrodolichyl diphosphate synthase subunit	2.144	0.001731296
CLTB	clathrin light chain B	2.138	0.038567446
OSGIN1	oxidative stress induced growth inhibitor 1	2.130	0.001731296
FTH1P16	ferritin heavy chain 1 pseudogene 16	2.122	0.001610405
ACSL1	acyl-CoA synthetase long-chain family member 1	2.116	0.00258019
CD59	CD59 molecule	2.114	0.004446175
TPRA1	transmembrane protein adipocyte associated 1	2.112	0.00891168
NABP1	nucleic acid binding protein 1	2.111	0.005544972
YRDC	yrdC N6-threonylcarbamoyltransferase domain containing	2.106	0.035880246
RABAC1	Rab acceptor 1	2.102	0.004525921
PKP3	plakophilin 3	2.101	0.002399367
IFITM10	interferon induced transmembrane protein 10	2.091	0.019575473
RHOG	ras homolog family member G	2.089	0.004066989
MYZAP	myocardial zonula adherens protein	2.084	0.005247721
ITPKC	inositol-trisphosphate 3-kinase C	2.080	0.003193015
ITGA3	integrin subunit alpha 3	2.078	0.005972415
MALL	mal, T-cell differentiation protein like	2.070	0.018578373
NCDN	neurochondrin	2.070	0.006525294
TOM1	target of myb1 membrane trafficking protein	2.062	0.020045806
TACSTD2	tumor-associated calcium signal transducer 2	2.050	0.007626992
PODXL	podocalyxin like	2.049	0.008473486
HLA-C	major histocompatibility complex, class I, C	2.048	0.005134135
KLC2	kinesin light chain 2	2.048	0.005090163
GLA	galactosidase alpha	2.046	0.036829644
S100A16	S100 calcium binding protein A16	2.046	0.004110161
ODC1	ornithine decarboxylase 1	2.043	0.005972415
ADAM15	ADAM metallopeptidase domain 15	2.042	0.003853852
CPEB2	cytoplasmic polyadenylation element binding protein 2	2.037	0.018164374
EZR	ezrin	2.020	0.010078286
SRM	spermidine synthase	2.020	0.034533542

GNB5	G protein subunit beta 5	2.020	0.010947358
DCBLD2	discoidin, CUB and LCCL domain containing 2	2.020	0.010947358
SRPRB	SRP receptor beta subunit	2.018	0.005134135
KLHL21	kelch like family member 21	2.018	0.005431255
PRSS3	protease, serine 3	2.016	0.008004439
KCNAB2	potassium voltage-gated channel subfamily A regulatory beta subunit 2	2.012	0.016885691
ASNS	asparagine synthetase (glutamine-hydrolyzing)	2.011	0.005134135
SEC61G	Sec61 translocon gamma subunit	2.011	0.009844088
RAB4B	RAB4B, member RAS oncogene family	2.003	0.038567446
RN7SL4P	RNA, 7SL, cytoplasmic 4, pseudogene	2.002	0.013952378
**Downregulated genes (269)**			
COX2	cytochrome c oxidase subunit II	-2.002	0.01040103
BRWD3	bromodomain and WD repeat domain containing 3	-2.007	0.00503416
MAGED2	MAGE family member D2	-2.010	0.011177354
FRY	FRY microtubule binding protein	-2.018	0.009342067
TNS1	tensin 1	-2.022	0.02452076
SERPINB1	serpin family B member 1	-2.023	0.004815504
ANO9	anoctamin 9	-2.026	0.008193695
ZBTB37	zinc finger and BTB domain containing 37	-2.038	0.020218872
MARCKSL1	MARCKS like 1	-2.038	0.00626795
DPY19L4	dpy-19 like 4 (C. elegans)	-2.043	0.021298522
ARHGAP26	Rho GTPase activating protein 26	-2.049	0.003715828
NFIA	nuclear factor I A	-2.054	0.003235242
SBSPON	somatomedin B and thrombospondin type 1 domain containing	-2.057	0.003596785
SCARNA9	small Cajal body-specific RNA 9	-2.057	0.002868922
DIAPH2	diaphanous related formin 2	-2.060	0.003320834
KLHDC7A	kelch domain containing 7A	-2.061	0.01345768
STK39	serine/threonine kinase 39	-2.062	0.003060387
ZNF827	zinc finger protein 827	-2.067	0.002312942
CPNE2	copine 2	-2.068	0.012142378
GALM	galactose mutarotase	-2.073	0.037042785
MTRNR2L8	MT-RNR2-like 8	-2.073	0.007784735
SLC1A1	solute carrier family 1 member 1	-2.081	0.002257088
FAM13A	family with sequence similarity 13 member A	-2.084	0.043492603
ETS2	ETS proto-oncogene 2, transcription factor	-2.087	0.004424572
LOC107986981	uncharacterized LOC107986981	-2.091	0.023073852
C4orf19	chromosome 4 open reading frame 19	-2.092	0.001859692
VPS13A	vacuolar protein sorting 13 homolog A	-2.096	0.003101956
LOC101060400	uncharacterized LOC101060400	-2.100	0.005609339
TRERF1	transcriptional regulating factor 1	-2.100	0.030259937
TBC1D1	TBC1 domain family member 1	-2.105	0.00503416
LOC105379752	putative uncharacterized protein FLJ46204	-2.108	0.003044697
MAML2	mastermind like transcriptional coactivator 2	-2.111	0.019291377
CORO2A	coronin 2A	-2.112	0.001731296
TCF12	transcription factor 12	-2.115	0.002326437
BCKDHB	branched chain keto acid dehydrogenase E1 subunit beta	-2.118	0.001973714
EMP2	epithelial membrane protein 2	-2.124	0.00135517
FAXDC2	fatty acid hydroxylase domain containing 2	-2.128	0.008283405
RNLS	renalase, FAD dependent amine oxidase	-2.131	0.038396803
TARBP1	TAR (HIV-1) RNA binding protein 1	-2.133	0.001397187
ALDH7A1	aldehyde dehydrogenase 7 family member A1	-2.134	0.026871016
COX1	cytochrome c oxidase subunit I	-2.134	0.001329926
MMS22L	MMS22 like, DNA repair protein	-2.139	0.022235807
PLA2G4A	phospholipase A2 group IVA	-2.143	0.030244159
TMEM168	transmembrane protein 168	-2.143	0.003546792
ABCG2	ATP binding cassette subfamily G member 2 (Junior blood group)	-2.144	0.001040986
ARID5B	AT-rich interaction domain 5B	-2.147	0.001354768
MCAM	melanoma cell adhesion molecule	-2.156	0.007784735
CCSER1	coiled-coil serine rich protein 1	-2.172	0.000992228
ATP6V1B1	ATPase H+ transporting V1 subunit B1	-2.173	0.034093819
UGT1A8	UDP glucuronosyltransferase family 1 member A8	-2.178	0.000799691
ATP10B	ATPase phospholipid transporting 10B (putative)	-2.179	0.001224466
ZNF888	zinc finger protein 888	-2.179	0.000832337
CRYZ	crystallin zeta	-2.180	0.004330053
PRELID2	PRELI domain containing 2	-2.182	0.003585386
SCAPER	S-phase cyclin A associated protein in the ER	-2.189	0.005741624

MAGI1	membrane associated guanylate kinase, WW and PDZ domain containing 1	-2.191	0.005972415
TP53I11	tumor protein p53 inducible protein 11	-2.200	0.004856119
ND6	NADH dehydrogenase, subunit 6 (complex I)	-2.202	0.002608104
ZNF552	zinc finger protein 552	-2.205	0.002248026
GEN1	GEN1, Holliday junction 5' flap endonuclease	-2.211	0.015061117
LY75	lymphocyte antigen 75	-2.214	0.00051051
CABLES1	Cdk5 and Abl enzyme substrate 1	-2.218	0.000945746
YPEL2	yippee like 2	-2.225	0.036559843
MYOM3	myomesin 3	-2.227	0.005972415
CCDC144B	coiled-coil domain containing 144B (pseudogene)	-2.249	0.001094149
NYNRIN	NYN domain and retroviral integrase containing	-2.261	0.000468191
SEMA4G	semaphorin 4G	-2.270	0.000337909
LIPE	lipase E, hormone sensitive type	-2.272	0.000653837
DZIP3	DAZ interacting zinc finger protein 3	-2.279	0.046658045
GLUL	glutamate-ammonia ligase	-2.305	0.000219963
ABCG1	ATP binding cassette subfamily G member 1	-2.331	0.046463911
ARHGAP18	Rho GTPase activating protein 18	-2.335	0.000196072
CALML4	calmodulin like 4	-2.340	0.000150174
CCNG1	cyclin G1	-2.353	0.000116327
ASS1	argininosuccinate synthase 1	-2.357	0.00017951
DBP	D-box binding PAR bZIP transcription factor	-2.372	0.01126228
FOXN3	forkhead box N3	-2.390	0.000416047
ADAM22	ADAM metallopeptidase domain 22	-2.410	0.039059095
HOXB8	homeobox B8	-2.467	3.41E-05
HIST1H1D	histone cluster 1 H1 family member d	-2.490	9.33E-05
DIAPH3	diaphanous related formin 3	-2.494	0.005850117
NECTIN3	nectin cell adhesion molecule 3	-2.500	5.38E-05
SAMD12	sterile alpha motif domain containing 12	-2.526	9.28E-05
HNF4A	hepatocyte nuclear factor 4 alpha	-2.533	0.009631218
TMC5	transmembrane channel like 5	-2.541	2.49E-05
DMTN	dematin actin binding protein	-2.548	0.020560131
TXNIP	thioredoxin interacting protein	-2.551	2.66E-05
ZNF334	zinc finger protein 334	-2.551	0.032535341
LCN2	lipocalin 2	-2.555	1.98E-05
RIN2	Ras and Rab interactor 2	-2.556	0.005676915
MECOM	MDS1 and EVI1 complex locus	-2.558	2.75E-05
ACSS1	acyl-CoA synthetase short-chain family member 1	-2.563	0.018254584
ARPIN	actin-related protein 2/3 complex inhibitor	-2.565	0.00030171
MFSD4A	major facilitator superfamily domain containing 4A	-2.567	0.008221302
MUC13	mucin 13, cell surface associated	-2.575	1.40E-05
HGD	homogentisate 1,2-dioxygenase	-2.576	0.030151155
PRNCR1	prostate cancer associated non-coding RNA 1	-2.579	0.00011628
PRKCA	protein kinase C alpha	-2.580	1.51E-05
FER	FER tyrosine kinase	-2.593	0.001425191
SKP2	S-phase kinase associated protein 2	-2.594	0.00012505
SYBU	syntabulin	-2.597	0.000223022
SYNE2	spectrin repeat containing nuclear envelope protein 2	-2.598	2.67E-05
HOXA5	homeobox A5	-2.609	0.044762099
ANK3	ankyrin 3	-2.612	7.23E-05
PLA2R1	phospholipase A2 receptor 1	-2.615	8.07E-06
CD24	CD24 molecule	-2.623	1.01E-05
DPYSL2	dihydropyrimidinase like 2	-2.629	0.000965269
ANXA9	annexin A9	-2.641	0.006048449
DENND5B	DENN domain containing 5B	-2.645	8.86E-06
NRIP1	nuclear receptor interacting protein 1	-2.653	7.74E-06
ALDH1A1	aldehyde dehydrogenase 1 family member A1	-2.676	3.67E-05
TLR4	toll like receptor 4	-2.679	0.004074744
SIAE	sialic acid acetylesterase	-2.691	0.020708638
TSC22D3	TSC22 domain family member 3	-2.694	5.87E-06
PRSS12	protease, serine 12	-2.705	0.04109117
C1orf21	chromosome 1 open reading frame 21	-2.718	0.012251147
DYNC2H1	dynein cytoplasmic 2 heavy chain 1	-2.727	0.000153055
POF1B	premature ovarian failure, 1B	-2.728	3.17E-06
HOXB3	homeobox B3	-2.728	1.09E-05
PROS1	protein S (alpha)	-2.762	6.28E-05
COBLL1	cordon-bleu WH2 repeat protein like 1	-2.780	1.27E-05

TM4SF4	transmembrane 4 L six family member 4	-2.799	1.99E-05
TEAD2	TEA domain transcription factor 2	-2.802	0.001539242
PRR15L	proline rich 15 like	-2.823	0.013943702
TRIM2	tripartite motif containing 2	-2.828	4.64E-06
LOC105375724	uncharacterized LOC105375724	-2.837	6.68E-07
RBPMS	RNA binding protein with multiple splicing	-2.841	0.036998877
MTA3	metastasis associated 1 family member 3	-2.843	4.46E-05
BHLHE41	basic helix-loop-helix family member e41	-2.909	9.84E-07
ENTPD5	ectonucleoside triphosphate diphosphohydrolase 5	-2.915	2.07E-06
SLC40A1	solute carrier family 40 member 1	-2.932	5.30E-07
ABCC4	ATP binding cassette subfamily C member 4	-2.945	4.24E-07
CREB3L1	cAMP responsive element binding protein 3 like 1	-2.969	2.46E-07
LOC107986513	uncharacterized LOC107986513	-2.980	6.47E-07
AQP5	aquaporin 5	-2.985	0.030244159
MYADM	myeloid associated differentiation marker	-3.007	0.000638586
CACNA1D	calcium voltage-gated channel subunit alpha1 D	-3.017	8.23E-05
VIL1	villin 1	-3.019	1.94E-07
LOC730102	quinone oxidoreductase-like protein 2 pseudogene	-3.028	0.003596785
PTPRJ	protein tyrosine phosphatase, receptor type J	-3.064	7.04E-08
LOC100287290	cytokine receptor CRL2	-3.067	3.33E-05
BCAS1	breast carcinoma amplified sequence 1	-3.098	4.62E-08
SMARCA1	SWI/SNF related, matrix associated, actin dependent regulator of chromatin, subfamily a, member 1	-3.100	4.77E-08
XAF1	XIAP associated factor 1	-3.111	0.001452029
EHHADH	enoyl-CoA hydratase and 3-hydroxyacyl CoA dehydrogenase	-3.119	0.017953289
ATP8	ATP synthase F0 subunit 8	-3.166	1.58E-07
PAPSS2	3'-phosphoadenosine 5'-phosphosulfate synthase 2	-3.198	0.000315393
PLCE1	phospholipase C epsilon 1	-3.239	0.006894681
KIZ	kizuna centrosomal protein	-3.246	6.28E-05
TFPI	tissue factor pathway inhibitor	-3.274	7.99E-09
HCAR1	hydroxycarboxylic acid receptor 1	-3.344	6.56E-06
TSPAN9	tetraspanin 9	-3.365	1.35E-05
LOC102723409	uncharacterized LOC102723409	-3.408	3.88E-06
PDZK1IP1	PDZK1 interacting protein 1	-3.440	0.003341487
CLMN	calmin	-3.459	6.31E-08
KANSL1L	KAT8 regulatory NSL complex subunit 1 like	-3.507	6.17E-08
FGFR2	fibroblast growth factor receptor 2	-3.535	0.011505071
PTPRB	protein tyrosine phosphatase, receptor type B	-3.541	2.20E-07
BCL2L15	BCL2 like 15	-3.546	0.000728345
SPRY1	sprouty RTK signaling antagonist 1	-3.550	0.006659696
USH1C	USH1 protein network component harmonin	-3.646	2.39E-10
MDK	midkine (neurite growth-promoting factor 2)	-3.652	1.21E-10
INSR	insulin receptor	-3.653	0.030242524
IFT80	intraflagellar transport 80	-3.710	1.28E-06
SLC12A2	solute carrier family 12 member 2	-3.726	2.32E-09
SELENBP1	selenium binding protein 1	-3.730	0.000269573
F5	coagulation factor V	-3.733	1.53E-10
PROM1	prominin 1	-3.741	1.50E-08
SCAMP5	secretory carrier membrane protein 5	-3.816	0.023177718
GRAMD1B	GRAM domain containing 1B	-3.857	3.01E-09
MYRFL	myelin regulatory factor-like	-3.871	0.001924602
ADRA2A	adrenoceptor alpha 2A	-3.880	0.034275804
HNF4G	hepatocyte nuclear factor 4 gamma	-3.939	0.000131103
MTRNR2L2	MT-RNR2-like 2	-3.939	2.51E-10
TP53INP1	tumor protein p53 inducible nuclear protein 1	-3.982	0.004330053
NDUFC2-KCTD14	NDUFC2-KCTD14 readthrough	-4.021	0.038850134
SH3PXD2A	SH3 and PX domains 2A	-4.048	5.57E-05
PDZK1	PDZ domain containing 1	-4.141	0.019069817
TSPAN12	tetraspanin 12	-4.158	8.90E-11
CDCA7	cell division cycle associated 7	-4.183	1.56E-10
ALDH6A1	aldehyde dehydrogenase 6 family member A1	-4.263	0.002083909
GDA	guanine deaminase	-4.331	0.00023237
ARHGEF40	Rho guanine nucleotide exchange factor 40	-4.356	0.00503416
TRAF5	TNF receptor associated factor 5	-4.380	1.00E-08
HNF1A	HNF1 homeobox A	-4.532	0.016687695
VWA2	von Willebrand factor A domain containing 2	-4.546	0.000131819

CEACAM7	carcinoembryonic antigen related cell adhesion molecule 7	-4.613	0.006752896
LOC107985962	uncharacterized LOC107985962	-4.664	4.32E-05
ZNF792	zinc finger protein 792	-4.833	0.003104877
IQGAP2	IQ motif containing GTPase activating protein 2	-4.962	3.67E-07
MAP2K6	mitogen-activated protein kinase kinase 6	-4.974	0.023073852
HOXA10-HOXA9	HOXA10-HOXA9 readthrough	-4.989	0.034794494
TPPP3	tubulin polymerization promoting protein family member 3	-5.044	0.000226304
PPP1R1B	protein phosphatase 1 regulatory inhibitor subunit 1B	-5.247	0.007164323
PTK7	protein tyrosine kinase 7 (inactive)	-5.286	0.008739183
PTGS1	prostaglandin-endoperoxide synthase 1	-5.329	2.38E-05
NAALADL2	N-acetylated alpha-linked acidic dipeptidase like 2	-5.526	7.09E-15
F2R	coagulation factor II thrombin receptor	-5.527	4.34E-06
FSIP2	fibrous sheath interacting protein 2	-5.547	0.001081339
CLDN2	claudin 2	-5.571	1.69E-09
LRIG1	leucine rich repeats and immunoglobulin like domains 1	-5.643	0.038751244
AZGP1	alpha-2-glycoprotein 1, zinc-binding	-5.656	0.000436236
MRAS	muscle RAS oncogene homolog	-5.725	3.37E-16
CLRN3	clarin 3	-5.779	0.010553069
FREM2	FRAS1 related extracellular matrix protein 2	-5.902	2.30E-13
MYO7B	myosin VIIB	-5.917	0.003193015
PRLR	prolactin receptor	-6.128	5.52E-10
LOC101927627	uncharacterized LOC101927627	-6.253	0.011321596
DNMT3B	DNA methyltransferase 3 beta	-6.294	0.036862708
LOC107984897	uncharacterized LOC107984897	-6.295	0.017551046
LOC107985977	uncharacterized LOC107985977	-6.362	0.035933985
LOC107985783	uncharacterized LOC107985783	-6.429	0.028183866
PA2G4P6	proliferation-associated 2G4 pseudogene 6	-6.436	0.032981839
IYD	iodotyrosine deiodinase	-6.666	0.026871016
ITGA1	integrin subunit alpha 1	-6.697	0.000147026
STRA6	stimulated by retinoic acid 6	-7.004	0.000189968
CLEC3A	C-type lectin domain family 3 member A	-7.019	8.38E-23
AOC1	amine oxidase, copper containing 1	-7.158	0.030244159
PTPRO	protein tyrosine phosphatase, receptor type O	-7.233	1.83E-08
CFTR	cystic fibrosis transmembrane conductance regulator	-7.414	1.97E-23
NR5A2	nuclear receptor subfamily 5 group A member 2	-7.531	0.014017946
TOX3	TOX high mobility group box family member 3	-7.681	0.017790707
LOC105374285	uncharacterized LOC105374285	-7.740	0.016739932
LOC105370256	uncharacterized LOC105370256	-7.842	1.93E-06
KCNH2	potassium voltage-gated channel subfamily H member 2	-8.033	0.02795979
LOC105375166	uncharacterized LOC105375166	-8.057	0.013750618
ROR1	receptor tyrosine kinase like orphan receptor 1	-8.130	1.49E-11
COLCA1	colorectal cancer associated 1	-8.343	5.25E-05
SLC3A1	solute carrier family 3 member 1	-8.511	0.011159164
PBX1	PBX homeobox 1	-8.519	2.85E-05
DHRS3	dehydrogenase/reductase 3	-8.612	4.61E-09
BANK1	B-cell scaffold protein with ankyrin repeats 1	-8.765	0.005544972
SOSTDC1	sclerostin domain containing 1	-9.013	0.006480976
SNORA81	small nucleolar RNA, H/ACA box 81	-9.250	0.000662791
HNF1A-AS1	HNF1A antisense RNA 1	-9.745	0.000416047
SELL	selectin L	-9.857	0.036559843
CHN2	chimerin 2	-10.090	0.023038258
NR1H4	nuclear receptor subfamily 1 group H member 4	-10.158	0.021298522
LOC107986348	uncharacterized LOC107986348	-10.414	0.018289346
PPP1R14D	protein phosphatase 1 regulatory inhibitor subunit 14D	-10.565	0.045192065
SNORA53	small nucleolar RNA, H/ACA box 53	-10.990	9.49E-16
DNAJB3	DnaJ heat shock protein family (Hsp40) member B3	-11.268	0.002203655
KCNE3	potassium voltage-gated channel subfamily E regulatory subunit 3	-11.618	1.83E-08
LOC107984117	uncharacterized LOC107984117	-11.737	0.008547173
TSPAN8	tetraspanin 8	-11.804	8.38E-28
COLCA2	colorectal cancer associated 2	-12.376	7.87E-05
NEK11	NIMA related kinase 11	-12.473	0.049862387
UPK3A	uroplakin 3A	-13.132	0.003980082
SLITRK6	SLIT and NTRK like family member 6	-14.266	4.21E-10
CERS6-AS1	CERS6 antisense RNA 1	-14.612	0.019864509
NOX1	NADPH oxidase 1	-17.719	9.41E-06
CLCA4	chloride channel accessory 4	-20.665	9.95E-05

PIGR	polymeric immunoglobulin receptor	-24.298	1.92E-13
LGR5	leucine rich repeat containing G protein-coupled receptor 5	-26.321	2.15E-05
RARRES1	retinoic acid receptor responder 1	-29.233	1.71E-31
TCTN1	tectonic family member 1	-45.813	0.031120585
BIVM-ERCC5	BIVM-ERCC5 readthrough	-50.327	1.15E-05
HMGN2P6	high mobility group nucleosomal binding domain 2 pseudogene 6	-53.270	0.046831057
ZNF487	zinc finger protein 487	-57.814	0.027680754
MIA-RAB4B	MIA-RAB4B readthrough (NMD candidate)	-60.015	0.031886731
HPN	hepsin	-62.384	0.02724668
CFL1P3	cofilin 1 pseudogene 3	-63.685	0.009036343
LOC107984730	vesicle-associated membrane protein-associated protein A pseudogene	-64.050	0.00314187
UGT1A1	UDP glucuronosyltransferase family 1 member A1	-64.911	0.002848347
ARPC4-TTLL3	ARPC4-TTLL3 readthrough	-71.115	0.00645244
LOC105373828	uncharacterized LOC105373828	-78.850	0.004192017
RAB4B-EGLN2	RAB4B-EGLN2 readthrough (NMD candidate)	-98.631	0.018980844
CD96	CD96 molecule	-100.928	0.001141202
KLHL23	kelch like family member 23	-168.922	5.04E-07

[1] Slightly different volumes of bacterial inocula were introduced into Flight and Ground cultures (see Methods), which were then allowed to incubate for 6 hr with the host cells. These trends need to be confirmed in future studies.

[2] Significant differences between the Flight and Ground infected cultures were determined according to an FDR < 0.05 and a minimum log2 fold change of 1 or -1 (2-fold increase or decrease in expression, respectively). Log2-fold change values were converted to fold change in this table. Red shading indicates upregulation and blue shading downregulation in the infected Flight culture.

Supplementary Table 9. Bacterial transcripts expressed in infected Flight HT-29 cultures[1]

Gene	Description	Fold Change* (above background)	FDR
talB	transaldolase B	341.966	5.66E-10
dnaK	chaperone protein DnaK	566.752	4.39E-15
dnaJ	chaperone protein DnaJ	86.955	0.008884184
nhaA	Na(+)/H(+) antiporter NhaA	111.313	0.001491851
ileS	isoleucine--tRNA ligase	313.102	1.02E-09
surA	chaperone SurA	137.090	3.63E-05
lpdT	LPS-assembly protein LptD	262.983	3.77E-09
mraW	rRNA small subunit methyltransferase H	91.179	0.016679775
ftsI	peptidoglycan synthase FtsI	144.887	1.29E-05
murE	UDP-N-acetylmuramoylalanyl-D-glutamate--2,6-diaminopimelate ligase	137.184	5.61E-05
murD	UDP-N-acetylmuramoylalanine--D-glutamate ligase	91.266	0.0166552
murC	UDP-N-acetylmuramate--L-alanine ligase	135.862	6.01E-05
ftsA	cell division protein FtsA	115.883	0.00013139
ftsZ	cell division protein FtsZ	222.393	2.45E-08
lpxC	UDP-3-O-[3-hydroxymyristoyl] N-acetylglucosamine deacetylase	510.069	4.24E-12
secA	preprotein translocase subunit SecA	177.223	7.63E-07
guaC	GMP reductase	193.552	1.51E-07
aceE	pyruvate dehydrogenase decarboxylase subunit E1	1592.895	1.47E-43
aceF	pyruvate dehydrogenase dihydrolipoyltransacetylase subunit E2	1062.685	1.22E-25
lpdA	pyruvate dehydrogenase lipoamide dehydrogenase subunit E3	914.682	1.82E-21
acnB	aconitate hydratase B	104.005	0.000508897
yadF	carbonic anhydrase	279.741	2.22E-09
dksA	RNA polymerase-binding transcription factor	136.358	2.15E-05
mrcB	transpeptidase/transglycosylase	107.677	0.000795093
fhuA	ferrichrome outer membranereceptor/ transporter	325.186	6.69E-10
htrA	serine endoprotease	99.393	0.000684486
dapD	2,3,4,5-tetrahydropyridine-2,6-dicarboxylate N-succinyltransferase	169.505	9.45E-07
rpsB	30S ribosomal protein S2	496.756	4.24E-12
tsf	elongation factor Ts	427.435	6.07E-11
frr	ribosome recycling factor	91.854	0.00685563
yaeL	zinc metallopeptidase RseP	177.387	6.57E-07
bamA	outer membrane protein assembly factor BamA	686.069	2.93E-15
STM0225	chaperone protein Skp	168.524	1.96E-06
lpxD	UDP-3-O-[3-hydroxymyristoyl] glucosamine N-acyltransferase	239.145	1.10E-08
lpxA	acyl-[acyl-carrier-protein]--UDP-N-acetylglucosamine O-acyltransferase	132.461	9.45E-05
dnaE	DNA polymerase III subunit alpha	148.707	7.31E-06
accA	acetyl-CoA carboxylase carboxyltransferase subunit alpha	91.521	0.009143141
proS	proline--tRNA ligase	136.276	4.75E-05
mltD	membrane-bound lytic murein transglycosylase D	349.436	4.79E-10
pepD	aminoacyl-histidine dipeptidase	258.750	4.91E-09
foxA	ferrioxamine B receptor	132.379	0.000122485
STM0402	thiol-alkyl hydroperoxide reductase	357.549	3.81E-10
secD	preprotein translocase subunit SecD	164.640	1.04E-06
secF	preprotein translocase subunit SecF	140.921	5.94E-05
yajQ	phage host factor	87.121	0.011460819
cyoB	cytochrome o ubiquinol oxidase subunit I	128.000	2.05E-05
tig	trigger factor	464.979	1.54E-11
clpX	ATP-dependent protease ATP-binding subunit ClpX	313.104	1.49E-09
lon	Lon protease	534.304	1.75E-13
cypD	peptidyl-prolyl cis-trans isomerase	198.525	1.36E-07
ybaY	outer membrane lipoprotein	75.007	0.009029474
acrB	RND family acridine efflux pump	337.083	5.62E-10
acrA	acridine efflux pump	128.066	0.000268248
STM0478	small-conductance mechanosensitive channel	144.231	3.07E-05
htpG	chaperone protein HtpG	431.399	2.10E-11
ppiB	peptidyl-prolyl cis-trans isomerase	140.502	3.74E-05
fepA	outer membrane porin	1069.642	8.68E-25

fes	enterochelin esterase	304.174	1.39E-09
entF	enterobactin synthase subunit F	1139.247	1.05E-27
fepB	ferric-enterobactin ABC transporter substrate-binding protein FepB	82.643	0.01455434
entC	isochorismate synthase	378.947	2.35E-10
entE	2,3-dihydroxybenzoate-AMP ligase	718.990	3.69E-16
entB	2,3-dihydro-2,3-dihydroxybenzoate synthetase	453.288	2.94E-11
entA	2,3-dihydro-2,3-dihydroxybenzoate dehydrogenase	198.026	1.02E-07
ahpC	alkyl hydroperoxide reductase subunit C	267.389	3.94E-09
cspE	RNA chaperone	152.689	4.92E-06
lipA	lipoyl synthase	144.080	1.36E-05
STM0636	hypothetical protein	263.767	3.77E-09
dacA	D-alanyl-D-alanine carboxypeptidase	95.581	0.005682252
leuS	leucine--tRNA ligase	255.387	5.22E-09
glnS	glutamine--tRNA ligase	177.222	1.03E-06
fldA	flavodoxin	95.326	0.013975553
pgm	phosphoglucomutase	185.675	2.78E-07
gltA	citrate synthase	124.580	0.000146232
sucA	2-oxoglutarate dehydrogenase subunit E1	82.735	0.008801829
sucB	2-oxoglutarate dehydrogenase dihydrolipoyltranssuccinase subunit E2	83.145	0.018188223
cydA	cytochrome d terminal oxidase polypeptide subunit I	324.550	6.19E-10
cydB	cytochrome d terminal oxidase polypeptide subunit II	250.981	6.37E-09
tolB	translocation protein TolB	242.949	8.98E-09
pal	peptidoglycan-associated outer membrane lipoprotein	128.152	7.56E-05
aroG	3-deoxy-7-phosphoheptulonate synthase	111.739	0.001451907
gpmA	2,3-bisphosphoglycerate-dependent phosphoglycerate mutase	1416.608	1.37E-35
galM	aldose 1-epimerase	103.766	0.000353839
ybhC	pectinesterase	124.005	0.000443114
uvrB	excinuclease ABC subunit B	103.787	0.002851449
ybhL	permease	91.620	0.022036221
dps	DNA starvation/stationary phase protection protein	87.062	0.000322234
ompX	outer membrane protease	1010.648	2.72E-23
ybjX	VirK-like protein	160.731	3.06E-06
clpA	ATP-dependent Clp protease ATP-binding subunit	272.415	3.09E-09
cydD	cysteine/glutathione ABC transporter permease/ATP-binding protein CycD	116.298	0.000499058
trxB	thioredoxin reductase	177.877	5.73E-07
ftsK	DNA translocase FtsK	242.691	9.24E-09
serS	serine--tRNA ligase	116.125	0.000239994
pflB	pyruvate formate lyase I	1191.943	2.41E-30
serC	3-phosphoserine/phosphohydroxythreonine aminotransferase	144.563	2.41E-05
rpsA	30S ribosomal protein S1	1592.990	1.15E-43
mukB	chromosome partition protein MukB	210.461	4.81E-08
ycbL	metallo-beta-lactamase	132.129	0.000124372
aspC	aspartate aminotransferase	226.792	2.27E-08
ompF	outer membrane protein F	300.566	1.10E-09
asnC	DNA-binding transcriptional regulator AsnC	263.506	3.78E-09
pepN	aminopeptidase N	170.076	1.14E-06
ompA	outer membrane protein A	1636.462	9.54E-45
STM1085	hypothetical protein	132.295	3.89E-05
ycdW	glyoxylate/hydroxypyruvate reductase A	78.739	0.049430321
mdoG	glucan biosynthesis protein G	152.769	8.17E-06
mdoH	gucans biosynthesis glucosyltransferase H	128.232	4.98E-05
grxB	glutaredoxin	119.651	2.94E-05
rne	ribonuclease E	341.232	4.48E-10
STM1190	hypothetical protein	210.378	4.72E-08
fabG	3-oxoacyl-ACP reductase FabG	164.959	9.45E-07
acpP	acyl carrier protein	317.522	8.17E-10
fabF	3-oxoacyl-(acyl carrier protein) synthase II	366.311	3.03E-10
ptsG	PTS system glucose-specific transporter subunit IIBC	205.732	8.55E-08
fhuE	ferric-rhodotorulic acid outer membrane transporter	455.347	2.58E-11
ndh	respiratory NADH dehydrogenase 2	337.084	4.83E-10
ycfJ	outer membrane lipoprotein	111.739	0.001451907
ycfR	outer membrane protein	620.905	9.99E-14
mfd	transcription-repair coupling factor	95.498	0.007651395
phoQ	virulence sensor histidine kinase PhoQ	74.683	0.046007929

phoP	virulence transcriptional regulator PhoP	161.384	3.46E-06
purB	adenylosuccinate lyase	79.087	0.03745205
icdA	isocitrate dehydrogenase	197.695	1.15E-07
STM1254	outer membrane lipoprotein	87.471	0.026274197
mipA	murein-synthesizing holoenzymescaffolding protein	271.536	2.70E-09
gapA	glyceraldehyde-3-phosphate dehydrogenase	3400.016	6.20E-83
sppA	protease 4	112.169	0.001900915
pfkB	6-phosphofructokinase	78.759	0.008835747
thrS	threonine--tRNA ligase	173.084	4.73E-07
infC	translation initiation factor IF-3	645.619	1.52E-14
rplT	50S ribosomal protein L20	296.329	1.59E-09
pheS	phenylalanine--tRNA ligase subunit alpha	119.945	0.000539408
pheT	phenylalanine--tRNA ligase subunit beta	480.438	1.34E-11
ihfA	integration host factor subunit alpha	111.909	0.001441843
STM1345	hypothetical protein	103.701	0.002166443
pps	phosphoenolpyruvate synthase	74.513	0.046422793
ydiJ	oxidase	103.619	0.005250623
sufA	iron-sulfur cluster assembly scaffold protein	235.170	1.39E-08
sufB	cysteine desulfurase activator complex subunit SufB	1345.176	7.11E-34
sufC	ABC transporter ATP-binding protein	686.942	3.36E-15
sufD	cysteine desulfurase activator complex subunit SufD	1110.179	7.78E-27
sufS	bifunctional cysteine desulfurase/selenocysteine lyase	705.370	1.40E-15
ynhA	cysteine desufuration protein SufE	128.069	0.000120721
ynhG	murein L,D-transpeptidase	119.859	0.000726107
lpp	major outer membrane lipoprotein	625.247	2.31E-14
pykF	pyruvate kinase	912.272	1.25E-20
cfa	cyclopropane-fatty-acyl-phospholipid synthase	144.895	2.94E-05
nemA	N-ethylmaleimide reductase	107.246	0.003313037
slyB	outer membrane lipoprotein SlyB	206.316	6.46E-08
tyrS	tyrosine--tRNA ligase	312.110	8.17E-10
tppB	dipeptide/tripeptide transporter permease A	164.791	2.88E-06
rsxC	electron transport complex protein RsxC	86.675	0.036211033
add	adenosine deaminase	107.845	0.00133207
ydgA	periplasmic protein	115.967	0.000508897
manA	mannose-6-phosphate isomerase	152.769	1.55E-05
rstA	response regulator RstA	112.075	0.001058898
ydgH	periplasmic protein	135.941	7.86E-05
pntA	NAD(P) transhydrogenase subunit alpha	374.238	2.42E-10
pntB	NAD(P) transhydrogenase subunit beta	201.922	9.72E-08
sfcA	malate dehydrogenase	99.893	0.003463106
ompD	outer membrane porin protein OmpD	750.848	2.72E-17
STM1586	hypothetical protein	1115.491	1.80E-26
yncB	NADP-dependent oxidoreductase	78.747	0.02287475
tehB	tellurite resistance protein TehB	136.027	6.05E-05
STM1627	alcohol dehydrogenase class III	316.889	6.69E-10
ldhA	D-lactate dehydrogenase	296.776	1.49E-09
fnr	fumarate/nitrate reduction transcriptional regulator	235.255	1.33E-08
ydaA	universal stress protein E	136.520	3.74E-05
tpx	2-Cys peroxiredoxin	75.186	0.027306016
fabI	enoyl-ACP reductase FabI	274.978	2.61E-09
rnb	exoribonuclease II	197.861	1.21E-07
topA	DNA topoisomerase I	132.376	4.75E-05
STM1731	catalase	54.310	0.006608703
tonB	transport protein TonB	116.138	0.000865214
adhE	bifunctional acetaldehyde-CoA/alcohol dehydrogenase	1271.894	4.09E-31
hns	DNA-binding protein H-NS	436.399	4.79E-11
galU	UTP--glucose-1-phosphate uridylyltransferase subunit GalU	312.740	8.82E-10
prsA	ribose-phosphate pyrophosphokinase	206.651	8.14E-08
ychF	ribosome-binding ATPase	95.326	0.010310501
STM1808	cytoplasmic protein	217.826	4.24E-08
minD	ATPase MinD	156.913	6.51E-06
sdaA	L-serine deaminase I/L-threonine deaminase I	124.251	0.000118887
manX	PTS system mannose-specific transporter subunit IIAB	83.237	0.041103978
prc	tail-specific protease	172.995	1.53E-06
proQ	ProP effector	91.005	0.01676257
zwf	glucose-6-phosphate 1-dehydrogenase	198.110	1.38E-07

pykA	pyruvate kinase	136.194	4.75E-05
aspS	aspartate--tRNA ligase	222.308	2.99E-08
otsA	alpha,alpha-trehalose-phosphate synthase	66.647	0.034901782
fliC	flagellin	877.911	2.85E-22
fliD	flagellar hook-associated protein 2	116.125	0.000239994
rcsA	transcriptional regulator RcsA	164.704	4.18E-06
STM2059	hypothetical protein	99.123	0.008801829
hisG	ATP phosphoribosyltransferase	103.358	0.005317227
hisD	histidinol dehydrogenase	144.730	3.77E-05
hisC	histidinol-phosphate aminotransferase	107.422	0.002403211
hisB	bifunctional imidazole glycerol-phosphate dehydratase/histidinol phosphatase	115.799	0.000883894
hisA	1-(5-phosphoribosyl)-5-[(5-phosphoribosylamino)methylideneamino] imidazole-4-carboxamide isomerase	120.542	0.000695851
hisF	imidazole glycerol phosphate synthase subunit HisF	144.234	2.45E-05
wzzB	chain length determinant protein	99.216	0.004787305
udg	UDP-glucose 6-dehydrogenase	119.774	0.000728572
gnd	6-phosphogluconate dehydrogenase	419.607	7.52E-11
rfbK	phosphomannomutase	95.152	0.010361769
rfbM	mannose-1-phosphate guanylyltransferase	107.507	0.002394758
rfbH	lipopolysaccharide biosynthesis protein RfbH	325.095	7.16E-10
rfbG	CDP glucose 4,6-dehydratase	152.769	8.17E-06
rfbF	glucose-1-phosphate cytidylyltransferase	160.894	3.55E-06
galF	UTP--glucose-1-phosphate uridylyltransferase subunit GalF	180.957	4.82E-07
wcaG	GDP-fucose synthetase	115.627	0.001204791
gmd	GDP-D-mannose dehydratase	308.772	8.17E-10
fbaB	fructose-bisphosphate aldolase	103.684	0.000188095
mrp	ATP-binding protein	83.413	0.040755941
metG	methionine--tRNA ligase	144.399	2.43E-05
dld	D-lactate dehydrogenase	108.106	0.002314828
cirA	catecholate siderophore receptor CirA	1353.962	4.04E-35
fruA	PTS system fructose-specific transporter subunit IIBC	346.207	3.85E-10
fruK	1-phosphofructokinase	94.998	0.003463106
fruF	bifunctional PTS system fructose-specific transporter subunit IIA/HPr protein	272.063	3.05E-09
ompC	outer membrane porin protein C	276.390	3.61E-09
gyrA	DNA gyrase subunit A	311.379	1.49E-09
nrdA	ribonucleotide-diphosphate reductase subunit alpha	169.181	1.19E-06
yfbE	UDP-4-amino-4-deoxy-L-arabinose--oxoglutarate aminotransferase	103.445	0.005298763
yfbG	bifunctional UDP-glucuronic acid decarboxylase/UDP-4-amino-4-deoxy-L-arabinose formyltransferase	144.229	3.89E-05
yfbT	phosphatase	91.266	0.0166552
ackA	acetate kinase	624.861	4.38E-14
pta	phosphate acetyltransferase	739.651	6.17E-17
accD	acetyl-CoA carboxylase carboxyltransferase subunit beta	116.205	0.000239879
fabB	3-oxoacyl-ACP synthase	193.881	1.15E-07
aroC	chorismate synthase	99.298	0.008732494
gltX	glutamate--tRNA ligase	103.532	0.003874387
zipA	cell division protein ZipA	95.239	0.01032549
cysK	cysteine synthase A	161.057	3.00E-06
ptsH	PTS system phosphohistidinoprotein-hexose phosphotransferase Hpr	79.346	0.036990212
ptsI	phosphoenolpyruvate-protein phosphotransferase	771.606	7.06E-18
crr	PTS system glucose-specific transporter subunit IIA	238.034	1.12E-08
maeB	malic enzyme	87.386	0.034885393
tktB	transketolase	58.227	0.029903073
nlpB	outer membrane protein assembly factor BamC	177.224	6.70E-07
dapA	4-hydroxy-tetrahydrodipicolinate synthase	197.694	1.41E-07
guaA	GMP synthase	82.177	5.24E-09
guaB	inosine 5'-monophosphate dehydrogenase	522.330	3.06E-12
engA	GTP-binding protein EngA	95.239	0.01032549
bamB	uter membrane protein assembly factor BamB	160.894	3.55E-06
hisS	histidine--tRNA ligase	136.679	2.50E-05
ispG	4-hydroxy-3-methylbut-2-en-1-yl diphosphate synthase (flavodoxin)	124.254	0.000190443
hscA	chaperone protein HscA	144.482	1.58E-05
STM2542	iron-sulfur cluster assembly scaffold protein	95.501	0.013853016

nifS	cysteine desulfurase	390.650	1.52E-10
yfhP	HTH-type transcriptional regulator IscR	345.197	4.27E-10
glyA	serine hydroxymethyltransferase	173.405	4.68E-07
hmpA	bifunctional nitric oxide dioxygenase/dihydropteridine reductase	509.589	5.75E-12
purG	phosphoribosylformylglycinamidine synthase	83.147	0.030839015
lepB	signal peptidase I	91.021	0.004276768
lepA	elongation factor 4	144.239	1.60E-05
rseA	anti-sigma E factor RseA	164.873	2.87E-06
rpoE	ECF RNA polymerase sigma factor RpoE	131.716	9.92E-05
rrlG	23S ribosomal RNA	1349.750	5.63E-148
clpB	chaperone protein ClpB	309.315	1.42E-09
yfiO	outer membrane protein assembly factor BamD	79.000	0.037584526
yfiA	translation inhibitor protein RaiA	144.565	2.35E-06
rplS	50S ribosomal protein L19	94.984	0.007866572
trmD	tRNA (guanine-N(1)-)-methyltransferase	177.958	5.12E-07
rimM	ribosome maturation factor RimM	181.287	3.59E-07
ffh	signal recognition particle protein	95.155	0.007794176
grpE	heat shock protein GrpE	144.565	3.27E-06
iroB	glycosyl transferase	641.177	1.46E-14
iroC	ABC transporter ATP-binding protein	743.803	1.46E-16
iroD	enterochelin esterase-like protein	79.085	0.029019319
iroE	alpha/beta superfamily hydrolase	128.235	0.000265768
iroN	iron-enterobactin outer membrane transporter FepA	2863.691	1.68E-73
virK	VirK-like protein	99.472	0.003550264
mig-14	transcriptional activator	186.091	4.68E-07
ygaU	LysM domain/BON superfamily protein	46.434	0.03186216
stpA	DNA binding protein StpA	107.841	0.000789508
ygaM	inner membrane protein	71.033	0.027171871
nrdI	ribonucleotide reductase stimulatory protein	149.113	1.00E-05
nrdE	ribonucleotide-diphosphate reductase subunit alpha	1100.334	2.74E-25
nrdF	ribonucleotide-diphosphate reductase subunit beta	456.012	2.68E-11
alaS	alanine--tRNA ligase	432.910	5.07E-11
recA	recombinase A	322.124	6.49E-10
sitA	Fur regulated iron ABC transporter substrate-binding protein SitA	231.451	1.75E-08
sitB	Fur regulated iron ABC transporter ATP-binding protein SitB	82.740	0.006944022
sipC	cell invasion protein SipC	74.771	0.028037755
mutS	DNA mismatch repair protein MutS	86.944	0.027018766
rpoS	RNA polymerase sigma factor RpoS	1008.823	3.06E-25
nlpD	Murein hydrolase activator NlpD	907.538	8.46E-21
eno	enolase	2390.934	5.75E-64
pyrG	CTP synthase	346.852	4.68E-10
relA	(P)ppGpp synthetase I	140.665	2.96E-05
STM2963	flavodoxin	119.776	0.000544498
queF	7-cyano-7-deazaguanine reductase	82.974	0.024226675
ygdH	nucleotide binding protein	108.162	0.000607636
ptsP	fused phosphoenolpyruvate-protein phosphotransferase PtsP/GAF domain	87.121	0.011460819
lysS	lysine--tRNA ligase	338.000	6.19E-10
prfB	peptide chain release factor 2	107.265	0.001054219
gcvP	glycine dehydrogenase	238.718	1.10E-08
gcvT	glycine cleavage system aminomethyltransferase T	160.567	3.61E-06
pepP	proline aminopeptidase P II	99.638	0.002663035
rpiA	ribose-5-phosphate isomerase A	91.092	0.016737514
fba	fructose-bisphosphate aldolase	1220.524	1.45E-30
pgk	phosphoglycerate kinase	1034.879	1.99E-24
tktA	transketolase	771.027	6.57E-18
speB	agmatinase	79.259	0.037120907
speA	arginine decarboxylase	91.474	0.000381687
metK	methionine adenosyltransferase	279.388	2.20E-09
yggN	periplasmic protein	132.380	0.000160331
exbB	biopolymer transport protein ExbB	185.759	4.74E-07
parC	DNA topoisomerase IV subunit A	115.884	0.000667121
parE	DNA topoisomerase IV subunit B	107.002	0.001848666
tolC	outer membrane channel protein	214.607	4.23E-08
STM3187	hypothetical protein	103.453	0.001018282
ygiC	glutathionylspermidine synthase	78.660	0.029772505

dnaG	DNA primase	99.307	0.002108061
rpoD	RNA polymerase sigma factor RpoD	309.498	1.59E-09
rnpB	miscRNA	5956.497	1.40E-100
STM3264	hypothetical protein	86.857	0.027059
yraP	periplasmic protein	82.974	0.024226675
deaD	ATP-dependent RNA helicase DeaD	185.349	2.29E-07
nlpI	lipoprotein NlpI	230.853	1.58E-08
pnp	polyribonucleotide nucleotidyltransferas	346.024	4.83E-10
infB	translation initiation factor IF-2	451.863	2.68E-11
nusA	transcription termination/antitermination protein NusA	136.516	1.55E-05
hflB	ATP-dependent metalloprotease	658.460	7.25E-15
rpmA	50S ribosomal protein L27	112.143	0.000384927
rplU	50S ribosomal protein L21	180.881	3.41E-07
murA	UDP-N-acetylglucosamine 1-carboxyvinyltransferase	107.509	0.001788389
rpoN	RNA polymerase factor sigma-54	127.755	5.15E-05
sspA	stringent starvation protein A	128.316	0.000118887
rpsI	30S ribosomal protein S9	214.272	4.26E-08
rplM	50S ribosomal protein L13	259.098	4.87E-09
degQ	serine endoprotease	95.501	0.013853016
mdh	malate dehydrogenase	136.436	1.18E-05
accB	acetyl-CoA carboxylase biotin carboxyl carrier protein subunit	107.260	0.001385671
accC	acetyl-CoA carboxylase biotin carboxylase subunit	369.625	2.35E-10
rrlD	23S ribosomal RNA	3066.503	4.90E-159
rrsD	16S ribosomal RNA	1218.134	8.97E-32
rplQ	50S ribosomal protein L17	250.981	7.26E-09
rpoA	DNA-directed RNA polymerase subunit alpha	237.033	1.09E-26
rpsD	30S ribosomal protein S4	526.961	1.96E-12
rpsK	30S ribosomal protein S11	267.477	3.77E-09
rpsM	30S ribosomal protein S13	131.892	4.90E-05
secY	preprotein translocase subunit SecY	1006.777	8.25E-26
rplO	50S ribosomal protein L15	419.701	7.52E-11
rpsE	30S ribosomal protein S5	308.592	1.57E-09
rplR	50S ribosomal protein L18	99.550	0.001018282
rplF	50S ribosomal protein L6	73.454	1.67E-08
rpsH	30S ribosomal protein S8	152.524	8.25E-06
rpsN	30S ribosomal protein S14	153.009	3.60E-06
rplE	50S ribosomal protein L5	45.020	6.24E-08
rplX	50S ribosomal protein L24	115.718	0.000516519
rplN	50S ribosomal protein L14	23.301	3.11E-05
rpsQ	30S ribosomal protein S17	111.906	0.000814079
rplP	50S ribosomal protein L16	303.455	1.38E-09
rpsC	30S ribosomal protein S3	494.224	2.97E-12
rplV	50S ribosomal protein L22	107.598	0.000406278
rpsS	30S ribosomal protein S19	99.389	0.002100381
rplB	50S ribosomal protein L2	517.987	8.65E-13
rplW	50S ribosomal protein L23	132.135	3.93E-05
rplD	50S ribosomal protein L4	312.287	1.41E-09
rplC	50S ribosomal protein L3	588.557	8.00E-14
rpsJ	30S ribosomal protein S10	103.536	0.000251559
tuf	elongation factor Tu	1309.641	4.78E-35
fusA	elongation factor G	1959.349	1.39E-54
rpsG	30S ribosomal protein S7	267.302	3.77E-09
rpsL	30S ribosomal protein S12	164.715	1.58E-06
fkpA	FKBP-type peptidyl-prolyl cis-trans isomerase	325.643	1.01E-09
slyD	FKBP-type peptidyl-prolyl cis-trans isomerase	103.534	0.001298241
crp	cAMP-activated global transcriptional regulator	82.573	0.00885116
ppiA	peptidyl-prolyl cis-trans isomerase A	86.864	0.01536003
trpS	tryptophan--tRNA ligase	107.762	0.001765289
damX	membrane protein	124.334	0.000148068
aroB	3-dehydroquinate synthase	115.635	0.000518276
yhgF	RNase R	153.095	6.68E-06
feoB	ferrous iron transport protein B	206.648	5.91E-08
yhgI	Fe/S biogenesis protein NfuA	168.775	1.24E-06
glpD	glycerol-3-phosphate dehydrogenase	772.473	3.07E-19
asd	aspartate-semialdehyde dehydrogenase	86.950	0.015304929
rpoH	RNA polymerase sigma factor RpoH	288.301	2.04E-09

ftsY	aignal recognition particle receptor FtsY	107.935	0.002336607
STM3580	inner membrane lipoprotein	107.509	0.001788389
pitA	low-affinity phosphate transporter	103.271	0.005324251
uspA	universal stress protein A	91.187	0.001735388
prlC	oligopeptidase A	148.629	1.83E-06
gor	glutathione oxidoreductase	140.421	1.42E-05
treF	trehalase	62.702	0.006719178
glyS	glycine--tRNA ligase subunit beta	214.104	4.18E-08
glyQ	glycine--tRNA ligase subunit alpha	128.151	0.000201317
STM3663	hypothetical protein	140.584	3.73E-05
mtlA	PTS system mannitol-specific transporter subunit IIABC	127.736	0.000158277
mtlD	mannitol-1-phosphate 5-dehydrogenase	111.901	0.000389915
tdh	L-threonine 3-dehydrogenase	144.401	1.59E-05
kbl	2-amino-3-ketobutyrate CoA ligase	91.521	0.009143141
rfaD	ADP-L-glycero-D-manno-heptose-6-epimerase	108.185	0.001726124
spoT	bifunctional (p)ppGpp synthetase II/ guanosine-3',5'-bis pyrophosphate 3'-pyrophosphohydrolase	115.469	0.000523201
cigR	inner membrane protein	79.526	0.047537052
gyrB	DNA gyrase subunit B	279.917	2.28E-09
yidC	membrane protein insertase YidC	194.212	1.57E-07
glmS	glucosamine--fructose-6-phosphate aminotransferase	210.461	4.94E-08
glmU	bifunctional N-acetylglucosamine-1-phosphate uridyltransferase/glucosamine-1-phosphate acetyltransferase	165.038	1.55E-06
atpD	F0F1 ATP synthase subunit beta	487.977	6.60E-12
atpG	F0F1 ATP synthase subunit gamma	218.921	2.99E-08
atpA	F0F1 ATP synthase subunit alpha	806.172	2.54E-18
atpH	F0F1 ATP synthase subunit delta	78.742	0.038123195
atpF	F0F1 ATP synthase subunit B	152.851	6.68E-06
gidA	tRNA uridine 5-carboxymethylaminomethyl modification protein GidA	140.584	3.73E-05
rrlC	23S ribosomal RNA	18320.494	8.75E-194
STM3898	hypothetical protein	120.112	0.000406278
ilvE	branched-chain amino acid aminotransferase	87.120	0.019961856
ilvC	ketol-acid reductoisomerase	53.447	2.68E-07
rhlB	ATP-dependent RNA helicase RhlB	99.386	0.006385292
trxA	thioredoxin	144.726	1.56E-05
rho	transcription termination factor Rho	325.004	6.69E-10
wzzE	lipopolysaccharide biosynthesis protein WzzE	83.060	0.031556083
wecC	UDP-N-acetyl-D-mannosamine dehydrogenase	91.178	0.022537304
cyaA	adenylate cyclase	316.256	7.98E-10
uvrD	DNA-dependent helicase II	107.336	0.002411705
udp	uridine phosphorylase	144.646	2.40E-05
ubiE	ubiquinone/menaquinone biosynthesis C-methyltransferase UbiE	79.001	0.04883916
ubiB	protein kinase UbiB	87.295	0.026503262
yigC	inner membrane protein; 3-octaprenyl-4-hydroxybenzoate decarboxylase	82.886	0.031834397
pepQ	Xaa-Pro dipeptidase	86.963	0.004273201
rrlA	23S ribosomal RNA	7479.076	9.53E-174
dsbA	thiol:disulfide interchange protein DsbA	95.412	0.007678716
polA	DNA polymerase I	127.900	0.000204432
STM4002	cytoplasmic protein	595.415	1.30E-13
glnA	glutamine synthetase	119.863	0.000318257
typA	GTP-binding elongation factor family protein	365.568	3.38E-10
fdoG	formate dehydrogenase large subunit	78.490	0.029988932
sodA	superoxide dismutase	365.661	3.70E-10
cpxA	sensory kinase CpxS	128.645	0.000116717
cpxP	cpx regulon periplasmic repressor	119.792	7.19E-05
pfkA	6-phosphofructokinase	148.949	8.41E-06
tpiA	triosephosphate isomerase	530.056	1.84E-12
menG	ribonuclease activity regulator protein RraA	112.412	0.001039014
hslU	ATP-dependent protease ATPase subunit HslU	181.773	2.18E-07
STM4118	phosphoethanolamine transferase CptA	148.461	1.56E-05
ppc	phosphoenolpyruvate carboxylase	226.962	2.02E-08
oxyR	DNA-binding transcriptional regulator OxyR	99.556	0.003537433
rrlB	23S ribosomal RNA	4457.818	6.23E-150
tuf	elongation factor Tu	1322.760	1.12E-34

nusG	transcription termination/antitermination protein NusG	91.005	0.01676257
rplK	50S ribosomal protein L11	32.662	0.000188901
rplA	50S ribosomal protein L1	472.519	1.33E-11
rplJ	50S ribosomal protein L10	411.400	9.47E-11
rplL	50S ribosomal protein L7/L12	234.914	1.31E-08
rpoB	DNA-directed RNA polymerase subunit beta	1323.848	1.42E-33
rpoC	DNA-directed RNA polymerase subunit beta'	1579.582	3.60E-41
hupA	DNA-binding protein HU-alpha	128.317	0.00015212
rrlE	23S ribosomal RNA	36296.432	1.34E-139
metH	B12-dependent methionine synthase	74.769	0.045842849
pgi	glucose-6-phosphate isomerase	407.909	1.04E-10
yjbH	outer membrane lipoprotein	95.501	0.013853016
plsB	glycerol-3-phosphate acyltransferase	152.039	7.17E-06
lexA	LexA repressor	103.618	0.003860697
uvrA	excinuclease ABC subunit A	99.388	0.003563157
ssb	single-stranded DNA-binding protein	132.213	7.53E-05
STM4261	inner membrane protein	235.774	1.71E-08
proP	proline/betaine transporter	116.212	0.000386754
aspA	aspartate ammonia-lyase	62.431	0.030839015
groES	co-chaperonin GroES	111.667	9.26E-05
groEL	chaperonin GroEL	1779.354	1.12E-53
efp	elongation factor P	165.119	2.37E-06
miaA	tRNA dimethylallyltransferase	111.308	0.002010453
hfq	RNA-binding protein Hfq	99.051	0.003639612
hflX	GTPase HflX	136.118	2.60E-05
hflK	FtsH protease regulator HflK	218.753	3.25E-08
hflC	FtsH protease regulator HflC	201.922	9.13E-08
purA	adenylosuccinate synthetase	255.560	5.37E-09
vacB	ribonuclease R	235.085	1.36E-08
rpsF	30S ribosomal protein S6	107.251	0.002415638
rplI	50S ribosomal protein L9	148.306	7.50E-06
ytfE	iron-sulfur cluster repair protein	107.847	0.001759067
ytfM	outer membrane protein	115.715	0.000886947
ytfN	periplasmic protein	185.344	4.82E-07
ppa	inorganic pyrophosphatase	246.664	7.53E-09
fbp	fructose-1,6-bisphosphatase	124.334	0.000148068
mpl	UDP-N-acetylmuramate:L-alanyl-gamma-D-glutamyl-meso-diaminopimelate ligase	79.167	0.022367956
valS	valine--tRNA ligase	329.426	6.58E-10
pepA	cytosol aminopeptidase	107.922	0.000786737
yjgP	permease	107.507	0.002394758
yjgQ	permease	91.528	0.016451292
STM4492	cytoplasmic protein	79.089	0.04867233
STM4496	DNA repair ATPase	169.344	1.06E-06
STM4519	NAD-dependent aldehyde dehydrogenase	42.076	0.038336009
mdoB	phosphoglycerol transferase I	111.657	0.000642458
fhuF	ferric hydroximate transport ferric iron reductase	189.738	3.85E-07
osmY	hyperosmotically inducible periplasmic protein	66.645	0.001693119
deoC	2-deoxyribose-5-phosphate aldolase	91.436	0.009176473
deoB	phosphopentomutase	267.740	4.16E-09
deoD	purine nucleoside phosphorylase	152.688	8.18E-06
yjjK	ABC transporter ATP-binding protein	218.333	3.03E-08
arcA	two-component response regulator ArcA	95.327	0.004367867
repA2	DNA replication (plasmid-encoded)	92.167	0.015097344
rlgA	putative integrase protein (plasmid-encoded)	128.070	9.53E-05
PSLT046	putative carbonic anhydrase (plasmid-encoded)	124.258	0.000326829

[1] Fold change indicate expression in the infected Flight cultures above background (relative to uninfected cultures). Significance determined according to an FDR < 0.05. Log2-fold change values were converted to fold change.

Supplementary Table 10. Bacterial transcripts expressed in infected Ground HT-29 cultures[1]

Gene	Description	Fold Change* (above background)	FDR
dnaK	chaperone protein DnaK	259.173	3.77E-09
aceE	pyruvate dehydrogenase decarboxylase subunit E1	215.790	1.11E-11
aceF	pyruvate dehydrogenase dihydrolipoyltransacetylase subunit E2	110.707	1.08E-05
lpdA	pyruvate dehydrogenase lipoamide dehydrogenase subunit E3	102.768	4.47E-05
rpsB	30S ribosomal protein S2	79.529	0.00152656
bamA	outer membrane protein assembly factor BamA	43.233	0.04234548
tig	trigger factor	68.587	0.00433008
clpX	ATP-dependent protease ATP-binding subunit ClpX	83.002	0.00123279
lon	Lon protease	169.552	1.60E-06
htpG	chaperone protein HtpG	154.799	7.43E-06
fepA	outer membrane porin	51.367	0.00983955
entF	enterobactin synthase subunit F	103.653	1.72E-05
entE	2,3-dihydroxybenzoate-AMP ligase	61.799	0.0054494
ahpC	alkyl hydroperoxide reductase subunit C	43.045	0.04677683
gpmA	2,3-bisphosphoglycerate-dependent phosphoglycerate mutase	78.391	0.00017875
dps	DNA starvation/stationary phase protection protein	104.609	0.00025543
ompX	outer membrane protease	58.760	0.0044015
pflB	pyruvate formate lyase I	158.909	3.88E-08
rpsA	30S ribosomal protein S1	223.101	5.30E-12
ompA	outer membrane protein A	212.325	1.27E-11
STM1119	NAD(P)H dehydrogenase	84.732	0.01734099
grxB	glutaredoxin	64.856	0.00710134
gapA	glyceraldehyde-3-phosphate dehydrogenase	29.341	9.28E-18
thrS	threonine--tRNA ligase	46.743	0.0328973
infC	translation initiation factor IF-3	50.584	0.01991171
sufB	cysteine desulfurase activator complex subunit SufB	100.646	1.27E-05
sufD	cysteine desulfurase activator complex subunit SufD	103.540	1.95E-05
lpp	major outer membrane lipoprotein	72.561	0.00220372
pykF	pyruvate kinase	54.819	0.00827378
STM1513	cytoplasmic protein	200.975	6.33E-07
ompD	outer membrane porin protein OmpD	94.826	0.00018353
STM1586	hypothetical protein	73.936	0.00056052
yciE	cytoplasmic protein	80.782	0.03979437
STM1731	catalase	194.030	6.42E-07
adhE	bifunctional acetaldehyde-CoA/alcohol dehydrogenase	74.320	0.00039074
fliC	flagellin	211.274	3.86E-09
fbaB	fructose-bisphosphate aldolase	57.332	0.01843163
cirA	catecholate siderophore receptor CirA	152.389	3.37E-08
ompC	outer membrane porin protein C	61.352	0.00767944
gyrA	DNA gyrase subunit A	75.791	0.00221468
ackA	acetate kinase	43.144	0.04551514
pta	phosphate acetyltransferase	87.497	0.00038578
fabB	3-oxoacyl-ACP synthase	46.774	0.03190145
ptsI	phosphoenolpyruvate-protein phosphotransferase	102.185	8.31E-05
glyA	serine hydroxymethyltransferase	46.744	0.0328973
rrlG	23S ribosomal RNA	30.158	2.43E-52
clpB	chaperone protein ClpB	225.633	2.42E-07
yfiA	translation inhibitor protein RaiA	101.268	0.00022094
grpE	heat shock protein GrpE	65.018	0.00574178
iroB	glycosyl transferase	61.622	0.00642321
iroC	ABC transporter ATP-binding protein	50.764	0.0169945
iroN	iron-enterobactin outer membrane transporter FepA	162.958	2.27E-10
ygaU	LysM domain/BON superfamily protein	103.945	0.00069547
alaS	alanine--tRNA ligase	46.634	0.03582473
sitA	Fur regulated iron ABC transporter substrate-binding protein SitA	65.066	0.00510779
rpoS	RNA polymerase sigma factor RpoS	172.403	3.49E-08
nlpD	Murein hydrolase activator NlpD	73.359	0.00099118
eno	enolase	177.415	7.09E-11
fba	fructose-bisphosphate aldolase	126.071	1.10E-06

pgk	phosphoglycerate kinase	84.830	0.00019867
tktA	transketolase	105.819	6.00E-05
speA	arginine decarboxylase	75.516	0.00363269
rpoD	RNA polymerase sigma factor RpoD	79.403	0.00165141
rnpB	miscRNA	1051.758	6.70E-45
infB	translation initiation factor IF-2	53.986	0.01711276
hflB	ATP-dependent metalloprotease	57.983	0.00889671
rrlD	23S ribosomal RNA	57.033	1.62E-65
rrsD	16S ribosomal RNA	7.622	0.0005445
rpoA	DNA-directed RNA polymerase subunit alpha	13.496	1.59E-05
rpsD	30S ribosomal protein S4	54.073	0.0160496
secY	preprotein translocase subunit SecY	39.312	9.28E-08
rplF	50S ribosomal protein L6	50.311	0.02413302
rplB	50S ribosomal protein L2	12.521	0.00796151
rplC	50S ribosomal protein L3	90.634	0.00046371
tuf	elongation factor Tu	41.139	4.22E-09
fusA	elongation factor G	272.067	4.14E-15
fkpA	FKBP-type peptidyl-prolyl cis-trans isomerase	72.161	0.00312084
glpD	glycerol-3-phosphate dehydrogenase	196.118	3.39E-08
prlC	oligopeptidase A	90.460	0.00053022
treF	trehalase	85.994	0.00245147
atpA	F0F1 ATP synthase subunit alpha	76.737	0.00090433
rrlC	23S ribosomal RNA	79.605	4.85E-76
ilvC	ketol-acid reductoisomerase	50.423	0.02154135
rrlA	23S ribosomal RNA	80.685	4.45E-74
STM4002	cytoplasmic protein	50.500	0.02144679
typA	GTP-binding elongation factor family protein	46.632	0.03535343
sodA	superoxide dismutase	82.975	0.00129205
tpiA	triosephosphate isomerase	50.403	0.02323298
hslU	ATP-dependent protease ATPase subunit HslU	79.628	0.00127907
rrlB	23S ribosomal RNA	91.438	3.51E-71
tuf	elongation factor Tu	177.880	2.72E-09
rplA	50S ribosomal protein L1	57.664	0.01176427
rplJ	50S ribosomal protein L10	46.627	0.03582473
rpoB	DNA-directed RNA polymerase subunit beta	7.609	0.00901243
rpoC	DNA-directed RNA polymerase subunit beta'	105.009	4.16E-06
rrlE	23S ribosomal RNA	64.862	9.36E-58
pgi	glucose-6-phosphate isomerase	46.626	0.03582473
STM4261	inner membrane protein	83.148	0.00102942
groES	co-chaperonin GroES	53.747	0.02396165
groEL	chaperonin GroEL	108.096	4.77E-24
vacB	ribonuclease R	46.767	0.03150487
STM4519	NAD-dependent aldehyde dehydrogenase	133.018	5.10E-05
osmY	hyperosmotically inducible periplasmic protein	172.709	2.01E-06
deoB	phosphopentomutase	50.406	0.02183121
yjjK	ABC transporter ATP-binding protein	57.791	0.00984699

[1] Fold change indicates expression in the infected Ground cultures above background (relative to uninfected ground cultures/no bacteria). Significance determined according to an FDR < 0.05. Log2-fold change values were converted to fold change.

Supplementary Table 11. Differential expression of bacterial transcripts between infected Flight and infected Ground HT-29 cultures[1]

Gene	Description	Fold Change (Flight/Ground)	FDR
**Upregulated genes (328)**			
STM1627	alcohol dehydrogenase class III	316.889	2.80E-09
gmd	GDP-D-mannose dehydratase	308.772	3.48E-09
STM1808	cytoplasmic protein	217.826	1.82E-07
fhuF	ferric hydroximate transport ferric iron reductase	189.738	1.39E-06
rcsA	transcriptional regulator RcsA	164.704	1.18E-05
manA	mannose-6-phosphate isomerase	152.769	3.66E-05
hisD	histidinol dehydrogenase	144.730	8.14E-05
yfbG	bifunctional UDP-glucuronic acid decarboxylase/UDP-4-amino-4-deoxy-L-arabinose formyltransferase	144.229	8.37E-05
secF	preprotein translocase subunit SecF	140.921	0.000120443
yggN	periplasmic protein	132.380	0.000295131
iroE	alpha/beta superfamily hydrolase	128.235	0.000460929
acrA	acridine efflux pump	128.066	0.000465064
ybhC	pectinesterase	124.005	0.000722328
hisA	1-(5-phosphoribosyl)-5-[(5-phosphoribosylamino)methylideneamino] imidazole-4-carboxamide isomerase	120.542	0.001055764
ynhG	murein L,D-transpeptidase	119.859	0.001092805
udg	UDP-glucose 6-dehydrogenase	119.774	0.001094149
wcaG	GDP-fucose synthetase	115.627	0.001727127
sppA	protease 4	112.169	0.00257034
miaA	tRNA dimethylallyltransferase	111.308	0.002681966
nemA	N-ethylmaleimide reductase	107.246	0.004053641
ydiJ	oxidase	103.619	0.006004629
yfbE	UDP-4-amino-4-deoxy-L-arabinose--oxoglutarate aminotransferase	103.445	0.006050678
hisG	ATP phosphoribosyltransferase	103.358	0.006073851
pitA	low-affinity phosphate transporter	103.271	0.006097123
aroC	chorismate synthase	99.298	0.009185762
STM2059	hypothetical protein	99.123	0.00927041
degQ	serine endoprotease	95.501	0.013655347
STM2542	iron-sulfur cluster assembly scaffold protein	95.501	0.013655347
yjbH	outer membrane lipoprotein	95.501	0.013655347
fldA	flavodoxin	95.326	0.013750618
ybhL	permease	91.620	0.020045806
wecC	UDP-N-acetyl-D-mannosamine dehydrogenase	91.178	0.02036155
maeB	malic enzyme	87.386	0.029727132
rsxC	electron transport complex protein RsxC	86.675	0.030546195
spr	outer membrane lipoprotein	83.418	0.042935119
yebG	DNA damage-inducible protein	83.418	0.042935119
yibN	rhodanese-like sulfurtransferase	83.329	0.042969066
yjeI	outer membrane lipoprotein	83.329	0.042969066
rna	RNase I	83.239	0.043127763
mltC	lytic murein transglycosylase C	83.150	0.04324679
sfbA	ABC transporter substrate-binding protein	82.882	0.043650908
wcaI	glycosyl transferase	82.793	0.04381618
ycfR	outer membrane protein	69.254	1.86E-10
hmpA	bifunctional nitric oxide dioxygenase/dihydropteridine reductase	56.905	6.61E-09
mipA	murein-synthesizing holoenzymescaffolding protein	54.184	3.67E-07
sufA	iron-sulfur cluster assembly scaffold protein	46.927	1.74E-06
aspC	aspartate aminotransferase	45.257	2.67E-06
aspS	aspartate--tRNA ligase	44.364	3.36E-06
accC	acetyl-CoA carboxylase biotin carboxylase subunit	41.277	2.20E-07
fruA	PTS system fructose-specific transporter subunit IIBC	38.652	3.67E-07
mig-14	transcriptional activator	37.162	3.46E-05
exbB	biopolymer transport protein ExbB	37.096	3.50E-05
ytfN	periplasmic protein	37.014	3.58E-05
cydA	cytochrome d terminal oxidase polypeptide subunit I	36.226	6.06E-07

recA	recombinase A	35.954	6.40E-07
glnS	glutamine--tRNA ligase	35.402	6.59E-05
tyrS	tyrosine--tRNA ligase	34.832	8.32E-07
prc	tail-specific protease	34.564	9.10E-05
nrdE	ribonucleotide-diphosphate reductase subunit alpha	33.951	3.13E-16
ompF	outer membrane protein F	33.539	1.14E-06
lpdT	LPS-assembly protein LptD	29.336	3.68E-06
cfa	cyclopropane-fatty-acyl-phospholipid synthase	29.004	0.001018088
STM0478	small-conductance mechanosensitive channel	28.871	0.001051117
ydgH	periplasmic protein	27.234	0.00223224
murC	UDP-N-acetylmuramate--L-alanine ligase	27.207	0.001828161
rne	ribonuclease E	26.544	2.01E-06
foxA	ferrioxamine B receptor	26.533	0.003126758
ycbL	metallo-beta-lactamase	26.483	0.003166296
ndh	respiratory NADH dehydrogenase 2	26.219	2.20E-06
glyQ	glycine--tRNA ligase subunit alpha	25.699	0.004533637
polA	DNA polymerase I	25.649	0.00459994
PSLT046		24.933	0.00645244
pheS	phenylalanine--tRNA ligase subunit alpha	24.083	0.009477749
STM2963	flavodoxin	24.049	0.009565561
pheT	phenylalanine--tRNA ligase subunit beta	23.408	9.00E-07
nifS	cysteine desulfurase	23.390	2.51E-06
tonB	transport protein TonB	23.335	0.013655347
hisB	bifunctional imidazole glycerol-phosphate dehydratase/histidinol phosphatase	23.267	0.013807237
ytfM	outer membrane protein	23.250	0.013843091
prsA	ribose-phosphate pyrophosphokinase	23.062	5.06E-05
ptsG	PTS system glucose-specific transporter subunit IIBC	22.959	5.27E-05
ihfA	integration host factor subunit alpha	22.503	0.019725055
aroG	3-deoxy-7-phosphoheptulonate synthase	22.469	0.019858283
ycfJ	outer membrane lipoprotein	22.469	0.019858283
nhaA	Na(+)/H(+) antiporter NhaA	22.384	0.020233506
dld	D-lactate dehydrogenase	21.756	0.02795979
ftsY	aignal recognition particle receptor FtsY	21.722	0.028063791
rfbM	mannose-1-phosphate guanylyltransferase	21.637	0.028666
yjgP	permease	21.637	0.028666
hisC	histidinol-phosphate aminotransferase	21.619	0.028748948
uvrD	DNA-dependent helicase II	21.602	0.028864801
rpsF	30S ribosomal protein S6	21.585	0.028981133
lexA	LexA repressor	20.873	0.040481006
gltX	glutamate--tRNA ligase	20.856	0.040560398
fepA	outer membrane porin	20.823	1.73E-13
yfhP	HTH-type transcriptional regulator IscR	20.657	6.70E-06
STM0636	hypothetical protein	20.496	1.61E-05
sufS	bifunctional cysteine desulfurase/selenocysteine lyase	19.712	2.76E-08
sufC	ABC transporter ATP-binding protein	19.209	5.46E-08
cyaA	adenylate cyclase	18.915	1.30E-05
STM0225	chaperone protein Skp	18.841	0.000627161
entB	2,3-dihydro-2,3-dihydroxybenzoate synthetase	18.646	4.64E-06
lpxD	UDP-3-O-[3-hydroxymyristoyl] glucosamine N-acyltransferase	18.581	4.08E-05
rseA	anti-sigma E factor RseA	18.439	0.000818737
tppB	dipeptide/tripeptide transporter permease A	18.430	0.000820857
fnr	fumarate/nitrate reduction transcriptional regulator	18.279	4.85E-05
gpmA	2,3-bisphosphoglycerate-dependent phosphoglycerate mutase	18.071	1.11E-18
iroN	iron-enterobactin outer membrane transporter FepA	17.573	1.37E-37
minD	ATPase MinD	17.562	0.00148469
ompX	outer membrane protease	17.200	1.27E-11
adhE	bifunctional acetaldehyde-CoA/alcohol dehydrogenase	17.114	8.36E-16
rplK	50S ribosomal protein L11	17.090	0.001809281
metK	methionine adenosyltransferase	16.699	3.41E-05
pykF	pyruvate kinase	16.641	3.97E-10
STM4118	phosphoethanolamine transferase CptA	16.632	0.002887509
guaB	inosine 5'-monophosphate dehydrogenase	16.396	4.03E-06
nrdF	ribonucleotide-diphosphate reductase subunit beta	16.241	1.03E-05
udp	uridine phosphorylase	16.214	0.003896256
serC	3-phosphoserine/phosphohydroxythreonine aminotransferase	16.204	0.003909336

metG	methionine--tRNA ligase	16.186	0.003942652
hisF	imidazole glycerol phosphate synthase subunit HisF	16.168	0.003969222
guaA	GMP synthase	15.899	2.14E-05
bamA	outer membrane protein assembly factor BamA	15.869	2.36E-07
fhuA	ferrichrome outer membranereceptor/ transporter	15.831	3.09E-05
rho	transcription termination factor Rho	15.823	3.09E-05
gidA	tRNA uridine 5-carboxymethylaminomethyl modification protein GidA	15.768	0.005382275
STM3663	hypothetical protein	15.768	0.005382275
ppiB	peptidyl-prolyl cis-trans isomerase	15.759	0.005401003
asnC	DNA-binding transcriptional regulator AsnC	15.747	5.56E-05
cypD	peptidyl-prolyl cis-trans isomerase	15.437	0.00030171
zwf	glucose-6-phosphate 1-dehydrogenase	15.404	0.000307488
murE	UDP-N-acetylmuramoylalanyl-D-glutamate--2,6-diaminopimelate ligase	15.396	0.007164323
dapA	4-hydroxy-tetrahydrodipicolinate synthase	15.372	0.000313381
tehB	tellurite resistance protein TehB	15.267	0.007626992
leuS	leucine--tRNA ligase	15.260	7.23E-05
galU	UTP--glucose-1-phosphate uridylyltransferase subunit GalU	15.222	4.08E-05
STM1586	hypothetical protein	15.087	1.46E-12
rpoC	DNA-directed RNA polymerase subunit beta'	15.042	5.11E-20
lpxA	acyl-[acyl-carrier-protein]--UDP-N-acetylglucosamine O-acyltransferase	14.877	0.010314657
rpoE	ECF RNA polymerase sigma factor RpoE	14.794	0.010677866
ppa	inorganic pyrophosphatase	14.738	9.80E-05
iroC	ABC transporter ATP-binding protein	14.652	1.19E-07
tolB	translocation protein TolB	14.516	0.000112525
ackA	acetate kinase	14.483	1.67E-06
hupA	DNA-binding protein HU-alpha	14.424	0.014401528
mtlA	PTS system mannitol-specific transporter subunit IIABC	14.359	0.014845065
yaeL	zinc metallopeptidase RseP	13.805	0.001001521
secA	preprotein translocase subunit SecA	13.796	0.001087146
hns	DNA-binding protein H-NS	13.715	3.43E-05
gyrB	DNA gyrase subunit B	13.616	9.63E-05
yadF	carbonic anhydrase	13.607	9.50E-05
ppc	phosphoenolpyruvate carboxylase	13.562	0.000219963
STM3898	hypothetical protein	13.526	0.02786076
eno	enolase	13.477	2.31E-29
fabI	enoyl-ACP reductase FabI	13.374	0.000108154
sufB	cysteine desulfurase activator complex subunit SufB	13.365	2.28E-15
pntA	NAD(P) transhydrogenase subunit alpha	13.328	6.28E-05
hflK	FtsH protease regulator HflK	13.073	0.000318304
parC	DNA topoisomerase IV subunit A	13.064	0.038567446
acpP	acyl carrier protein	13.046	9.26E-05
lpxC	UDP-3-O-[3-hydroxymyristoyl] N-acetylglucosamine deacetylase	12.979	2.14E-05
efp	elongation factor P	12.868	0.002459859
tolC	outer membrane channel protein	12.826	0.000391152
rpsI	30S ribosomal protein S9	12.806	0.000396286
infC	translation initiation factor IF-3	12.763	2.82E-06
rpoB	DNA-directed RNA polymerase subunit beta	12.688	7.74E-15
phoP	virulence transcriptional regulator PhoP	12.582	0.003184597
bamB	uter membrane protein assembly factor BamB	12.544	0.003235242
rfbF	glucose-1-phosphate cytidylyltransferase	12.544	0.003235242
gcvT	glycine cleavage system aminomethyltransferase T	12.519	0.003285715
nlpD	Murein hydrolase activator NlpD	12.371	8.57E-09
pgk	phosphoglycerate kinase	12.199	2.71E-10
pntB	NAD(P) transhydrogenase subunit beta	12.072	0.000737533
acrB	RND family acridine efflux pump	11.998	0.000135445
mdoG	glucan biosynthesis protein G	11.923	0.005727013
rfbG	CDP glucose 4,6-dehydratase	11.923	0.005727013
atpF	F0F1 ATP synthase subunit B	11.923	0.005090163
deoD	purine nucleoside phosphorylase	11.917	0.005741624
rpsH	30S ribosomal protein S8	11.904	0.005782207
gnd	6-phosphogluconate dehydrogenase	11.806	9.63E-05
STM4002	cytoplasmic protein	11.790	1.20E-05
valS	valine--tRNA ligase	11.723	0.00015982

entE	2,3-dihydroxybenzoate-AMP ligase	11.634	1.40E-06
rfbH	lipopolysaccharide biosynthesis protein RfbH	11.568	0.000177638
fabF	3-oxoacyl-(acyl carrier protein) synthase II	11.510	0.000153222
hflB	ATP-dependent metalloprotease	11.356	5.39E-06
relA	(P)ppGpp synthetase I	11.000	0.013655347
entF	enterobactin synthase subunit F	10.991	4.43E-11
pyrG	CTP synthase	10.895	0.000232618
tsf	elongation factor Ts	10.889	0.000149595
galF	UTP--glucose-1-phosphate uridylyltransferase subunit GalF	10.830	0.002302554
sufD	cysteine desulfurase activator complex subunit SufD	10.722	1.52E-10
proS	proline--tRNA ligase	10.665	0.018687396
pykA	pyruvate kinase	10.659	0.018754493
nlpB	outer membrane protein assembly factor BamC	10.610	0.002887509
fhuE	ferric-rhodotorulic acid outer membrane transporter	10.599	0.000139218
tpiA	triosephosphate isomerase	10.516	6.59E-05
atpA	F0F1 ATP synthase subunit alpha	10.506	5.30E-07
glyS	glycine--tRNA ligase subunit beta	10.411	0.001022793
iroB	glycosyl transferase	10.405	1.40E-05
ssb	single-stranded DNA-binding protein	10.356	0.024775592
rrlG	23S ribosomal RNA	10.339	3.06E-28
cydB	cytochrome d terminal oxidase polypeptide subunit II	10.299	0.000594873
glmS	glucosamine--fructose-6-phosphate aminotransferase	10.234	0.0011649
nrdA	ribonucleotide-diphosphate reductase subunit alpha	10.132	0.0042773
cpxA	sensory kinase CpxS	10.085	0.032631898
sspA	stringent starvation protein A	10.060	0.033121656
STM0402	thiol-alkyl hydroperoxide reductase	10.057	0.000370334
ynhA	cysteine desufuration protein SufE	10.041	0.03356713
hflC	FtsH protease regulator HflC	9.822	0.001797188
rplO	50S ribosomal protein L15	9.773	0.000317817
crr	PTS system glucose-specific transporter subunit IIA	9.766	0.000916026
ispG	4-hydroxy-3-methylbut-2-en-1-yl diphosphate synthase (flavodoxin)	9.751	0.044081936
rpsD	30S ribosomal protein S4	9.745	0.000112525
pnp	polyribonucleotide nucleotidyltransferas	9.730	0.000474905
fba	fructose-bisphosphate aldolase	9.681	2.35E-11
fruF	bifunctional PTS system fructose-specific transporter subunit IIA/HPr protein	9.669	0.000681852
cysK	cysteine synthase A	9.658	0.007629004
ybjX	VirK-like protein	9.638	0.007733603
rplL	50S ribosomal protein L7/L12	9.638	0.001018088
rnb	exoribonuclease II	9.626	0.002203655
aceF	pyruvate dehydrogenase dihydrolipoyltransacetylase subunit E2	9.599	2.27E-09
yidC	membrane protein insertase YidC	9.450	0.002649636
guaC	GMP reductase	9.416	0.002614779
ldhA	D-lactate dehydrogenase	9.312	0.000737533
alaS	alanine--tRNA ligase	9.283	0.00040408
rrlD	23S ribosomal RNA	9.234	1.55E-25
yhgF	RNase R	9.190	0.012773942
plsB	glycerol-3-phosphate acyltransferase	9.127	0.013482196
purA	adenylosuccinate synthetase	9.080	0.001087146
nrdI	ribonucleotide reductase stimulatory protein	8.957	0.016667669
lpdA	pyruvate dehydrogenase lipoamide dehydrogenase subunit E3	8.900	2.27E-07
cirA	catecholate siderophore receptor CirA	8.885	1.65E-12
entC	isochorismate synthase	8.824	0.000774627
rplJ	50S ribosomal protein L10	8.823	0.000658385
ileS	isoleucine--tRNA ligase	8.798	0.000971633
rrlC	23S ribosomal RNA	8.752	4.88E-25
pgi	glucose-6-phosphate isomerase	8.748	0.000704738
trxA	thioredoxin	8.699	0.021752131
hscA	chaperone protein HscA	8.685	0.022001935
tdh	L-threonine 3-dehydrogenase	8.680	0.022076981
lepA	elongation factor 4	8.670	0.022227912
trxB	thioredoxin reductase	8.664	0.006105287
lpp	major outer membrane lipoprotein	8.617	7.23E-05
gapA	glyceraldehyde-3-phosphate dehydrogenase	8.544	1.13E-27
clpA	ATP-dependent Clp protease ATP-binding subunit	8.542	0.001354768
rplP	50S ribosomal protein L16	8.526	0.00121169

slyB	outer membrane lipoprotein SlyB	8.467	0.003193015
pta	phosphate acetyltransferase	8.453	1.33E-05
infB	translation initiation factor IF-2	8.370	0.00068094
rrlA	23S ribosomal RNA	8.300	1.78E-23
pepN	aminopeptidase N	8.290	0.009462405
surA	chaperone SurA	8.253	0.036263219
yhgI	Fe/S biogenesis protein NfuA	8.227	0.010017335
ydaA	universal stress protein E	8.219	0.037095035
rplA	50S ribosomal protein L1	8.194	0.000658385
mltD	membrane-bound lytic murein transglycosylase D	8.134	0.001397187
rplM	50S ribosomal protein L13	8.122	0.00196878
icdA	isocitrate dehydrogenase	8.116	0.004685088
pepD	aminoacyl-histidine dipeptidase	8.112	0.001984094
rplN	50S ribosomal protein L14	7.975	0.005209193
talB	transaldolase B	7.959	0.001647903
ompD	outer membrane porin protein OmpD	7.918	1.91E-05
ftsZ	cell division protein FtsZ	7.900	0.003341487
lysS	lysine--tRNA ligase	7.866	0.001777858
typA	GTP-binding elongation factor family protein	7.839	0.001687643
atpG	F0F1 ATP synthase subunit gamma	7.776	0.003853852
fes	enterochelin esterase	7.739	0.002191765
ompA	outer membrane protein A	7.707	1.33E-14
rrlE	23S ribosomal RNA	7.627	4.75E-22
pgm	phosphoglucomutase	7.627	0.008214252
ftsK	DNA translocase FtsK	7.606	0.003223793
ptsI	phosphoenolpyruvate-protein phosphotransferase	7.551	1.99E-05
rplT	50S ribosomal protein L20	7.537	0.00257034
atpD	F0F1 ATP synthase subunit beta	7.509	0.000974367
rpsK	30S ribosomal protein S11	7.508	0.002947417
rpsG	30S ribosomal protein S7	7.503	0.002958376
pflB	pyruvate formate lyase I	7.501	2.12E-09
gcvP	glycine dehydrogenase	7.481	0.003660807
mukB	chromosome partition protein MukB	7.477	0.00535239
tuf	elongation factor Tu	7.436	8.90E-11
aceE	pyruvate dehydrogenase decarboxylase subunit E1	7.382	1.05E-13
rpoH	RNA polymerase sigma factor RpoH	7.332	0.003088597
trmD	tRNA (guanine-N(1)-)-methyltransferase	7.314	0.012127108
tktA	transketolase	7.286	2.74E-05
pfkA	6-phosphofructokinase	7.280	0.03193175
nlpI	lipoprotein NlpI	7.234	0.00475342
fusA	elongation factor G	7.202	6.37E-17
rrsD	16S ribosomal RNA	7.160	1.48E-09
rpsA	30S ribosomal protein S1	7.140	2.08E-13
ftsI	peptidoglycan synthase FtsI	7.086	0.040560398
rrlB	23S ribosomal RNA	7.085	5.19E-20
lipA	lipoyl synthase	7.047	0.042192797
STM4496	DNA repair ATPase	6.965	0.01887863
rplE	50S ribosomal protein L5	6.955	0.00338606
rplF	50S ribosomal protein L6	6.867	0.004011835
glmU	bifunctional N-acetylglucosamine-1-phosphate uridyltransferase/glucosamine-1-phosphate acetyltransferase	6.791	0.023320366
tig	trigger factor	6.779	0.002342885
rpsL	30S ribosomal protein S12	6.777	0.023624163
STM1190	hypothetical protein	6.593	0.009822483
rplC	50S ribosomal protein L3	6.494	0.00093819
feoB	ferrous iron transport protein B	6.477	0.011312967
rimM	ribosome maturation factor RimM	6.447	0.018849023
rplQ	50S ribosomal protein L17	6.378	0.00811738
cspE	RNA chaperone	6.292	0.045091566
rpsB	30S ribosomal protein S2	6.246	0.003047762
ahpC	alkyl hydroperoxide reductase subunit C	6.212	0.008488904
entA	2,3-dihydro-2,3-dihydroxybenzoate dehydrogenase	6.208	0.016049343
tuf	elongation factor Tu	6.111	2.80E-09
rpoA	DNA-directed RNA polymerase subunit alpha	6.047	2.05E-07
dapD	2,3,4,5-tetrahydropyridine-2,6-dicarboxylate N-succinyltransferase	6.034	0.032763913
rpoS	RNA polymerase sigma factor RpoS	5.852	3.17E-06

rplU	50S ribosomal protein L21	5.674	0.032468576
rnpB	miscRNA	5.663	5.84E-20
deoB	phosphopentomutase	5.312	0.020485435
deaD	ATP-dependent RNA helicase DeaD	5.203	0.043492603
vacB	ribonuclease R	5.027	0.031164167
ilvC	ketol-acid reductoisomerase	4.984	0.030896205
secY	preprotein translocase subunit SecY	4.911	2.27E-05
rpsC	30S ribosomal protein S3	4.882	0.013655347
rplD	50S ribosomal protein L4	4.810	0.034275804
fkpA	FKBP-type peptidyl-prolyl cis-trans isomerase	4.513	0.047356231
rpsE	30S ribosomal protein S5	4.501	0.049723074
sodA	superoxide dismutase	4.407	0.049997847
rplB	50S ribosomal protein L2	4.342	0.023540741
fliC	flagellin	4.155	0.000869877
glpD	glycerol-3-phosphate dehydrogenase	3.939	0.004697577
groEL	chaperonin GroEL	3.176	1.38E-06
**Downregulated genes (1)**			
STM1513	cytoplasmic protein	-200.975	4.01E-07

[1] Fold change indicate expression of bacterial transcripts between the infected Flight cultures relative to infected Ground cultures. Significance determined according to an FDR < 0.05. Log2-fold change values were converted to fold change.
