# Supplementary material for: Evaluating the effect of spaceflight on the host–pathogen interaction between human intestinal epithelial cells and Salmonella Typhimurium
Source: NPJ Microgravity. 2021 Mar 9;7:9. doi: 10.1038/s41526-021-00136-w (PMC7943786; doi:10.1038/s41526-021-00136-w)
Supplement: Supplementary file 3 — Reporting Summary Checklist [file 41526_2021_136_MOESM3_ESM.pdf]

nature research

Corresponding author(s): Cheryl A. Nickerson

Last updated by author(s): Jan 27, 2021

Reporting Summary

Nature Research wishes to improve the reproducibility of the work that we publish. This form provides structure for consistency and transparency in reporting. For further information on Nature Research policies, see our Editorial Policies and the Editorial Policy Checklist.

Statistics

For all statistical analyses, confirm that the following items are present in the figure legend, table legend, main text, or Methods section.

n/a	Confirmed	
☐	☒	The exact sample size (n) for each experimental group/condition, given as a discrete number and unit of measurement
☐	☒	A statement on whether measurements were taken from distinct samples or whether the same sample was measured repeatedly
☐	☒	The statistical test(s) used AND whether they are one- or two-sided<br>*Only common tests should be described solely by name; describe more complex techniques in the Methods section.*
☒	☐	A description of all covariates tested
☐	☒	A description of any assumptions or corrections, such as tests of normality and adjustment for multiple comparisons
☐	☒	A full description of the statistical parameters including central tendency (e.g. means) or other basic estimates (e.g. regression coefficient) AND variation (e.g. standard deviation) or associated estimates of uncertainty (e.g. confidence intervals)
☐	☒	For null hypothesis testing, the test statistic (e.g. F, t, r) with confidence intervals, effect sizes, degrees of freedom and P value noted<br>*Give P values as exact values whenever suitable.*
☒	☐	For Bayesian analysis, information on the choice of priors and Markov chain Monte Carlo settings
☒	☐	For hierarchical and complex designs, identification of the appropriate level for tests and full reporting of outcomes
☒	☐	Estimates of effect sizes (e.g. Cohen's d, Pearson's r), indicating how they were calculated

Our web collection on statistics for biologists contains articles on many of the points above.

Software and code

Policy information about availability of computer code

Data collection	No software was used.
Data analysis	Protein identification and quantification was performed with ProteinPilot 4.0 software using Paragon algorithm.<br><br>RNA integrity was analyzed using Agilent 2100 Bioanalyzer Expert Software.<br><br>RNA-seq reads for each sample were quality checked using FastQC v0.10.1 and aligned to Human GRCh38.p7 primary assembly and S. Typhimurium LT2 genome using STAR v2.5.1b. A series of quality control metrics were generated on the STAR outputs. Cufflinks v2.2.1 was used to report FPKM values (Fragments Per Kilobase of transcript per Million mapped reads) and read counts. TPM (Transcripts Per Million) was calculated by an in-house R script. Differential expression (DE) analysis was performed with EdgeR package from Bioconductor v3.2 in R 3.2.3.<br><br>DAVID 6.8 was used to perform enrichment analysis of RNA-seq data.

For manuscripts utilizing custom algorithms or software that are central to the research but not yet described in published literature, software must be made available to editors and reviewers. We strongly encourage code deposition in a community repository (e.g. GitHub). See the Nature Research guidelines for submitting code & software for further information.

Data

Policy information about availability of data

All manuscripts must include a data availability statement. This statement should provide the following information, where applicable:

- Accession codes, unique identifiers, or web links for publicly available datasets
- A list of figures that have associated raw data
- A description of any restrictions on data availability

All data generated during this study are either included in the manuscript and its Supplementary files (Supplementary Table 12) or are available from the corresponding author upon reasonable request. RNA-Seq data are available at the Gene Expression Omnibus (GEO) database (GSE156066; BioProject PRJNA656571) and NASA GeneLab (https://genelab-data.ndc.nasa.gov/genelab/accession/GLDS-323).

Field-specific reporting

Please select the one below that is the best fit for your research. If you are not sure, read the appropriate sections before making your selection.

☒ Life sciences ☐ Behavioural & social sciences ☐ Ecological, evolutionary & environmental sciences

For a reference copy of the document with all sections, see nature.com/documents/nr-reporting-summary-flat.pdf

Life sciences study design

All studies must disclose on these points even when the disclosure is negative.

Sample size	Proteomics: Proteomic analysis using iTRAQ was performed on total cellular protein from HT-29 cells from triplicate flight and ground bioreactors (N=3 for flight and ground infected and uninfected cultures).<br><br>RNA seq: RNA was extracted from flight and ground cultures using remaining samples following the proteomics processing. From the remaining samples, RNA-seq analyses were performed using duplicate flight and ground bioreactors for all conditions (N=2 biological replicates) except for the infected flight samples, which only had sufficient sample remaining to evaluate duplicate technical aliquots from a single bioreactor (i.e., N=1 biological; N=2 technical replicates).
Data exclusions	The presence of FBS in the culture media resulted in strong interference from bovine proteins; a subset of which could not be distinguished from human proteins due to their identical sequence homologies and thus were excluded from our analysis.
Replication	The study was performed in spaceflight and thus all replicates were performed during that single spaceflight. It is not easy to obtain funding for or access to the microgravity platform to replicate spaceflight experiments.
Randomization	This was an in vitro infection study - randomization does not apply.
Blinding	This was an in vitro infection study - blinding does not apply.

Reporting for specific materials, systems and methods

We require information from authors about some types of materials, experimental systems and methods used in many studies. Here, indicate whether each material, system or method listed is relevant to your study. If you are not sure if a list item applies to your research, read the appropriate section before selecting a response.

Materials & experimental systems

n/a	Involved in the study	
☒	☐	Antibodies
☐	☒	Eukaryotic cell lines
☒	☐	Palaeontology and archaeology
☒	☐	Animals and other organisms
☒	☐	Human research participants
☒	☐	Clinical data
☒	☐	Dual use research of concern

Methods

n/a	Involved in the study	
☒	☐	ChIP-seq
☒	☐	Flow cytometry
☒	☐	MRI-based neuroimaging

Eukaryotic cell lines

Policy information about cell lines

Cell line source(s)	HT-29 (HTB-38)

Authentication	This study was performed in 2010 and our cell lines were not authenticated at this time. However, we did confirm that the HT-29 cells we used at that time displayed multiple markers of intestinal epithelial differentiation (Radtke et al. 2010. PloS ONE; https://doi.org/10.1371/journal.pone.0015750).
Mycoplasma contamination	Cell lines were not tested for mycoplasma contamination at the time. However, they did not display any signs of having mycoplasma contamination before, during or just after the experiment.
Commonly misidentified lines (See ICLAC register)	No commonly misidentified cell lines were used in this study.
